# Supplementary material for: Systems glycomics of adult zebrafish identifies organ-specific sialylation and glycosylation patterns
Source: Nat Commun. 2018 Nov 7;9:4647. doi: 10.1038/s41467-018-06950-3 (PMC6220181; doi:10.1038/s41467-018-06950-3)
Supplement: Supplementary file 1 — Supplementary Information [file 41467_2018_6950_MOESM1_ESM.docx]

Supplementary Information

**Systems glycomics of adult zebrafish identifies organ specific sialylation and glycosylation patterns**

Nao Yamakawa^1,2,†^, Jorick Vanbeselaere^1,†^, Lan-Yi Chang^1,3^, Shin-Yi Yu^1^, Lucie Ducrocq^1^, Anne Harduin-Lepers^1^, Junichi Kurata^4^, Kiyoko F. Aoki-Kinoshita^4^, Chihiro Sato^2^, Kay-Hooi Khoo^3^, Ken Kitajima^2^, Yann Guerardel^1,^*

^1^ Univ. Lille, CNRS, UMR 8576 – UGSF - Unité de Glycobiologie Structurale et Fonctionnelle, F- 59000 Lille, France

^2^ Bioscience and Biotechnology Center, Nagoya University, Nagoya 464-8601, Japan

^3^ Institute of Biological Chemistry, Academia Sinica, Taipei 11529, Taiwan

^4^ Faculty of Science and Engineering, Soka University, Hachioji, Tokyo 192-8577, Japan

**Supplementary Note 1****. Analytical approach**

Several technological approaches including LC-MS and MALDI-MS of native, fluorescence-labelled and permethylated glycans provide a reliable access to glycome. Here, straight analysis of permethylated glycans by MALDI-MS/MS was favored because it provides (1) overall sensitivity increase, (2) better discrimination of isomeric structures owing to specific cleavage ^1^, (3) reliable results for quantification over a wide range of glycosylated molecules ^2–4^ and (4) a single analytical workflow for the analysis of both glycoproteins and glycolipids derived glycans ^5,6^. All MS data were acquired using standard procedures (DHB 10 mg/mL in MeOH/H_2_O for permethylated glycans) that naturally result in the generation of predominant production of sodiated adducts in positive mode for both MS and MS/MS experiment. MALDI-MS spectra for permethylated glycans always produced sodiated species and sodiated parent only produced sodiated fragment ions and vice versa and their patterns are quite distinctive. The only exception to that rule was the B ions resulting from single sialic acid cleavage that generated [M+H]^+^ adducts.

**Supplementary Note 2. Structural analysis of N-linked glycans (NGs)**

Out of the 95 complex and hybrid NGs, 52 were substituted by at least one sialic acids: 41 by Neu5Ac, 12 by neu5Gc and 5 by Kdn. Five of them were substituted by both Neu5Ac and Neu5Gc, but Kdn-substituted NGs were never substituted by another type of sialic acids (Figure 3C). Seventy four NGs were substituted by at least one fucose residue, out of which 50 present at least one fucose on a LacNAc motif, and 45 on the chitobiose core. The most prominent fragments associated to CID MS/MS of permethylated glycans are always derived from the B/Y cleavage ions of HexNAc residues. This cleavage yield informative fragments on the presence and location of LacNAc motifs (B ion at *m/z* 486) that support the common Lewis type antigens (B ion at *m/z* 660 and 834). The multiple or recurrent cleavages of non-reducing terminal LacNAc epitopes shed light on the branching pattern owing to the presence or not of a remaining O-methyl group. The most obvious use of this is the possibility to distinguish the number of antennae versus the presence of poly-LacNAc motilfs and was used here to identify bi-, tri- and tetra-antennary NGs. Then, oxonium-type elimination of the C3-substituent from the GlcNAc residue allows a discrimination of the type-1 (Gal(β1,3)GlcNAc) versus type-2 (Gal(β1,4)GlcNAc) motif, which in turn permit to identify Lewis x (Lex, Gal(β1,4)[Fuc(α1,3)]GlcNAc), Lewis a (Lea, Gal(β1,3)[Fuc(α1,4)]GlcNAc), Lewis y (Ley, Fuc(α1,2)Gal(β1,4)[Fuc(α1,3)]GlcNAc) and Lewis b (Leb, Fuc(α1,2)Gal(β1,3)[Fuc(α1,4)]GlcNAc) epitopes, even from a complex mixture of NGs. Based on these, we exclusively observed type-2 LacNAc motifs and corresponding Lex epitope. No indication of type-1, Lea or Leb epitopes could be gathered so far. Positioning of Fuc residues on either LacNAc branches or chitobiose core were easily distinguished owing to the presence of fucosylated LacNAc based-B ions (*m/z* at 660, 864, 1225, 1255) on one hand or the presence of fucosylated Man_3_GlcNAc_2_ Z-ion (*m/z* at 1303 and 1317) as well as the Y_1_ ion at *m/z* 464 resulting from the cleavage of the fucosylated chitobiose core. High proportion the complex NGs (55 out of 86) showed Z-type fragments at *m/z* 690, 864, 1225 or 1255 that do not fit with usual LacNAc derived epitopes. Using a combination of MS/MS fragmentation, enzymatic digestion, chemical degradation and GC/MS linkage analysis we have previously demonstrated that these fragments were all derived from the Gal(β1,4)Gal(β1,4)GlcNAc motif that could be further fucosylated and sialylated to generate the unusual and so far zebrafish-specific Gal(β1,4)(NeuAc/Gc(α2,3)]Gal(β1,4)[Fuc(α1,3)]GlcNAc epitope^5,6^. As shown in Supplementary Figure 14, single NGs can be either substituted exclusively by Neu5Ac, Neu5Gc or a by combination of Neu5Ac and Neu5Gc. In addition, five NGs substituted by one or two Kdn residues were identified at *m/z* 2809 (NG50 and NG51), 2983 (NG61), 3129 (NG74) and 3187 (NG78). Kdn residue appear to be exclusively localized on the terminal Gal residue of the branch LacNAc, as shown by the observation of B ion at *m/z* 806 and C ion at *m/z* 579 in all corresponding fragmentation patterns (Supplementary Figure 15).

Finally, substitution by HexNAc_2_ motifs, tentatively assigned to LacDiNAc (GalNAc(β1,3/4)GlcNAc) motif, could be observed in 10 NGs (NG11, NG14-15, NG17, NG24, NG28, NG43, NG58-59, NG63) according to successive Z-type loss of external (M-259) and internal HexNAc (M-245) residues. It is noteworthy that we could observe the corresponding release of B-type fragment ion resulting from cleavage of internal HexNAc residue only when the HexNAc_2_ motif was sialylated either by Neu5Ac (*m/z* 888) or Neu5Gc (*m/z* 918) but not when unsialylated. When sialylated, the sialylation exclusively occurred on the terminal non-reducing HexNAc residues in a2-6 position, as shown by the B-ions at *m/z* 643 and 673, respectively and in accordance with previous data from Hanzawa and collaborators (Supplementary Figure 16)^7^.

**Supplementary Note 3. Structural analysis of O-linked glycans (OGs)**

Mass spectrometry sequencing of OGs released by reductive β-elimination established the presence of mucin type OGs, but no O-mannosylated or O-fucosylated glycans that are known to be minor compounds in vertebrates. The nature of the OG core was inferred for each compound from the combination of GalNac-ol containing Y- and Z-type fragments, in particular the cleavage of Gal(β1,3)GalNAc-ol and Sia(α2,6)GalNAc-ol linkages for core-1 (Gal(β1,3)GalNAc-ol) as well as Gal(β1,3)GalNAc-ol and GlcNAc(α1,6)GalNAc-ol linkages for core-2 (Gal(β1,3)[GlcNAc(β1,6)]GalNAc-ol). Altogether, about 80% of identified OGs exhibited a core-1 and 15% a core-2. A single low intensity core-3 (GlcNAc(β1,3)GalNAc-ol) OG OG6 at *m/z* 936 and core-4 (GlcNAc(β1,3)[GlcNAc(β1,6)]GalNAc-ol) OG14 at *m/z* 1239 were tentatively identified in gill and ovary, respectively. The unusual core-4 OG14 was clearly identified in ovary owing to multiple cleavages of HexNAc-HexNAc linkages that established the nature of the two branches as HexNAc and Fuc(α1,3)HexNAc-HexNAc (Supplementary Figure 17). Although we have no formal proof for the identification of HexNAc residues, the wide distribution of LacDiNAc epitopes in both vertebrates and invertebrates coupled to the known biosynthetic pathways of mucin-types *O*-glycans strongly suggest that the structure OG14 is GlcNAc(β1,6)[Fuc(α1,3)GalNAc(β1,3/4)GlcNAc(β1,3)]GalNAc-ol The identification of OG6 was deduced from the MS/MS losses of terminal HexNAc residues as Y/Z-ions at *m/z* 659/677 and Neu5ac as Y-ion at *m/z* 561 which established the HexNAc-[Neu5Ac]GalNAc-ol sequence. It was tentatively assigned to GlcNAc(β1,3)[Neu5Ac(α2,6)]GalNAc-ol, but could similarly be core-5, (GalNAc(α1,3)GalNAc-ol), core-6 (GlcNAc(β1,6)GalNAc-ol) or core-7 (GalNAc(α1,3)GalNAc-ol), although these are very rarely observed.

As for NGs, the most common sialic acid observed on OGs was Neu5Ac. Indeed, among the 33 identified OGs, 24 were substituted by at least one sialic acid, including 17 substituted by Neu5Ac, 9 by Neu5Gc and 4 by Kdn (Figure 5, Supplementary Data 2). Substitution by Kdn was established owing to Y-ions at M-334 in all MS/MS spectra. Signal at *m/z* 854 was assigned as Gal(β1,3)[Kdn(α2,6)]GalNAc-ol based on the observation of M-Kdn at *m/z* 520, Hex-HexNAc-ol signal at *m/z* 520 and [M+H]^+^ and [M+Na]^+^ Kdn signals at *m/z* 335 and 357. Absence of Kdn-Hex B-ion signal at *m/z* 579 makes it very unlikely that Kdn substituted Gal residue in OG1 (Supplementary Figure 18). Signal at *m/z* 1174 was shown to contain two isomers OG10 and OG11 with Kdn_2_Hex_1_HexNAc-ol compositions that were attributed to Kdn(α2,8)Kdn(α2,6)[Gal(β1,3)]GalNAc-ol (OG10) and to Kdn(α2,3)Gal(β1,3)[Kdn(α2,6)]GalNAc-ol (OG11). The Kdn-HexNAc-ol and Kdn-Hex fragments from OG11 were unambiguously identified owing to C/Z ions pair generated by the cleavage of the Gal(β1,3)GalNAc-ol motif at *m/z* 579 and 618, whereas Kdn dimer was identified in OG10 owing to the observation of the Kdn_2_ fragment at *m/z* 677 and of the secondary cleavage of Kdn(α2,6)GalNAc-ol motif at *m/z* 604 (Supplementary Figure 18). The substitution position of Kdn could not be *de novo* established, but was tentatively attributed as (α2,8) based on previous identification of Neu5Ac(α2,8)Neu5Ac linkage in zebrafish OGs^8^. As commonly found in vertebrates, brain glycans are highly sialylated and present a higher proportion of oligosialylated OGs than any other organ. Irrespective of the nature of the sialic acid, the sialylation occurred either on the C6 of GalNAc-ol, on the C3 of Gal residue or on another sialic acid, presumably through an α2-8 linkage as previously established, to generate a polysialic acid sequence^8^. Only Kdn was not identified within a polysialylated sequence (Figure 5B). Another shared feature with NGs is the presence of Gal(β1,4)Gal motif that was identified in core-2 glycans within the zebrafish specific Gal(β1,4)Gal(β1,4)[Fuc(α1,3)]GlcNAc (OG23 at *m/z* 1361) and the Gal(β1,4)[Neu5Ac/Gc(α2,3)]Gal(α1,4)[Fuc(α1,3)]GlcNAc (OG30/31 at *m/z* 1722/1752) motifs (Supplementary Figure 17). This strongly suggests that the set of enzymes involved in the synthesis of zebrafish epitopes can use both N- and OGs as substrates. The GalNAc(β1,4)Gal(β1,3)GalNAc-ol sequence which structure was previously described in the Fuc(α1,3)GalNAc(β1,4)[Neu5Ac/Gc(α2,3)]Gal(β1,3)GalNAc-ol OGs by a combination of NMR and MS/MS in zebrafish embryo was again identified in many OGs isolated from several organs ^9^. It was associated to non-sialylated glycans (as in OG7 at *m/z* 953), non-fucosylated glycans (as in OG9 at *m/z* 1141) or fully sialylated and fucosylated (as in OG18 and OG20 at *m/z* 1315 and 1345) as previously described (Supplementary Figure 17c and 17d) ^9^. Ten OGs out of 33 were shown to be substituted by at least one fucose residue for more than 80% associated to Fuc(α1,3)GalNAc(β1,4)[Neu5Ac/Gc(α2,3)]_0-1_Gal and Gal(β1,4)[Neu5Ac/Gc(α2,3)] _0-1_Gal(α1,4)[Fuc(α1,3)]GlcNAc motifs. Alternatively, it was associated to the terminal non-reducing residue of a HexNAc residue of LacdiNAc in two OGs observed in ovary (OG14) and gill (OG27).

**Supplementary Note 4. Structural analysis of glycosphingolipids**

Monosaccharide composition analysis by GC/MS and LC/MS of total glycolipids established the presence of galactose (Gal), glucose (Glc), *N*-acetylglucosamine (GlcNAc), *N*-acetylgalactosamine (GalNAc), fucose (Fuc) and sialic acids (Neu5Ac, Neu5Gc and Kdn) (data not showed). The nature and of sphingoid bases was established by GC/MS analysis following methanolysis and TMS derivatization GSLs. In particular, d18:0 sphinganine, C18:1 sphingosine and t18:0 phytosphingosine could be distinguished owing to their specific retention times and EI/MS fragmentation patterns. Overall, the analysis of the lipid moiety by GC/MS showed that the ceramide moiety of GSLs was mainly constituted by a combination of sphingosine and phytosphingosine with C16:0, C18:0 and C24:1 fatty acyl chains.

CID MS/MS fragmentation of permethylated GSLs cleaves the Hex1-1’Cer linkage which permits to establish the composition of intact ceramides and thus evaluate the heterogeneity of lipid moiety associated to oligosaccharides. Overview of the 172 identified compounds established that the GSLs were substituted by a heterogeneous combination of sphingoide bases and fatty acids, with three major combinations identified as [d18:1, 16:0] at *m/z* 548, [d18:1, 24:1] at *m/z* 658 and [d18:1, 24:0] at *m/z* 660. However, the ceramide composition, as well as the carbohydrate composition, was to a certain extent species-specific.

The Gala series was represented by four different glycan moieties Gal(β1,1’)Cer (G1), Neu5Ac(α2,3)Gal(β1,1’)Cer (NAcGM4, G2), Neu5Gc(α2,3)Gal(β1,1’)Cer (NGcGM4, G3) and Kdn(α2,3)Gal(β1,1’)Cer (KdnGM4, G4) that exhibited a wide lipid heterogeneity. The Hemato series was composed of lactoceramide (LacCer) that could be further substituted by one, two or three sialic acids, presumably substituted in (α2,8). Mono- and di-sialylated LacCer (GL55-59/G6 and GL60-62/G7) were exclusively substituted by Neu5Ac, but trisialylated LacCer (GT4, GL63-66, G8-11) could also incorporate a single Neu5Gc residue in all three positions of the oligosialylated chain. The presence of a homogenous Neu5Ac_3_ linear chain was easily established by the observation of Y-type fragments at *m/z* 1024 [LacCer], *m/z* 1385 [Neu5Ac_1_LacCer] and *m/z* 1746 [Neu5Ac_2_LacCer] and of B-type fragments at *m/z* 376 [Neu5Ac_1_], *m/z* 759 [Neu5Ac_2_] and *m/z* 1120 [Neu5Ac_3_], which permitted to identify GL63 at *m/z* 2123 as Neu5Ac(α2,8)Neu5Ac(α2,8)Neu5Ac(α2,3)Gal(β1,4)Glc(β1,1’)Cer^576^ (Supplementary Figure 19a). By comparison, the combination of Y-type fragments at *m/z* at 1024 [LacCer], 1385 [Neu5Ac_1_LacCer], 1415 [Neu5Gc_1_LacCer], 1746 [Neu5Gc_2_LacCer], 1776 [Neu5G_1_cNeu5Gc_1_LacCer] and of B-type fragments at *m/z* 376 [Neu5Ac_1_], 406 [Neu5Gc_1_], 759 [Neu5Ac_2_], 789 [Neu5Gc_1_Neu5Ac_1_] and 1150 [Neu5Gc_1_Neu5Ac_2_] from the parent ion at 2153 established the presence of a mixture of Neu5Gc(α2,8)Neu5Ac(α2,8)Neu5Ac(α2,3)Gal(β1,4)Glc(β1,1’)Cer^576^, Neu5Ac(α2,8)Neu5Gc(α2,8)Neu5Ac(α2,3)Gal(β1,4)Glc(β1,1’)Cer^576^ and Neu5Ac(α2,8)Neu5Ac(α2,8)Neu5Gc(α2,3)Gal(β1,4)Glc(β1,1’)Cer^576^ (Supplementary Figure 19b). The extension of Hemato series to Ganglio series generated a large family of GSLs ranging from widely distributed mono-sialylated GM2 like compounds GalNAc(β1,4)[Neu5Ac/Gc(α2,3)]Gal(β1,4)Glc(β1,1’)Cer to multisialylated GA1 substituted by up to five sialic acids. Fragmentation analyses of multisialylated compounds clearly established the presence of multiple isomers differing by the extension of the sialic acid chains and their respective positions. As an example, the MS/MS analysis of GT1 at *m/z* 2572 in brain showed that it was a mixture of Neu5Ac(α2,3)Gal(β1,3)GalNAc(β1,4)[Neu5Ac(α2,8)Neu5Ac(α2,3)]Gal(β1,4)Glc(β1,1’)Cer^576^ (GT1b, GL96) and GalNAc(β1,4)Gal(β1,3) [Neu5Ac(α2,8)Neu5Ac(α2,8)Neu5Ac(α2,3)]Gal(β1,4)Glc(β1,1’)Cer^576^ (GT1c, GL97) (Supplementary Figure 12a). Surprisingly, we identified a so far un-described isomer of pentasialylated GP1 (GL106) characterized by the substitution of the internal Gal residue by a Neu5Ac_4_ linear chain. The presence of this motif was unambiguously established owing to the fragmentation of signal at *m/z* 3294 that generated an intense series of Y/B ions at *m/z* 376/22919, 759/2558, 1120/2197 and 1482/1835 resulting from the recurrent cleavage of the Neu5Ac_4_ chain (Supplementary Figure 19c). The respective positions of the single Neu5Ac residue and the Neu5Ac_4_ chain on the terminal non-reducing and the internal Gal residues were then established owing to the Y/B ions pair at 847/2469. This compound Neu5Ac(α2,3)Gal(β1,3)GalNAc(β1,4)[[Neu5Ac(α2,8)]_3_Neu5Ac(α2,3)]Gal(β1,4)Glc(β1,1’)Cer, which we named GP1d (GL106) in accordance with the nomenclature proposed by Svennerholm, was identified along the classical GP1c (GL105) Neu5Ac(α2,8)Neu5Ac(α2,3)GalNAc(β1,4)[[Neu5Ac(α2,8)]_2_Neu5Ac(α2,3)]Gal(β1,4)Glc(β1,1’)Cer. Altogether, we identified members of GA1, GM2, GM1, GM1b, GD1a, GD1b, GT1b, GT1c, GQ1c, GP1c and GP1d within the Ganglio family. Finally, as observed for GT4, tri-sialylated GT1b (GL98-100) at *m/z* 2602 and tetra-sialylated GQ1c (GL103-104) at *m/z* 2964 appeared to be substituted by both Neu5Ac and Neu5Gc in different positions to generate multiple isomers (Supplementary Figure 12). However, Neu5Gc containing isomers were always present in smaller quantities than their respective Neu5Ac equivalents. Along Hemato and Ganglio series, we identified by MS/MS a homogeneous family of GSLs that was characterized by a pentasaccharide linear sequence HexNAc-Hex-HexNAc-Gal-Glc-Cer. These compounds were in majority sialylated on the internal galactose residue, as established by the Y ion pair at *m/z* 1470 for GL118 at *m/z* 2353 (with cer^660^) and fucosylated on the terminal non-reducing HexNAc residue as established by the recurrent B ions at m/z 434, 678 and 905 to generate the sequence deHex-HexNAc-Hex-HexNAc-[NeuAc/Gc-]Gal-Glc-Cer (GL113-121) (Supplementary Figure 20a). This particular sequence has been previously identified in salmon kidney by a combination of MS/MS and NMR as the extended Ganglio series GSLS Fuc(α1,3)GalNAc(β1,4)Gal(β1,3)GalNAc(β1,4)Gal(β1,4)Glc(β1,1’)Cer^10^. Here, we have no formal proof for establishing the monosaccharides anomeries and linkages, however the MS/MS fragmentation pattern is in total agreement with the previously established sequence. In zebrafish, analogues of this compound were identified substituted either by Neu5Ac or by Neu5Gc (GL120-121) but not by Kdn, and also identified in non-fucosylated (GL109-110) *m/z* at 2066 and 2096) and non sialylated (GL107-108 at *m/z* 1879) forms (Supplementary Figure 20b). Finally, another very heterogeneous family of GSLs with carbohydrate moieties ranging from four to thirteen monosaccharides was identified. They all have in common the HexNAc-[HexNAc-]-Hex-Hex tetrasaccharide core as observed in the simple GSL GL 123 at *m/z* 1501. From the tetrasaccharie core a single HexNAc residue may be elongated by multiple hexoses, N-acetylhexosamine and fucose residues up to thirteen monosaccharides as observed in GL 171 at *m/z* 3545 in ovary. This peculiar HexNAc_2_Hex_2_ core has been independently described in two fishes, the English sole and the stripped mullet, as the hybrid neolacto-ganglio serie (LcGgCer), in which it appears to be the major type of GSLs^11,12^. So far, its identification is restricted to teleost fishes as LcGcCer-based GSLs have never been observed in other animal branch to our best knowledge. All compounds exhibited a common pentasaccharide core structure Gal(β1,4)GlcNAc(β1,3)[GalNAc(β1,4)]Gal(β1,4)Glc that is exclusively elongated from the GlcNAc, but never from the GalNAc residue. In the mullet, the external Gal residue could be substituted by both GalNAc(β1,4) and Neu5Ac(α2,3) residues^11^, whereas it could be substituted by Gal(α1,3) in salmon^13^. Both features could be observed in zebrafish owing to the identification of GL128 at *m/z* 2066 in gill, testis and ovary and GL149 at *m/z* 2186 in ovary which MS/MS fragmentation patterns are totally compatible with previously identified Neu5Ac(α2,3)Gal(β1,4)GlcNAc(β1,3)[GalNAc(β1,4)]Gal(β1,4)Glc-Cer and GalNAc(β1,3)Gal(α1,3)Gal(β1,4)GlcNAc(β1,3)[GalNAc(β1,4)]Gal(β1,4)GlcCer (Supplementary Figure 21a). Other members of the LcGgCer showing a fucosylated HexNAc residue in terminal non reducing position was identified in several organs (GL130-148). As observed in the MS/MS fragmentation patterns of GL131, 134 and 135, the Y-type fragments at *m/z* 1274, 1330 and 1345 and the Y/Y-type secondary fragments at *m/z* 1014, 1070 and 1084 all associated to the B-type fragment at m/z 905 established the presence of the LcGg core in these GSLs (Supplementary Figure 21b). The presence of the terminal deHex-HexNAc and the internal LacNAc disaccharide were unambiguously established by the observation of B-type fragment at m/z 434 and secondary fragment at *m/z* 472. The exact nature of the terminal deHex-HexNAc motif could not be directly deduced from the MS/MS data, but the apparent wide distribution of the terminal GalNAc(β1,3) and Fuc(α1,3)GalNAc(β1,3) epitopes in GSLs from several fish species including English sole (*Parophrys vetulus*), Pacific salmon (*Oncorhynchus keta*) and striped mullet (*Mugil cephalus*) strongly suggests that these GSLs are made of the Fuc(α1,3)GalNAc(β1,3)Gal(β1,4)GlcNAc(β1,3)[GalNAc(β1,4)]Gal(β1,4)GlcCer sequence. In GL147 and 148 observed at *m/z* 2330 and 2386, this sequence could be further fucosylated on the internal GlcNAc residue, as demonstrated by the B-type fragments at 1079, 678 and 434 (Supplementary Figure 21c). The secondary fragment at *m/z* 646 generated by the loss of the terminal Fuc(α1,3)GalNAc disaccharide from the deHex_2_HexNac_2_Hex_1_ fragment at *m/z* 1079 further confirmed that the additional Fuc is not located on the terminal GalNAc residue, which strongly suggests GL147/148 are made of Fuc(α1,3)GalNAc(β1,3)Gal(β1,4)[Fucα1,3)]GlcNAc(β1,3)[GalNAc(β1,4)]Gal(β1,4)GlcCer^580/636^ sequences. The LcGgCer family comprised also 21 GSLs with 8 different carbohydrate moieties originating from the elongation of the Gal(β1,4)GlcNAc(β1,3) branch which sequences that have never been observed (GL150-171). Indeed, the Gal(β1,4)GlcNAc(β1,3) may be elongated a combination of one or two Hex, HexNAc and Fuc residues to form HexNAc-HexNAc-Hex-Hex, Hex-Hex-Hex, or HexNAc-Hex-Hex-Hex branches. Furthermore, five of the carbohydrate sequences (G44-45 and G49-51) were capped by Fuc-Fuc motifs as established by the observation of HexNAc_1_Fuc_2_ and Fuc_2_ fragments at *m/z* 630 and 403 in GL (Supplementary Figure 21d). The cleavage of Fuc-HexNAc linkage as a C-ion to generate the Fuc_2_ fragment at *m/z* 403 strongly suggests that the Fuc residue is linked in C3-position on the HexNAc, in agreement with the previously described Fuc(α1,3)GalNAc motif. However, the exact structural features of this complex series of unusual multifucosylated GSLs should be investigated through a combination of NMR, MS/MS and GC/MS analyses that is out of the scope of the present glycomics report as it requires the purification of individual GSLs prior to detailed analysis. This will be the focus of a coming publication.

Supplementary Figure 1 (1/3)


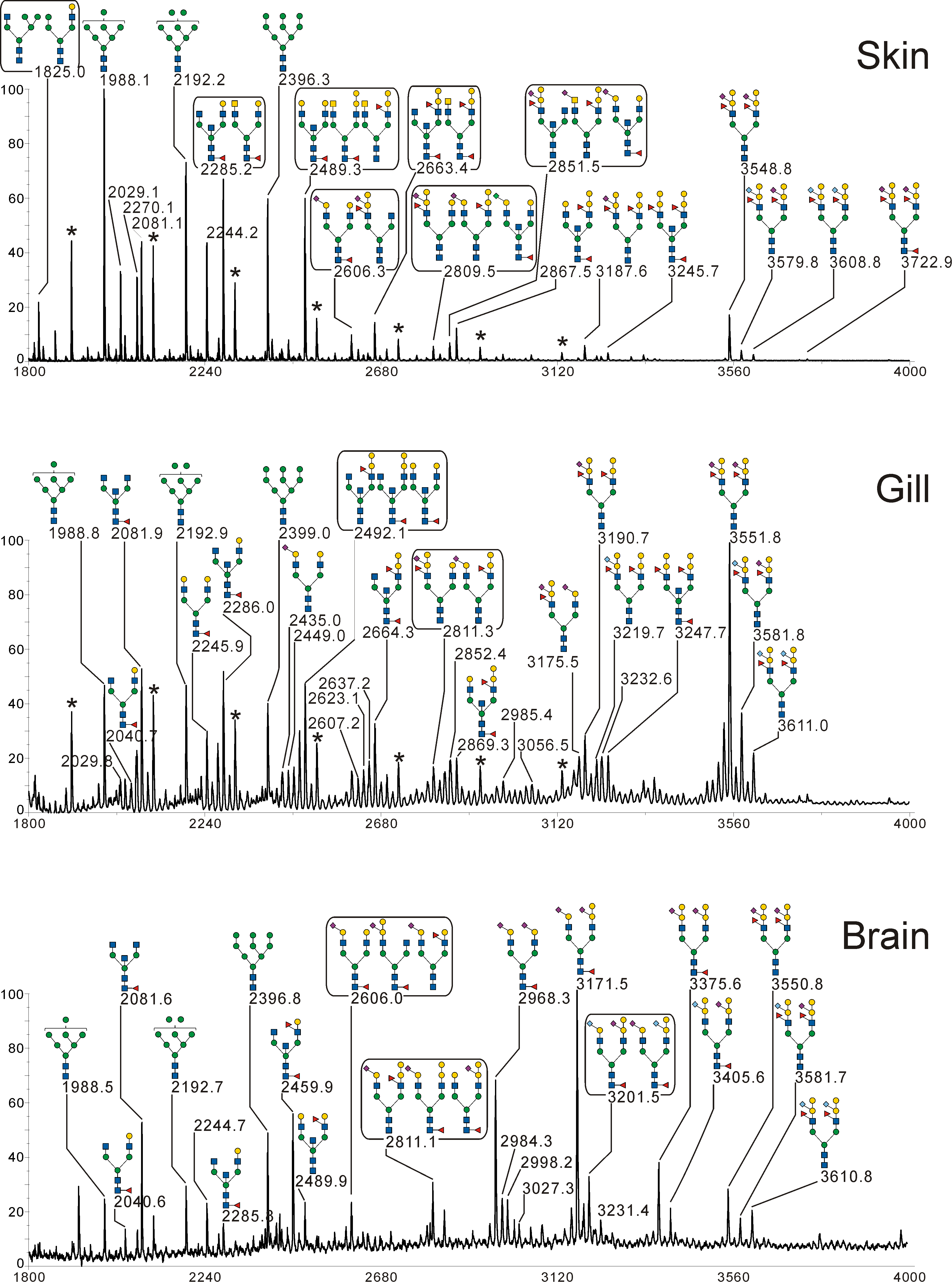


Supplementary Figure 1 (2/3)


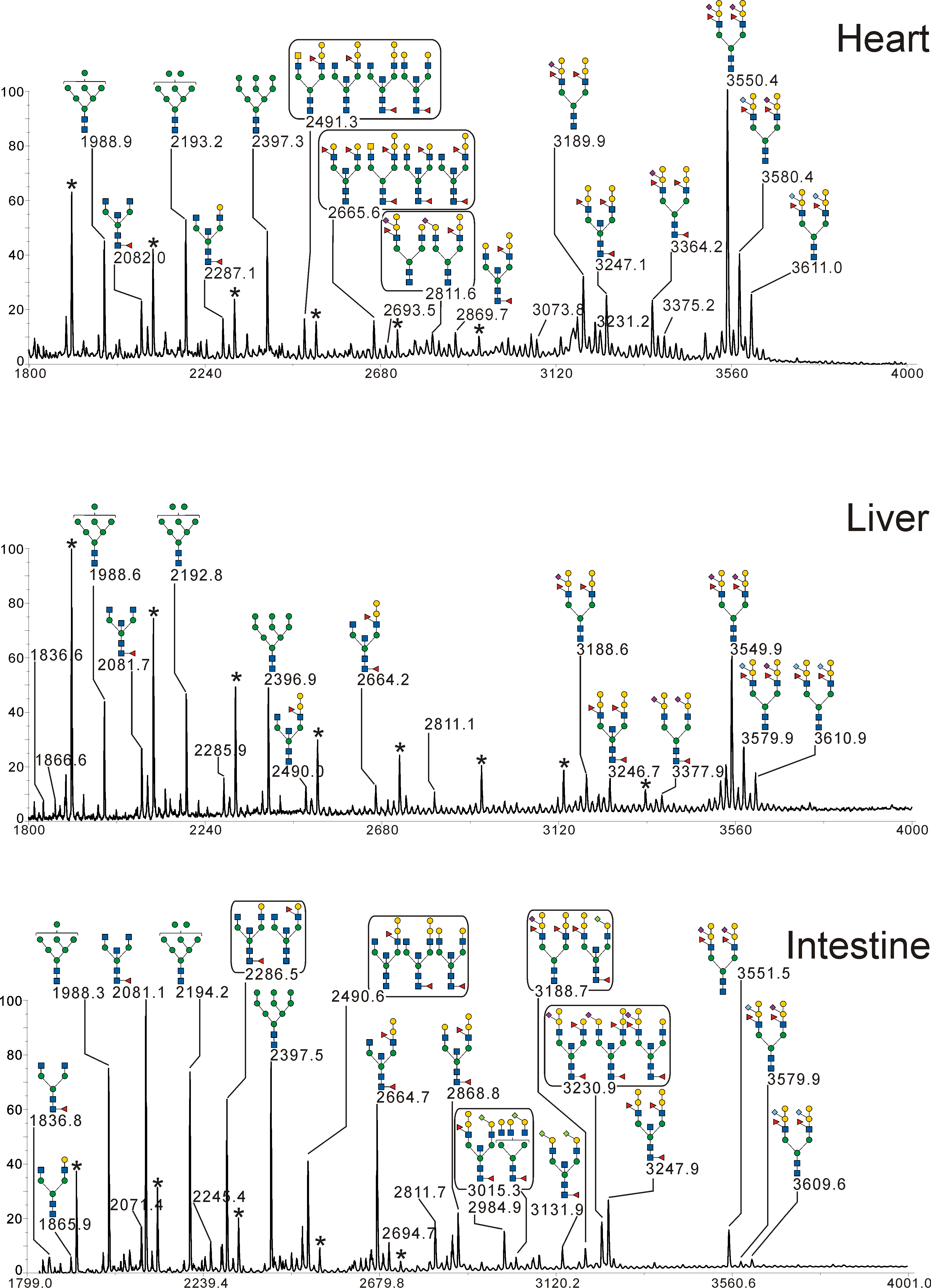


Supplementary Figure 1 (3/3)


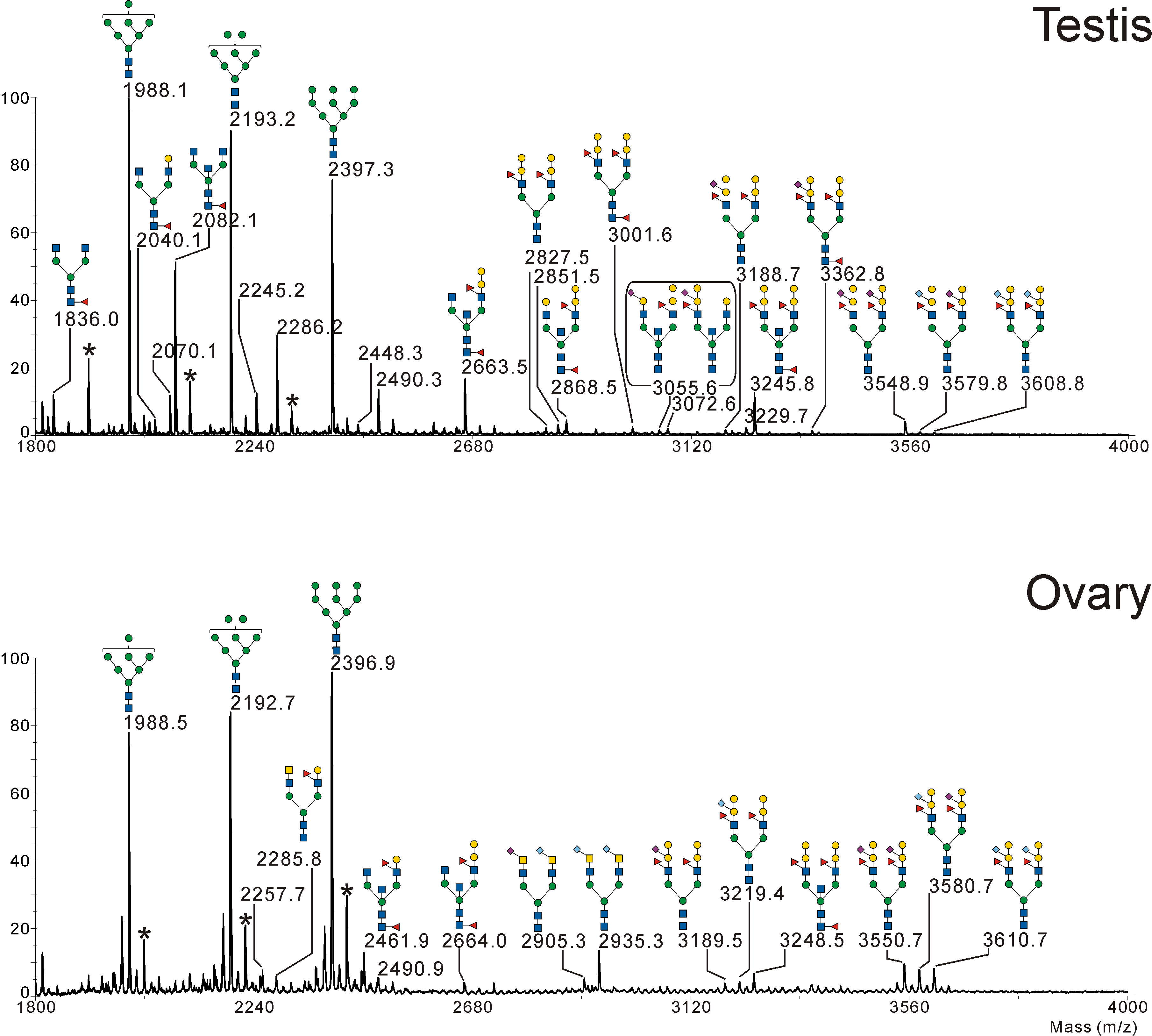


Supplementary Figure 1 - MALDI-TOF-MS spectra of permethylated NGs isolated from the eight organs. Graphical representation is based on accepted conventions for glycans and monosaccharide nomenclature as follows: yellow circle, Gal; yellow square, GalNAc; blue circle, Glc; blue square, GlcNAc; green circle, Man; red triangle, Fuc; purple diamond, Neu5Ac; light blue diamond, Neu5Gc; green diamond, Kdn.

Supplementary Figure 2 - Ratios of oligomannosylated N-glycans (in green) versus hybrid and complex-type N-glycans. NGs were quantified using the integration value of identified *m/z* signals recorded by MALDI-MS analysis of permethylated glycans and summed according to their nature.

Supplementary Figure 3 (1/4)


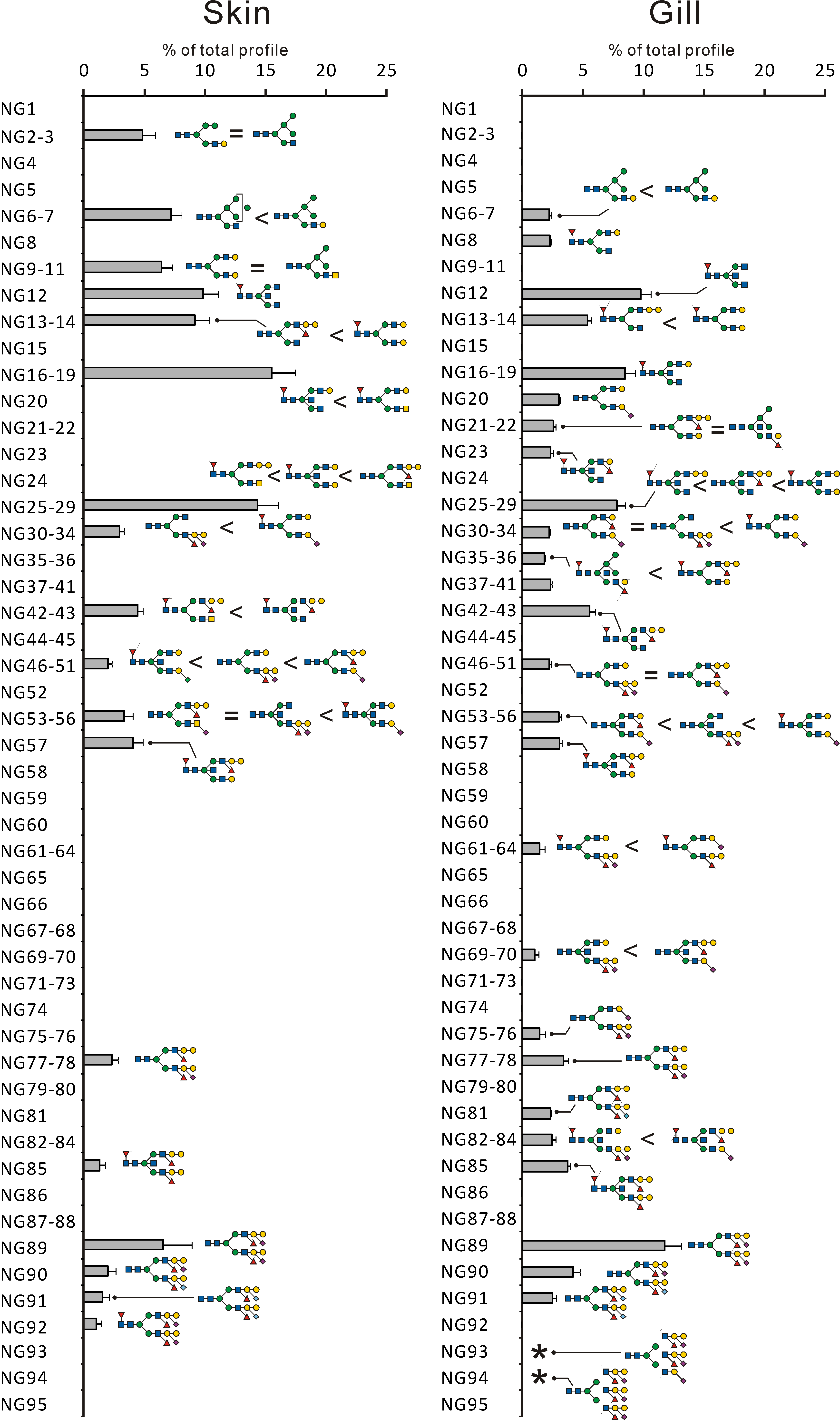


Supplementary Figure 3 (2/4)


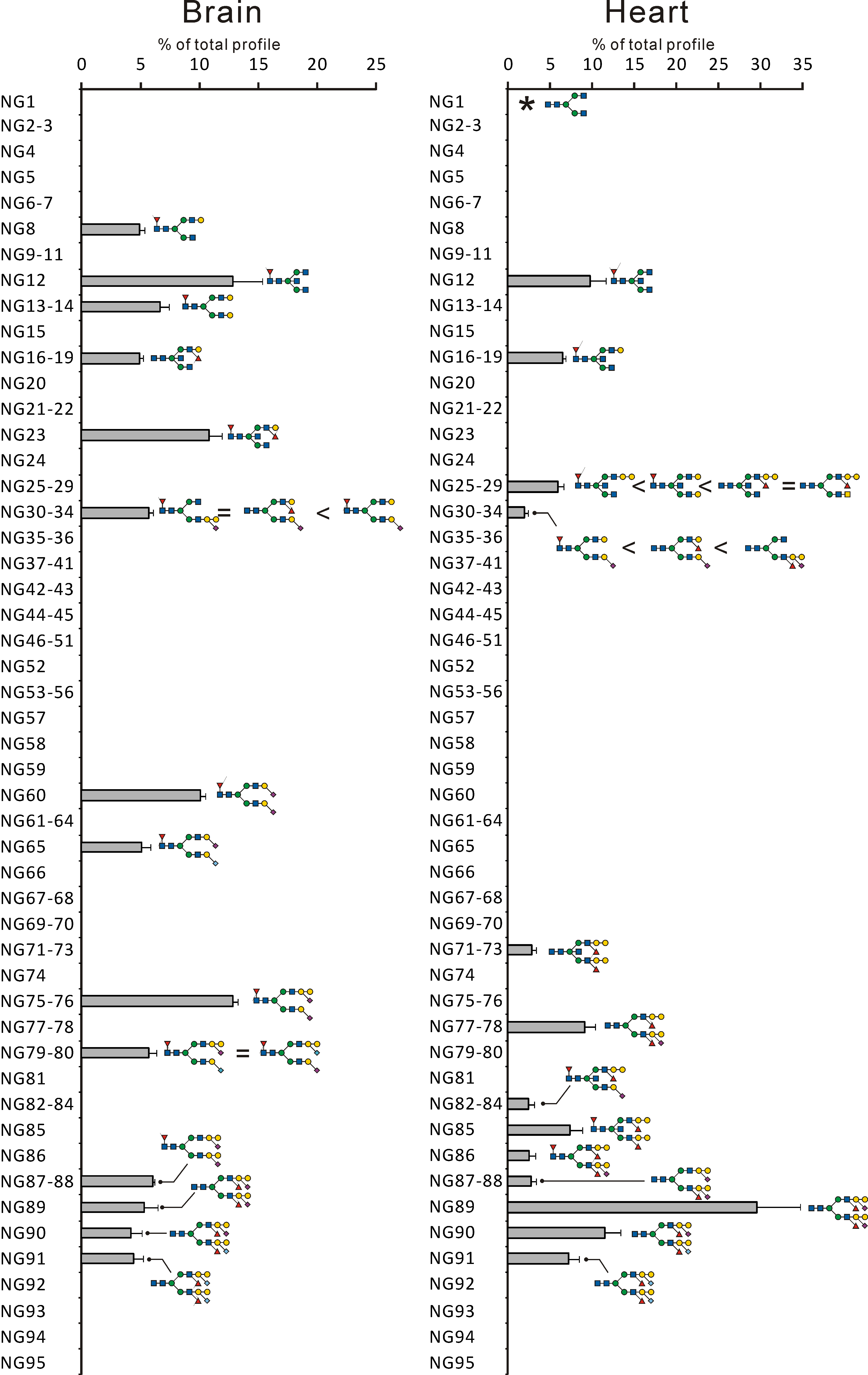


Supplementary Figure 3 (3/4)


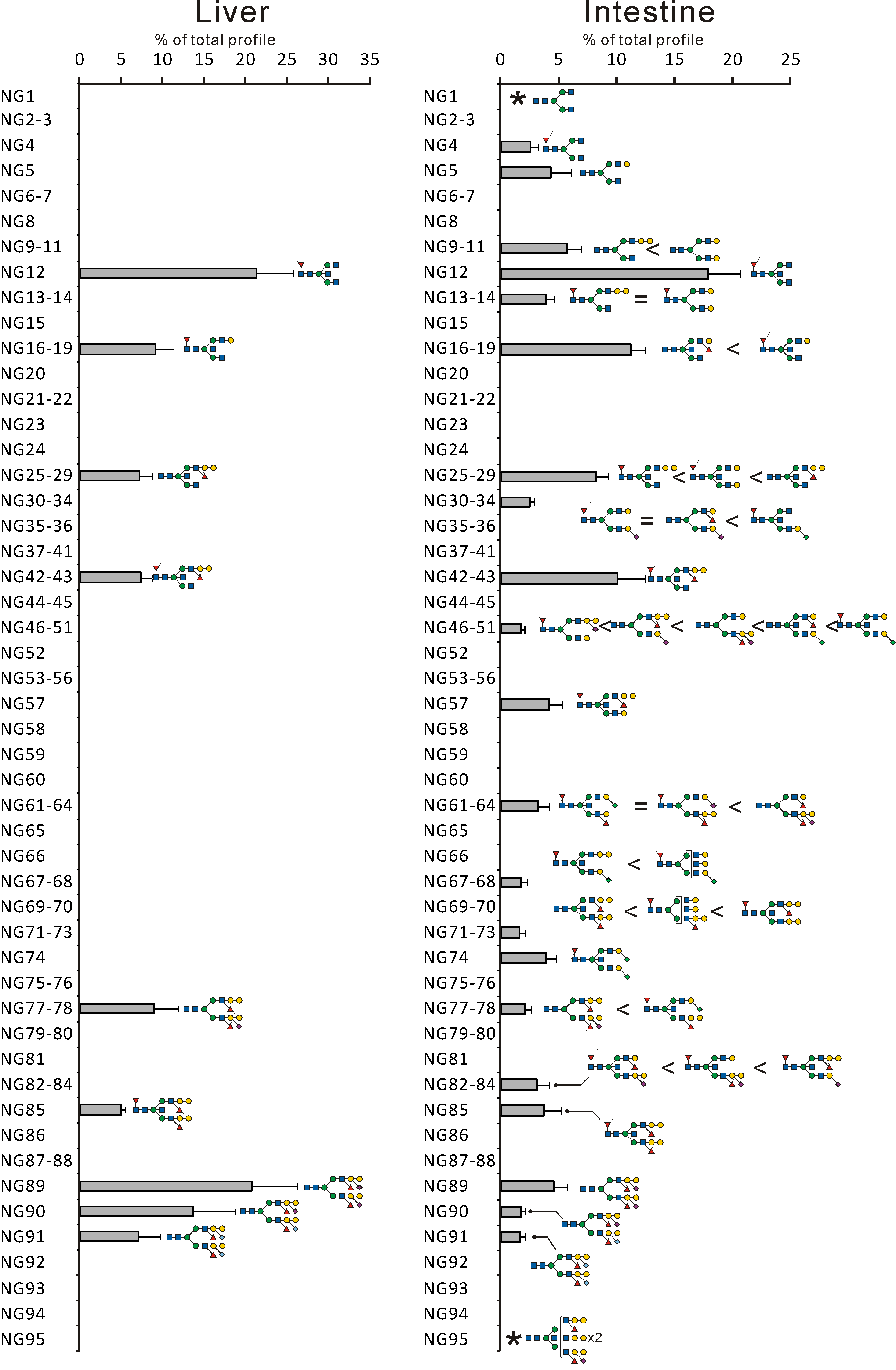


Supplementary Figure 3 (4/4)


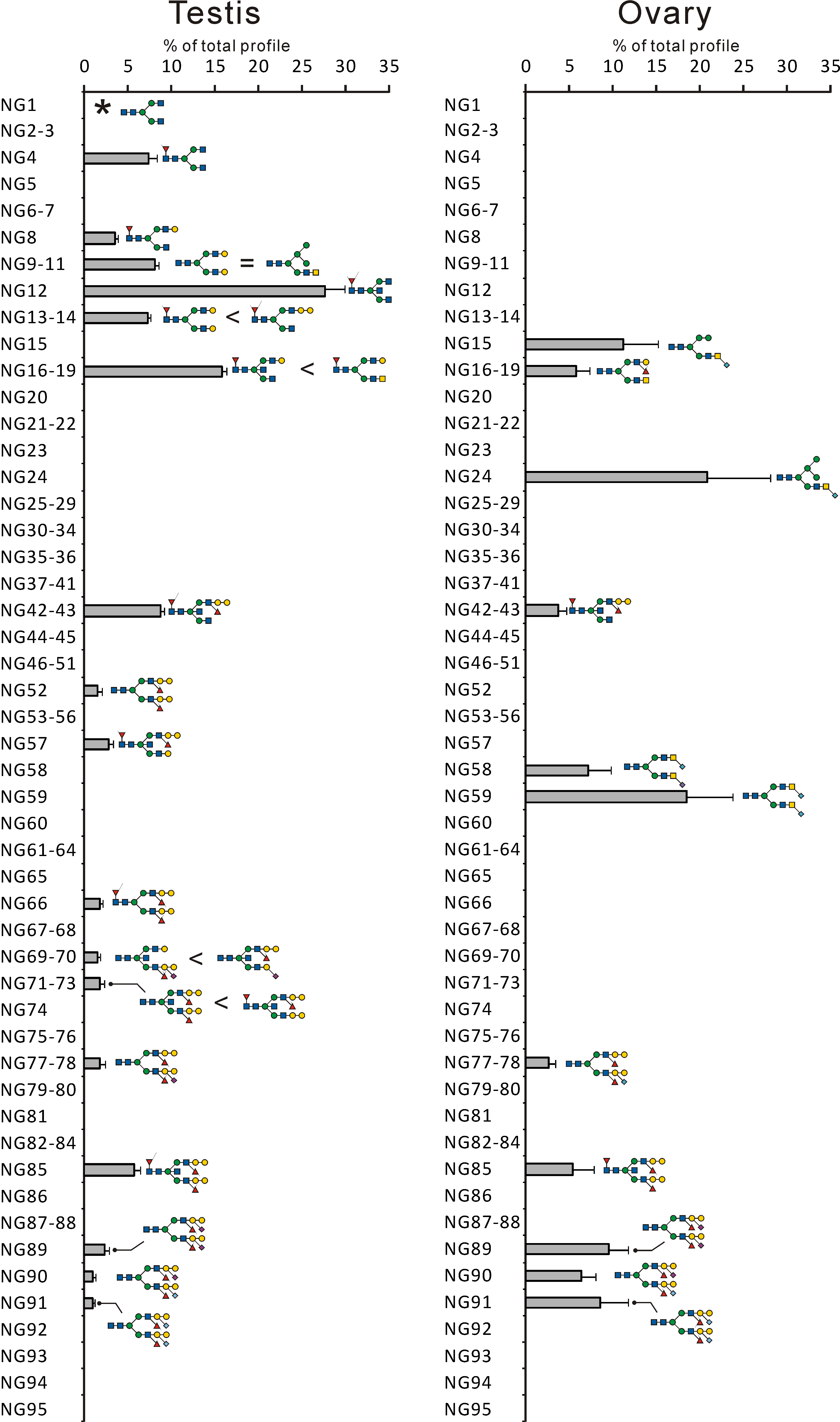


**Supplementary Figure 3** - Relative quantification of complex NGs NG1-NG92 (as described in Data 1) in eight organs. NGs were quantified using the integration value of identified *m/z* signals recorded by MALDI-MS analysis of permethylated glycans as % of the total identified signals (see Figure 3). Quantification values resulted from three independent experiments. The relative importance of isomeric structures in individual *m/z* signals was estimated by the intensity of MS/MS signals generated by MALDI-TOF/TOF fragmentation signals. Graphical representation is based on accepted conventions for glycans and monosaccharide nomenclature as follows: yellow circle, Gal; yellow square, GalNAc; blue circle, Glc; blue square, GlcNAc; green circle, Man; red triangle, Fuc; purple diamond, Neu5Ac; light blue diamond, Neu5Gc; green diamond, Kdn. Data are shown as mean ± SE of three experiments.

Supplementary Figure 4 (1/3)


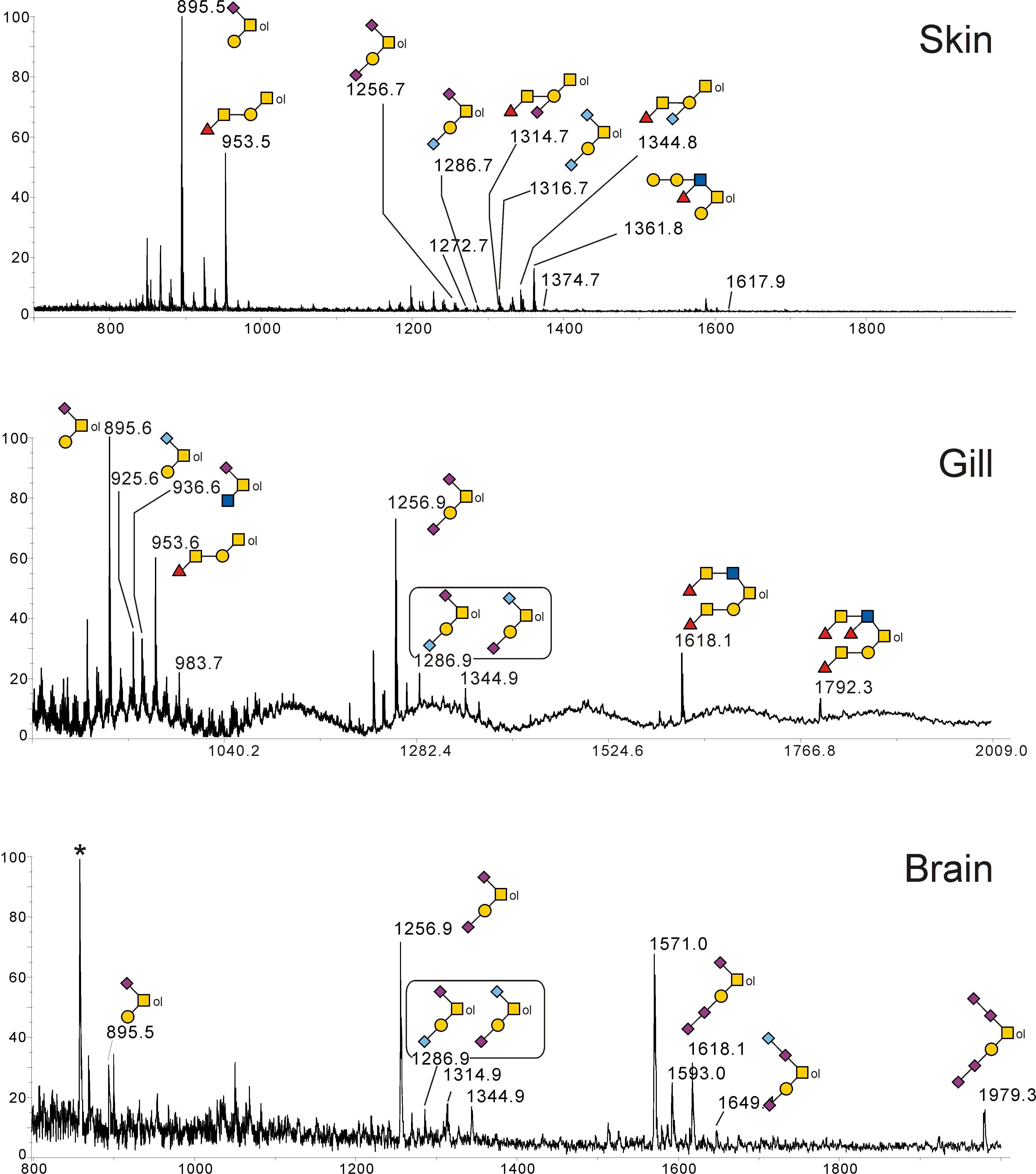


Supplementary Figure 4 (2/3)


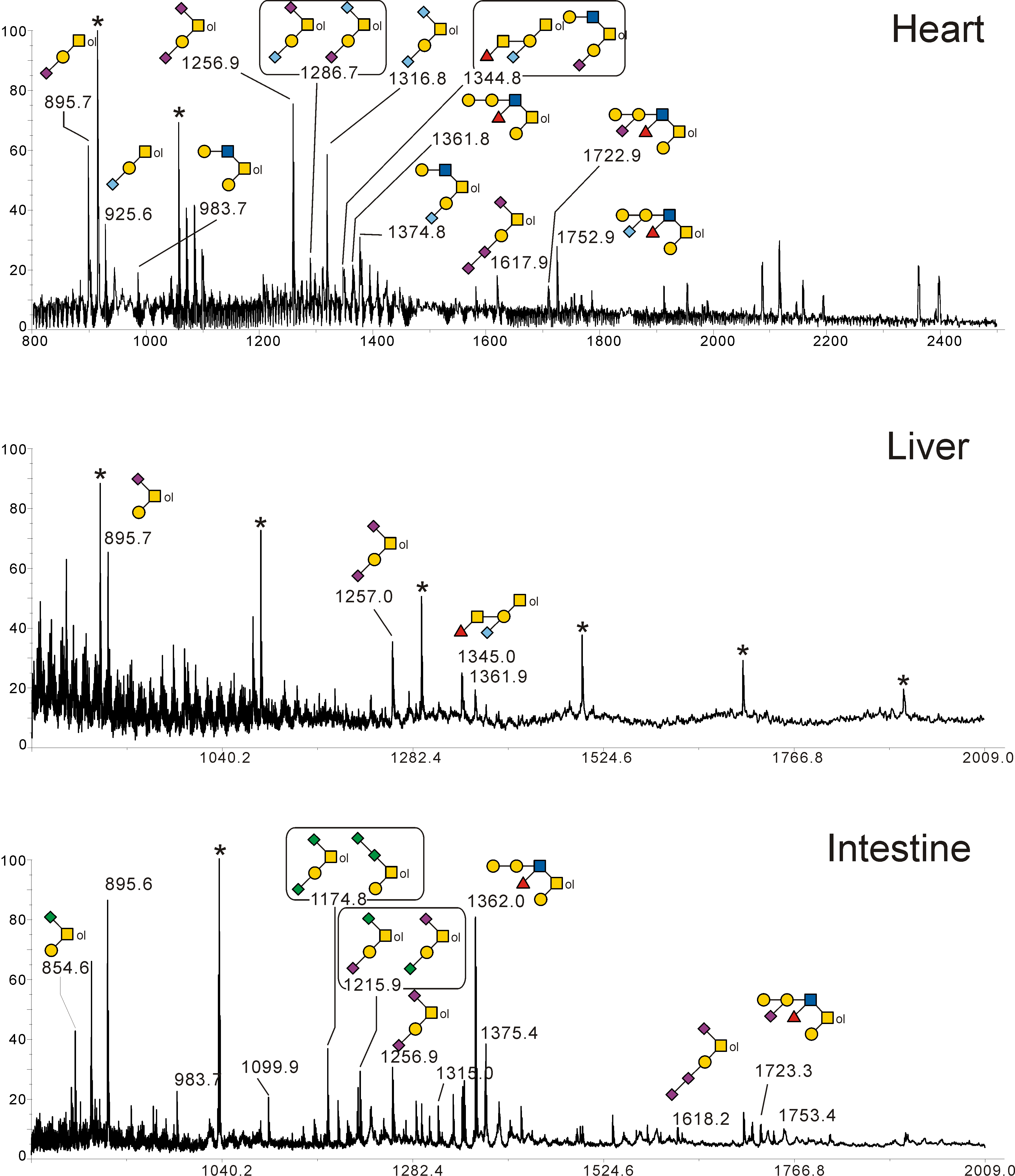


Supplementary Figure 4 (3/3)


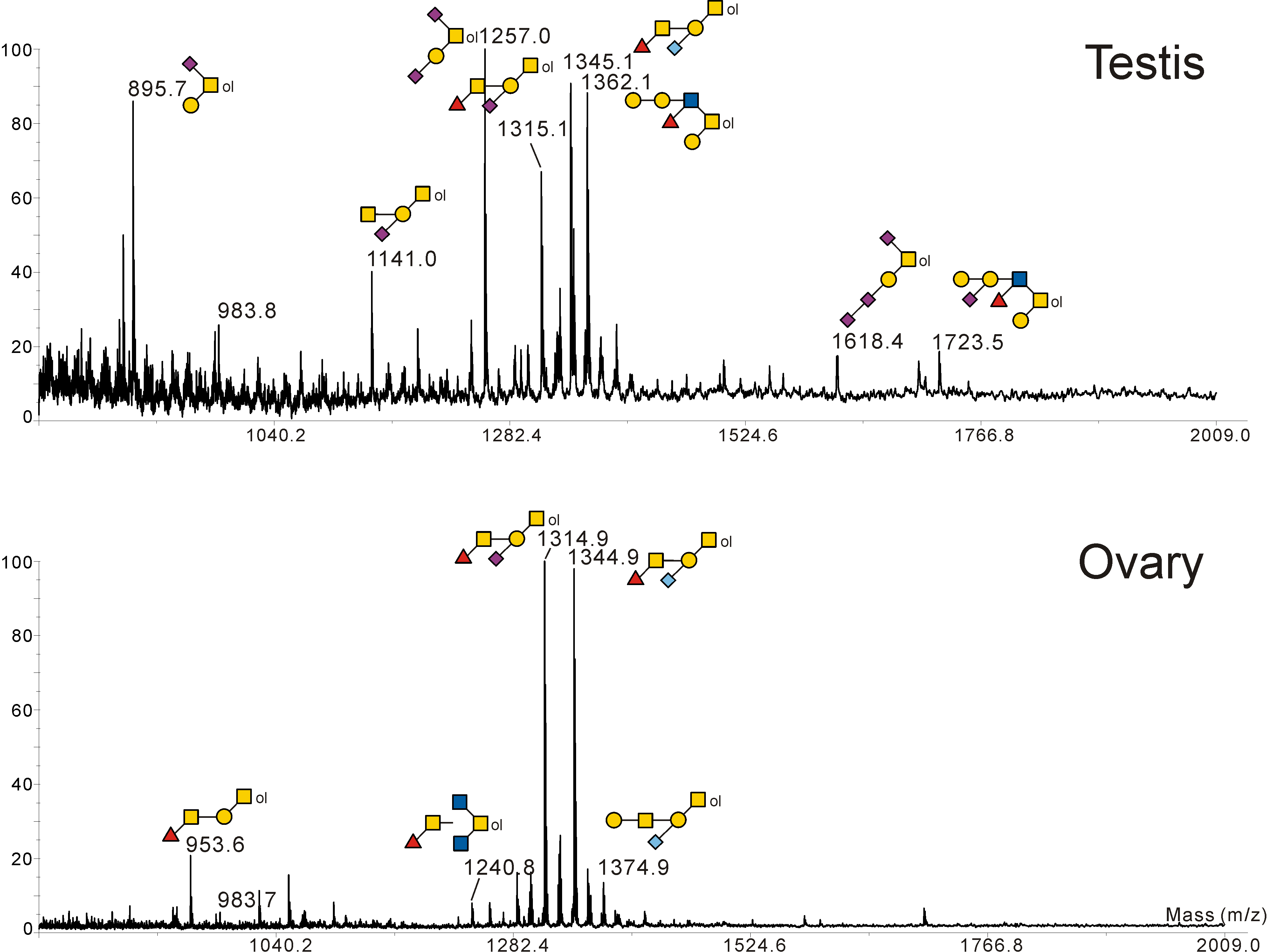


Supplementary Figure 4 - MALDI-TOF-MS spectra of permethylated OGs isolated from the eight organs. Graphical representation is based on accepted conventions for glycans and monosaccharide nomenclature.

Supplementary Figure 5 (1/4)


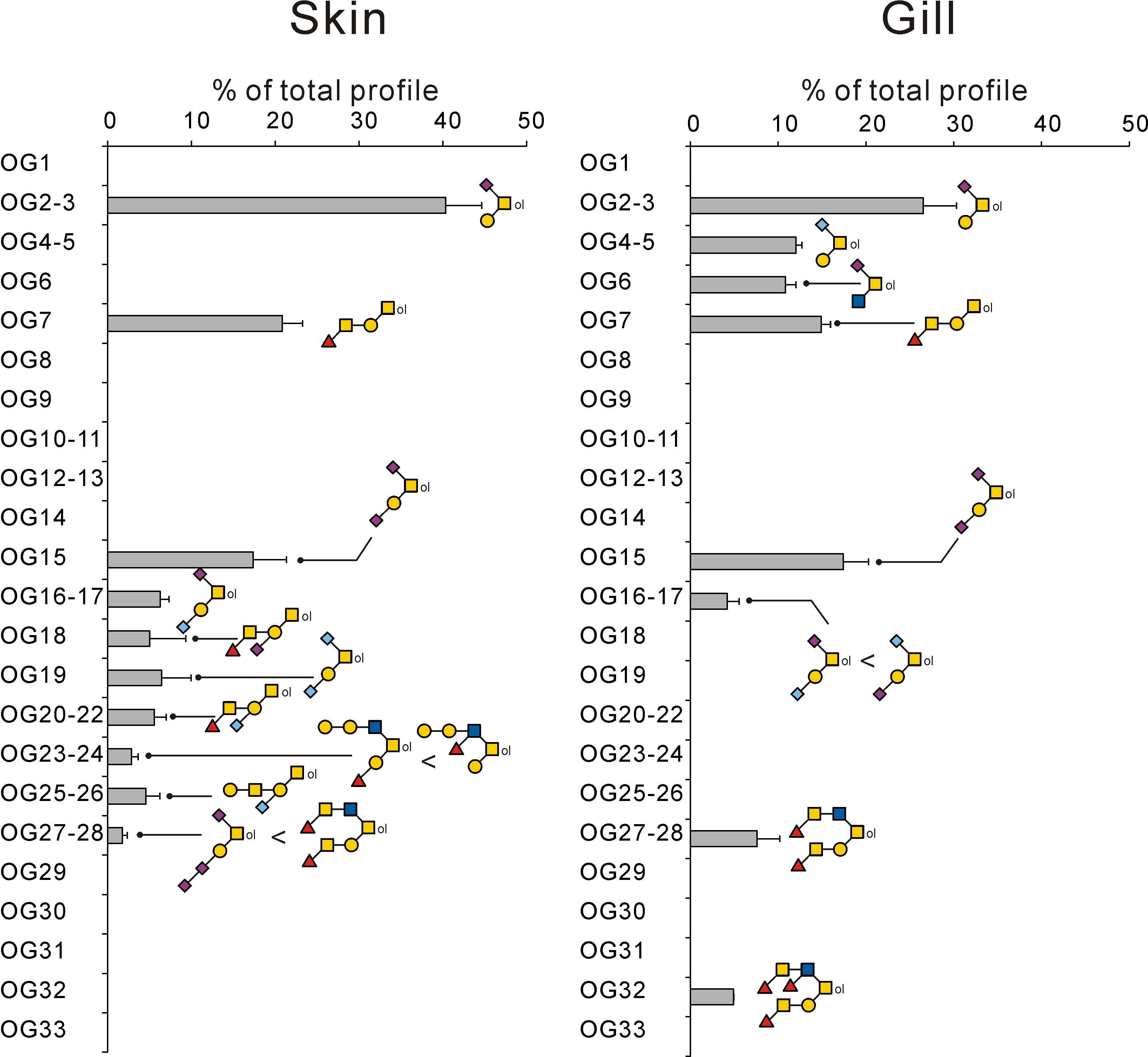


Supplementary Figure 5 (2/4)


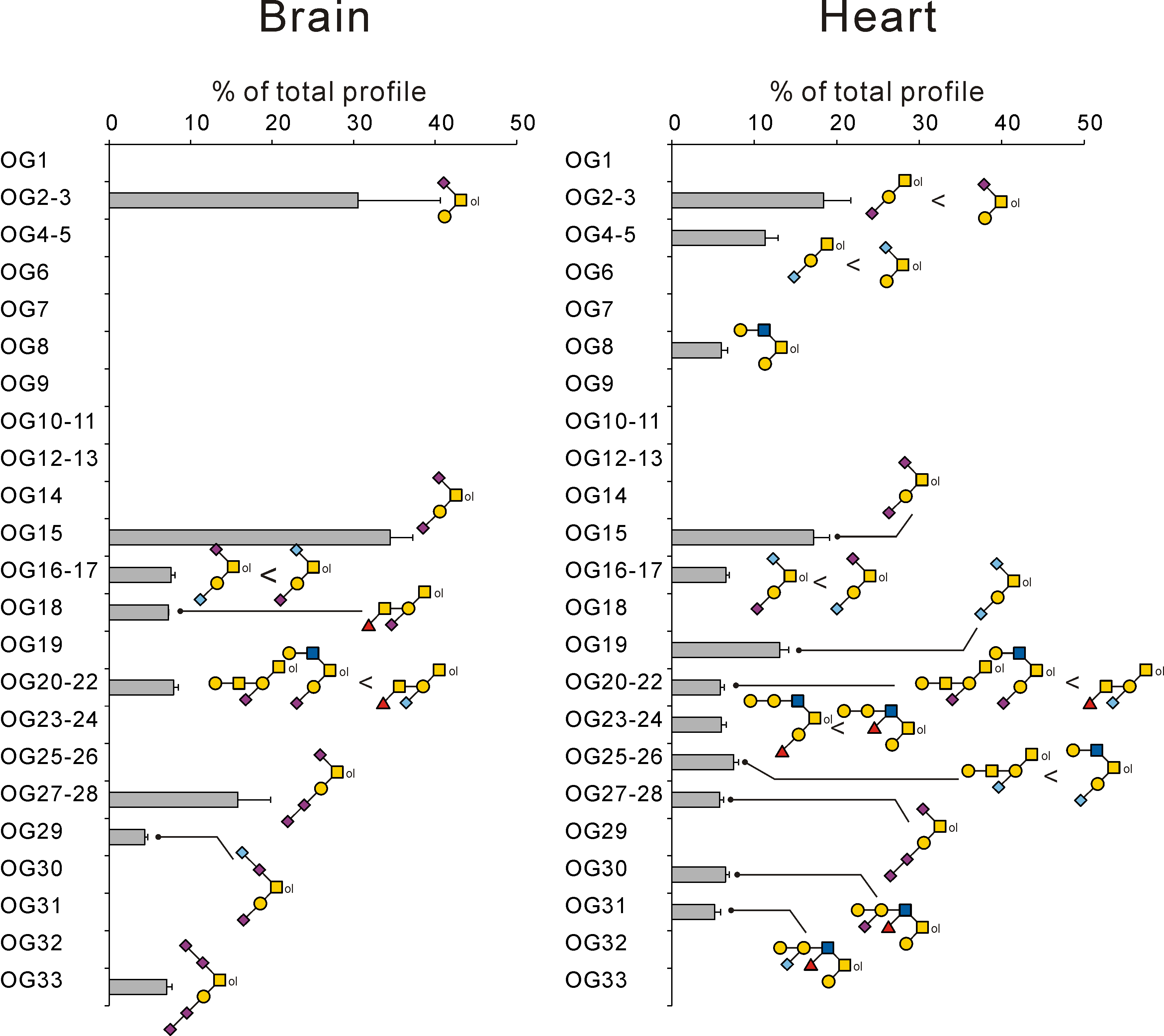


Supplementary Figure 5 (3/4)


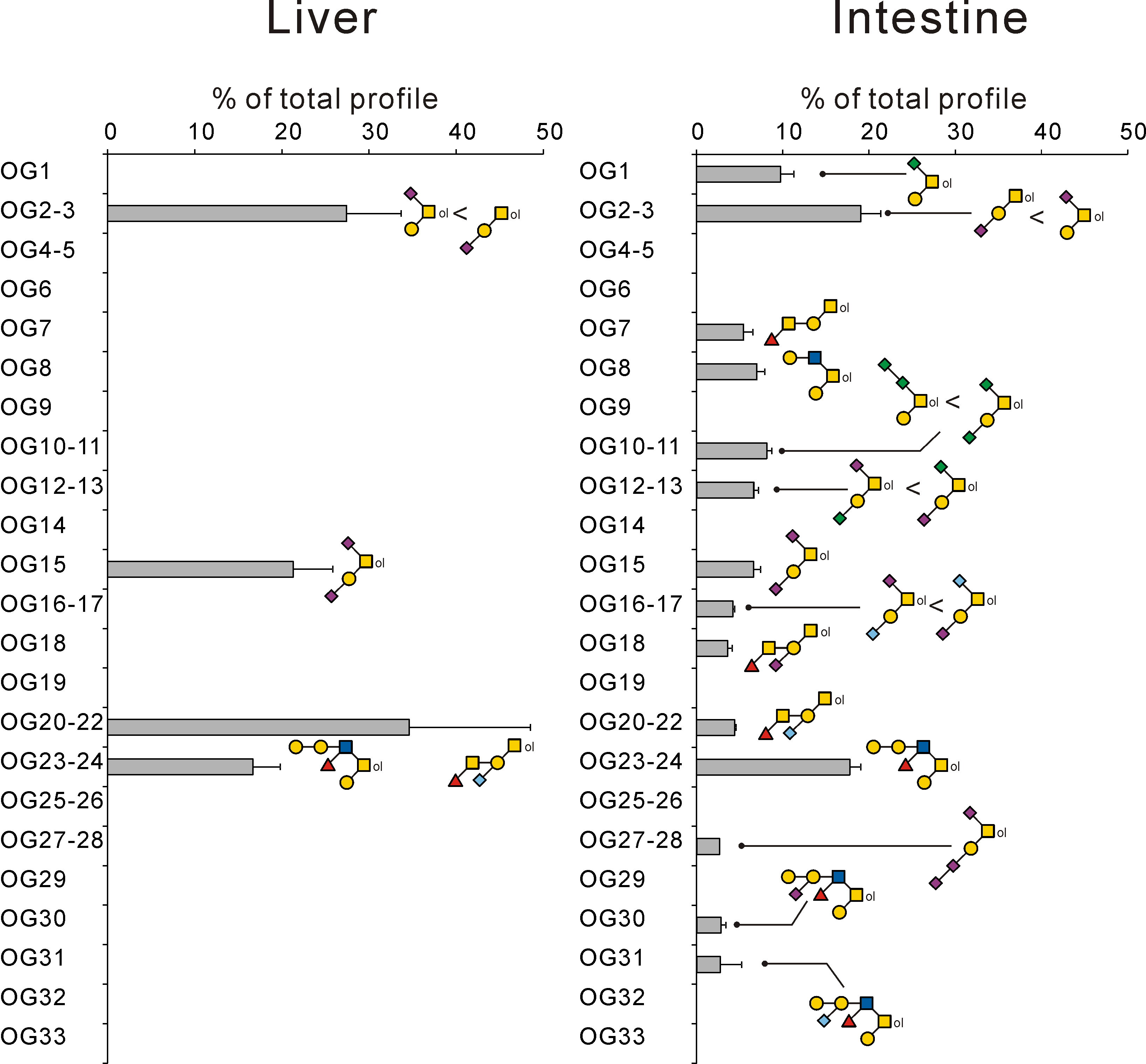


Supplementary Figure 5 (4/4)


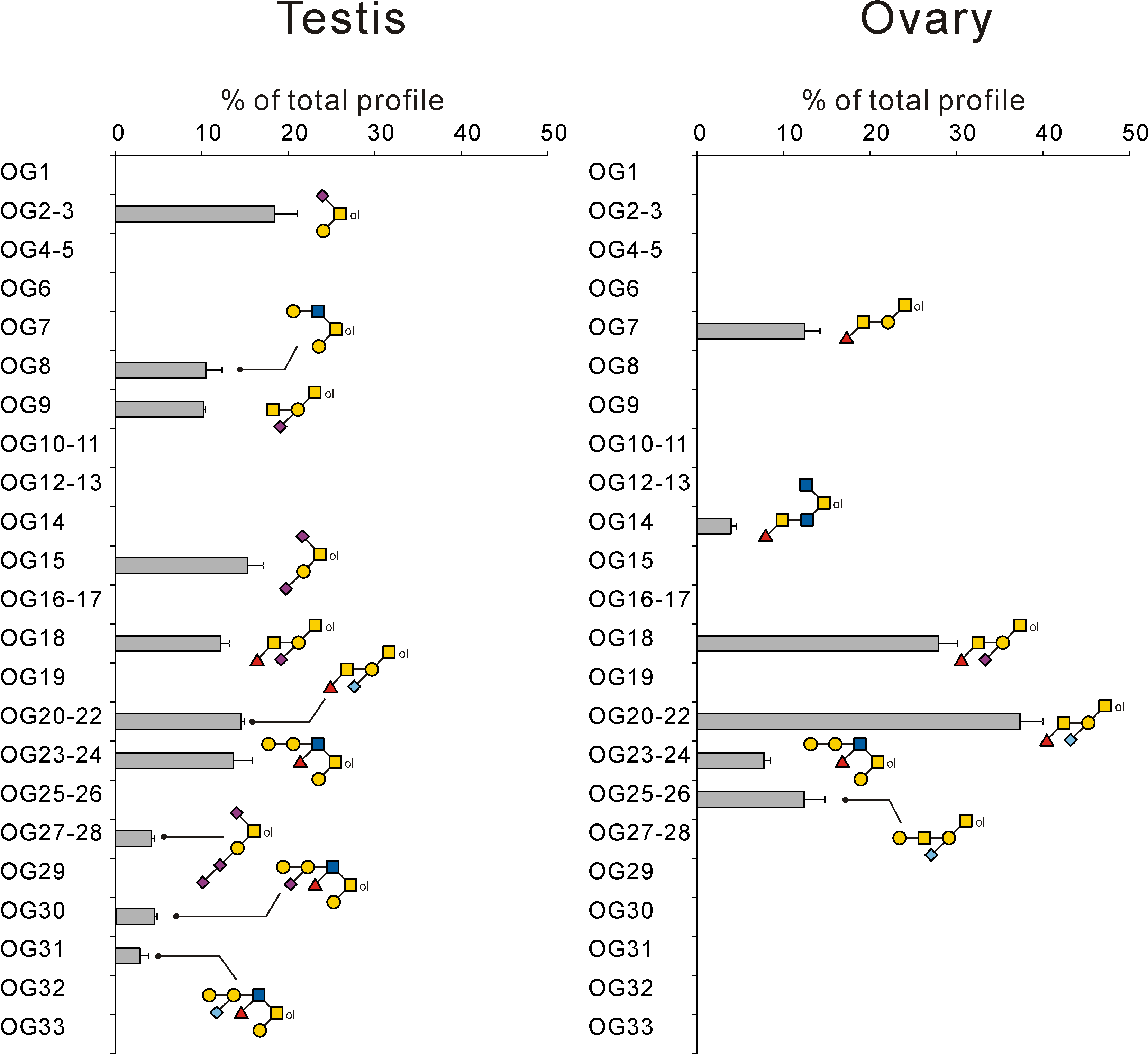


**Supplementary Figure 5** - Relative quantification of OGs in eight organs. OGs were quantified using the integration value of identified *m/z* signals recorded by MALDI-MS analysis of permethylated glycans as % of the total identified signals (see Figure 5). Quantification values resulted from three independent experiments. The relative importance of isomeric structures in individual *m/z* signals was estimated by the intensity of MS/MS signals generated by MALDI-TOF/TOF fragmentation signals. Data are shown as mean ± SE of three experiments.

Supplementary Figure 6 (1/3)


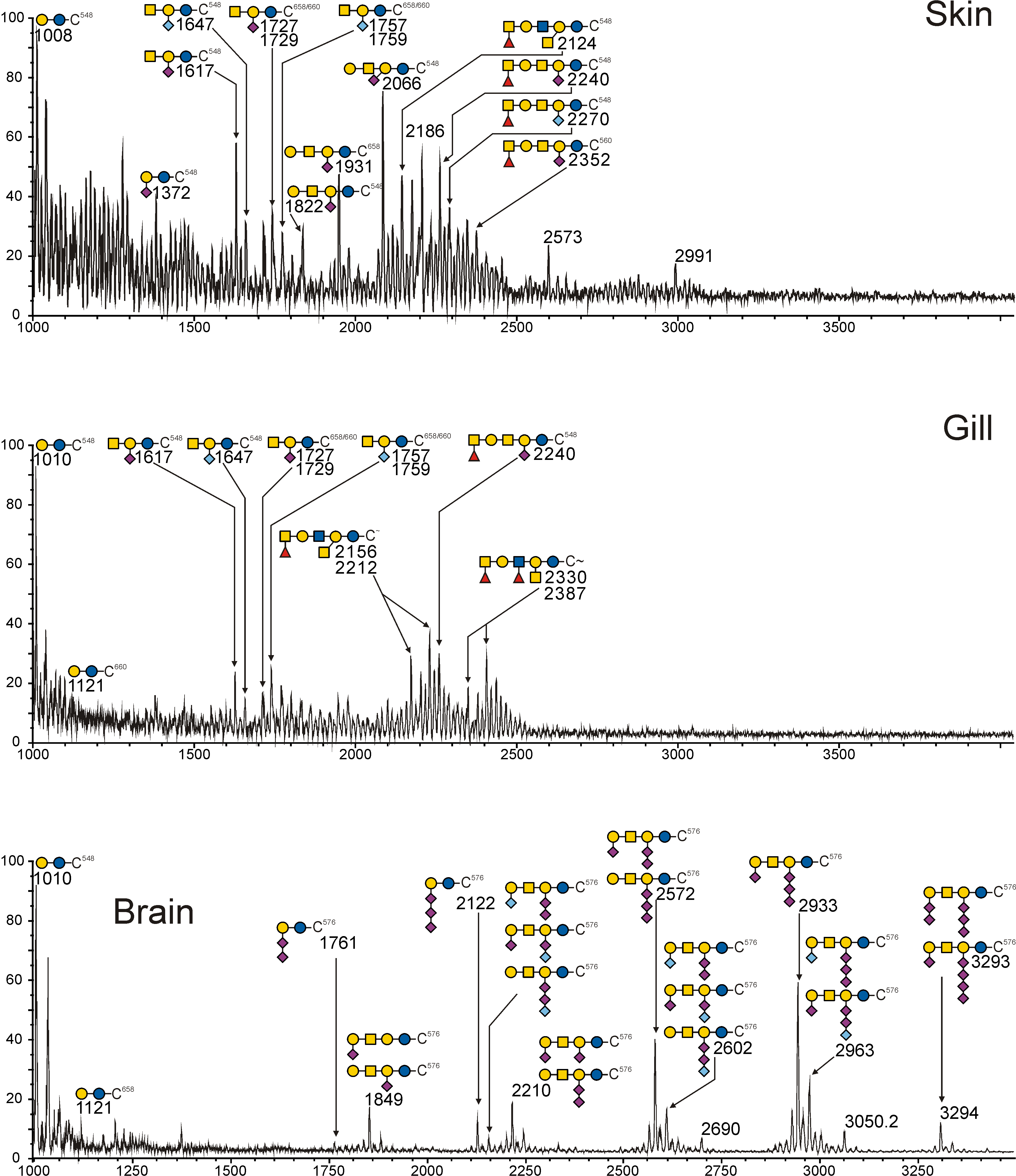


Supplementary Figure 6 (2/3)


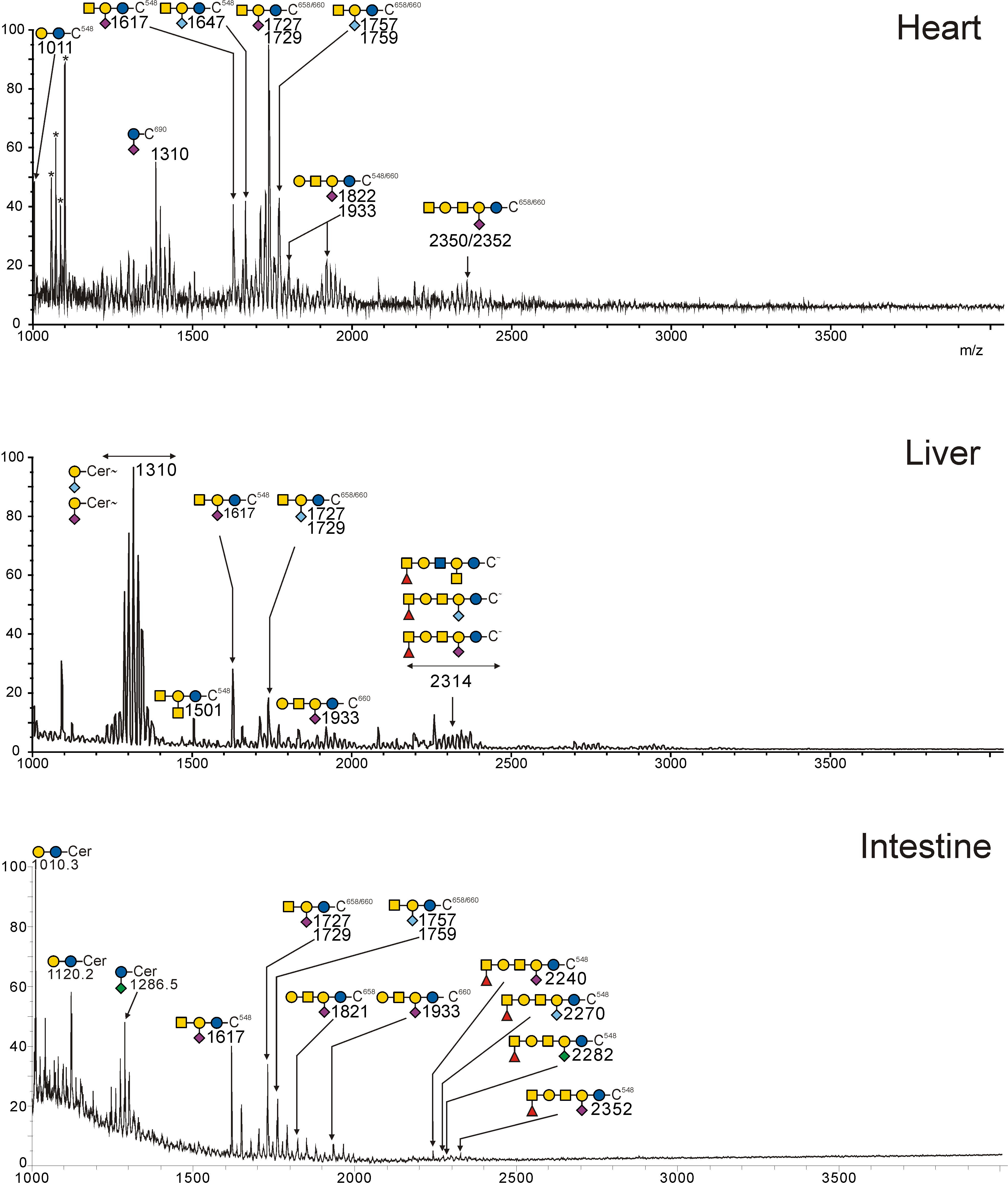


Supplementary Figure 6 (3/3)


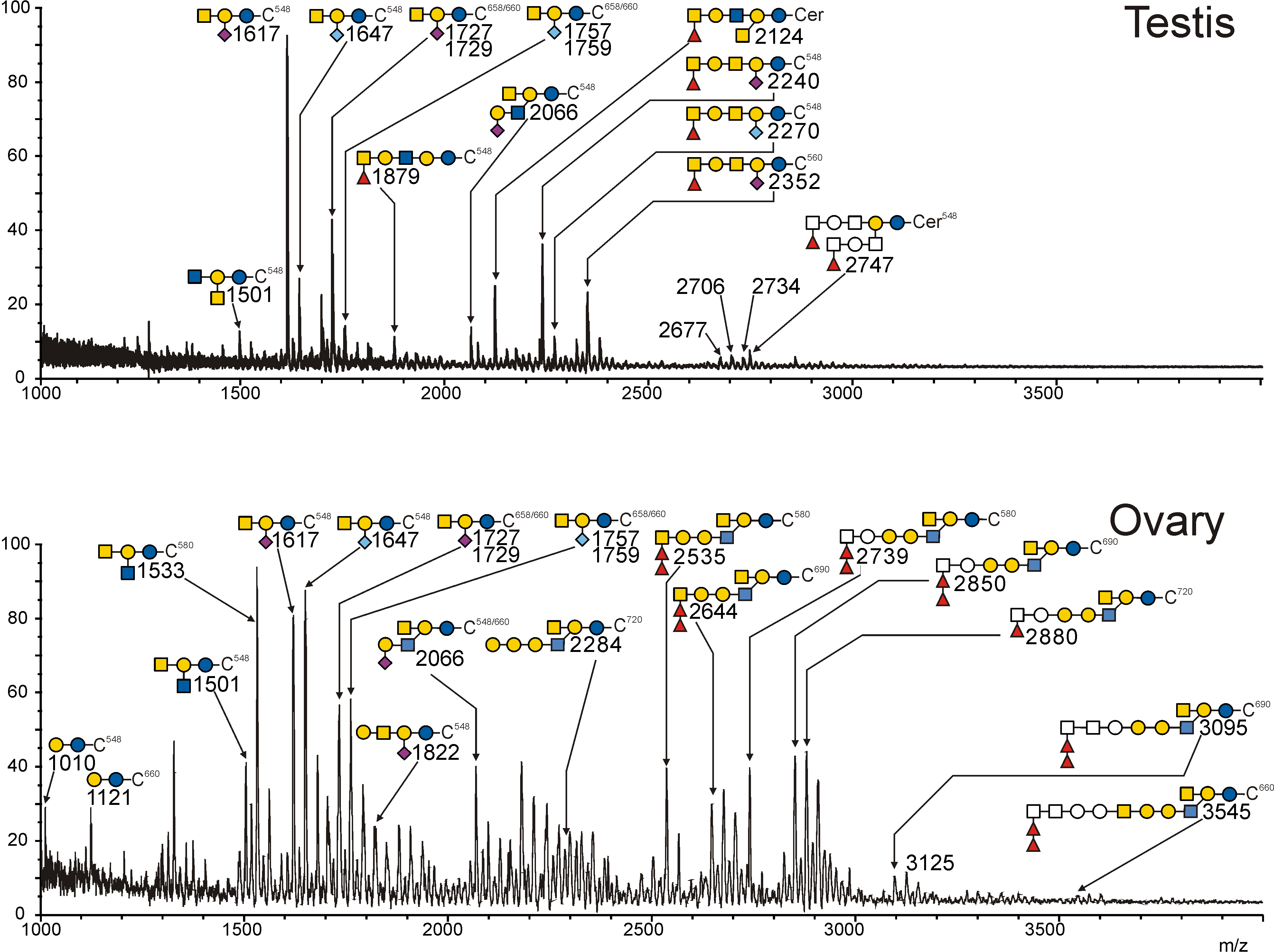


Supplementary Figure 6 - MALDI-TOF-MS spectra of permethylated GSLs isolated from the eight organs. Graphical representation is based on accepted conventions for glycans and monosaccharide nomenclature. White square, HexNAc; white circle, Hex.


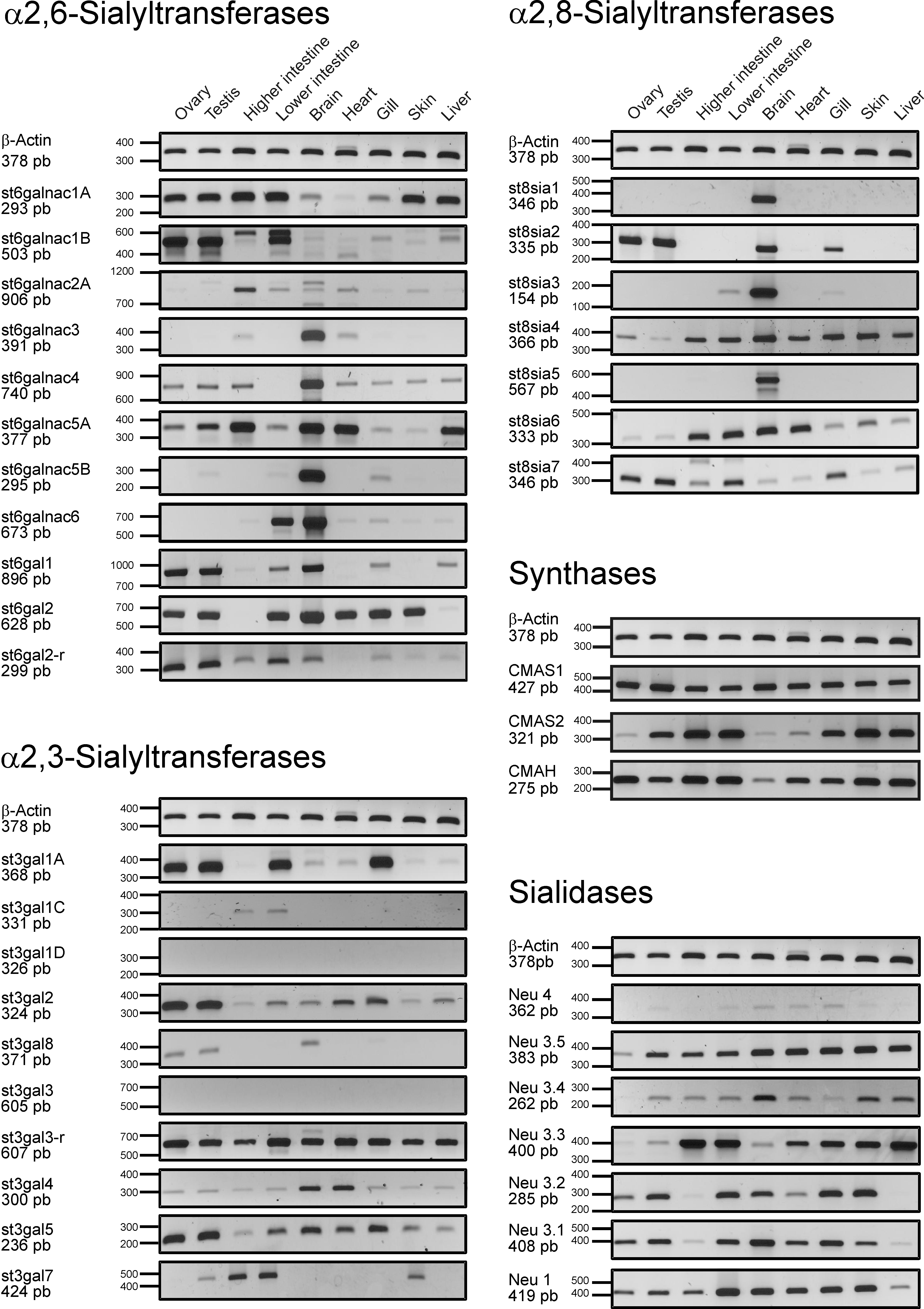


**Supplementary Figure 7** - Expression patterns of sialyltransferases, CMP-sialic acid synthases (CMAS), sialidases (Neu) and CMP-sialic acid hydroxylases (CMAH) genes in various zebrafish tissues using RT-PCR. Relative expression levels of zebrafish genes and β-actin mRNA were evaluated as described in the material and methods. Oligonucleotide primer sequences specific of each zebrafish enzymes (sialidases, CMAH and CMAS) are given in Supplementary Data 5. The zebrafish β-actin (378 bp) was amplified as a control of cDNA synthesis and purity. The uncropped versions of agarose gel electrophoresis are provided in Supplementary Figure 8.

Supplementary Figure 8 (1/3)


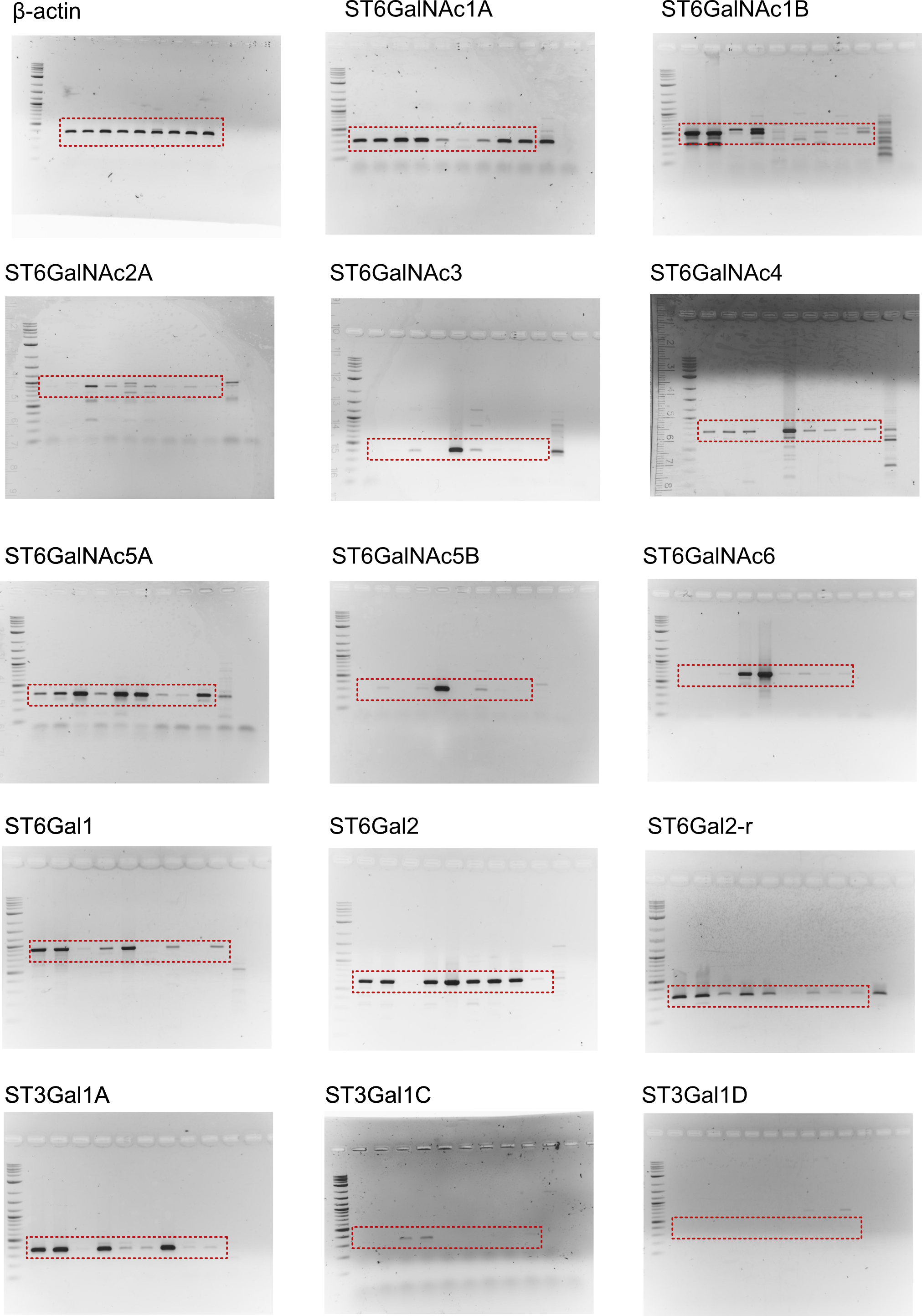


Supplementary Figure 8 (2/3)


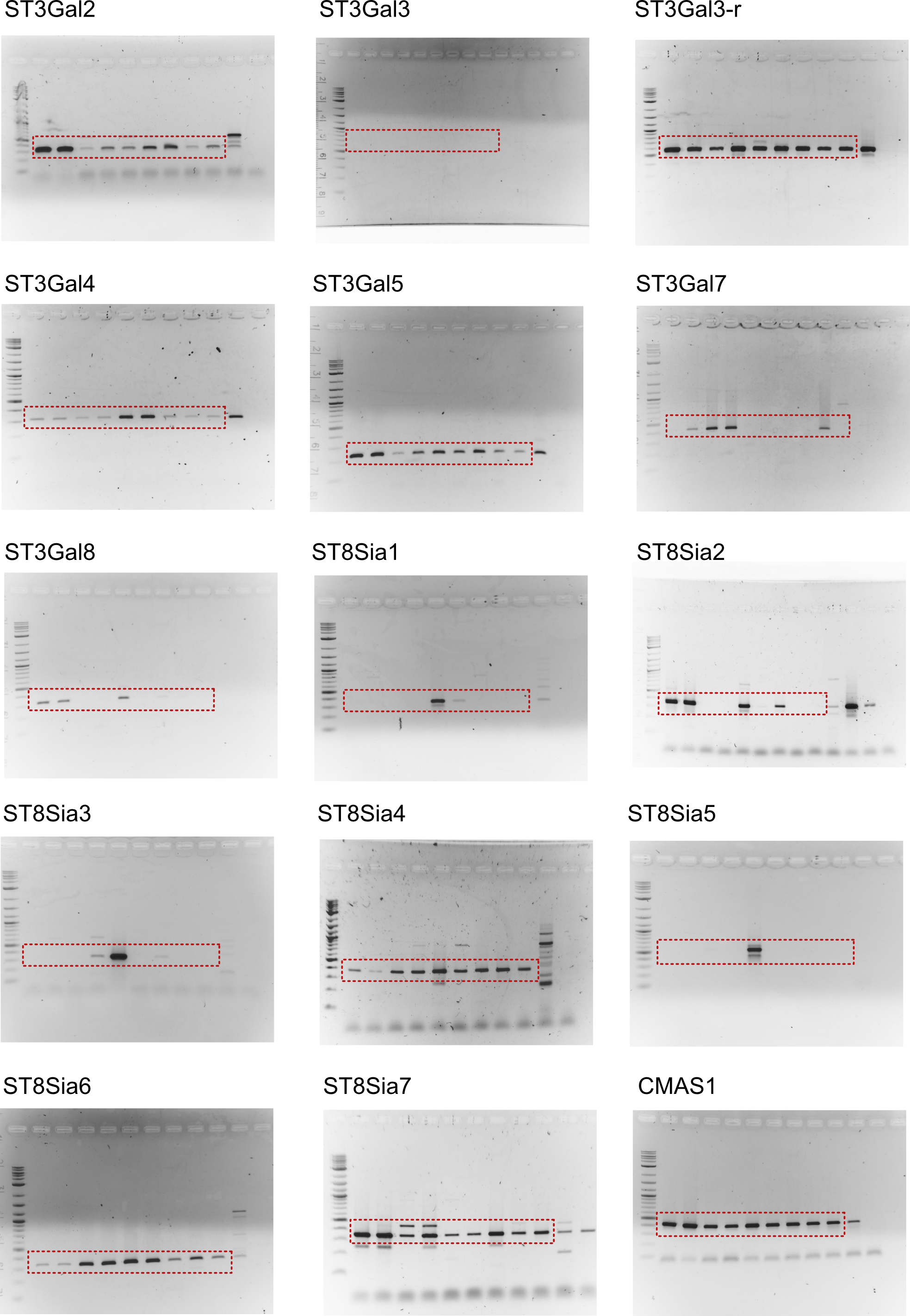


Supplementary Figure 8 (3/3)


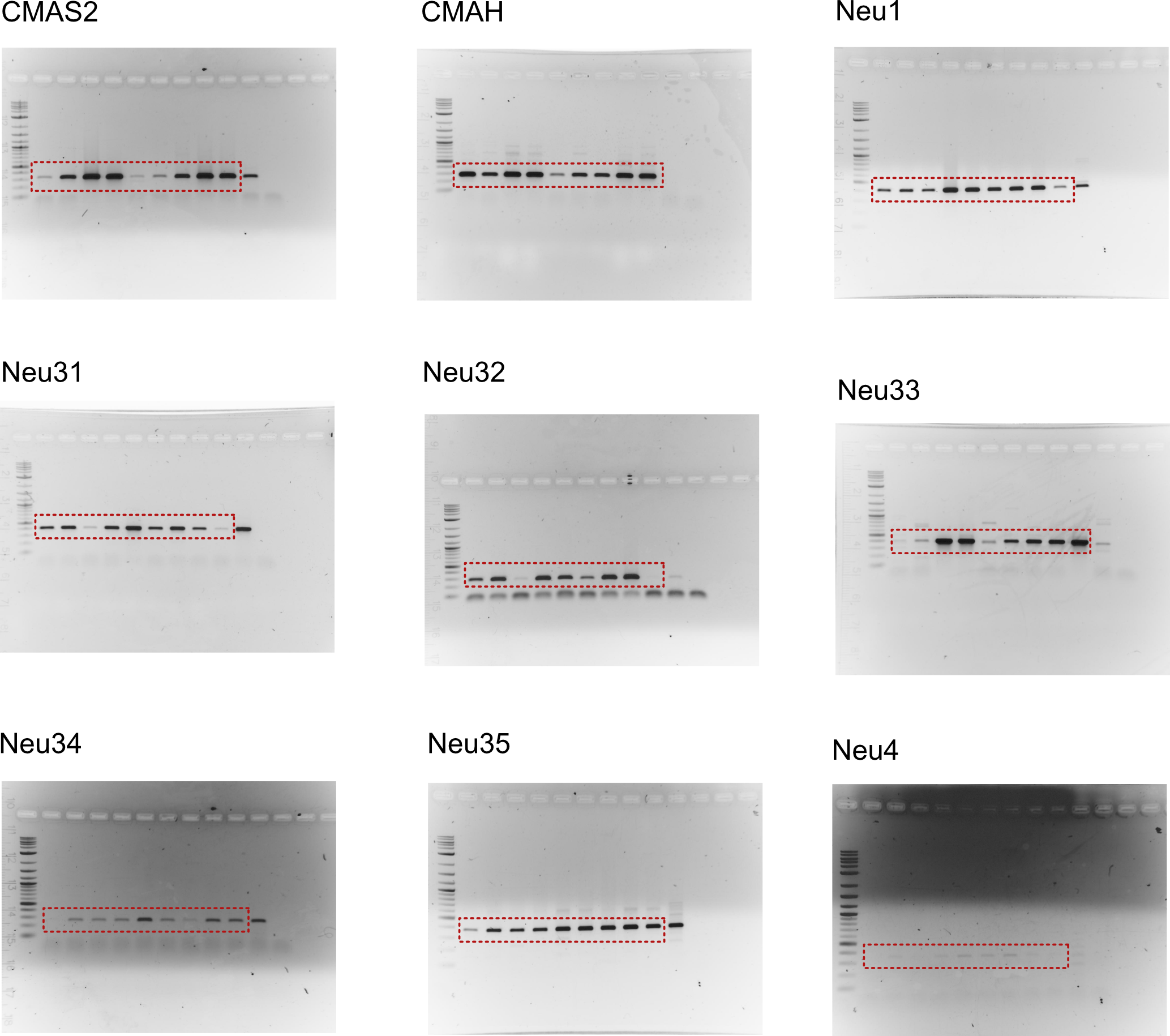


**Supplementary Figure 8** - 2% agarose gel electrophoresis of amplified fragments, visualized by ethidium bromide.


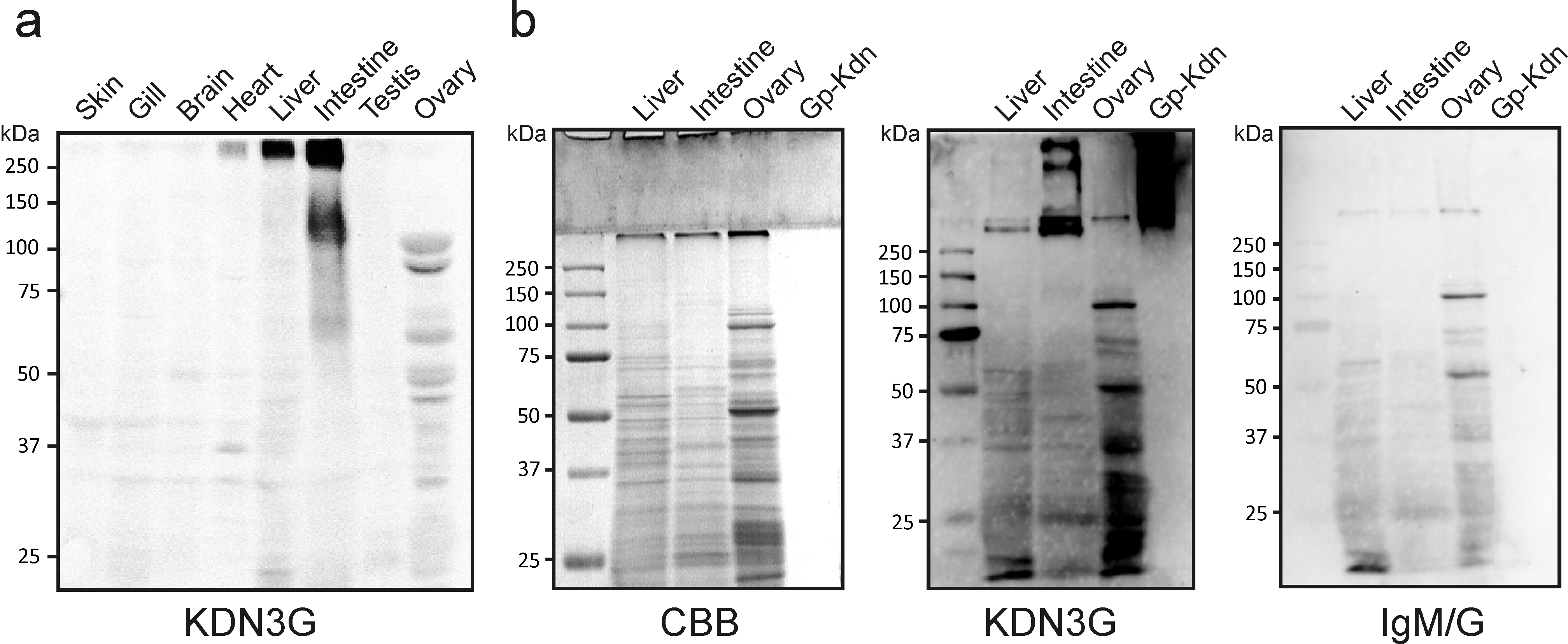


**Supplementary Figure 9** - (a) Monoclonal antibody raised against Kdn(α2,3)Gal epitope (KDN3G) showed a strong response for two high molecular weight broad bands around 125 kDa and above 250 kDa. (b) WB analysis of high molecular weight proteins from SDS-PAGE with concentration gels (CBB, coomassie blue). In addition to the high molecular weight component previously observed, intestine specifically contains KDN3G reactive components that did not reach the separating gel, probably because of their very high molecular weight. Their chromatographic behaviour, as well as the absence of detection by Coomassie Brilliant Blue staining, strongly suggest that these high molecular components are mucin-type highly glycosylated glycoproteins. IgM/G control showed that signals observed in ovary were non-specific.

IgG control

a

IgG control

b


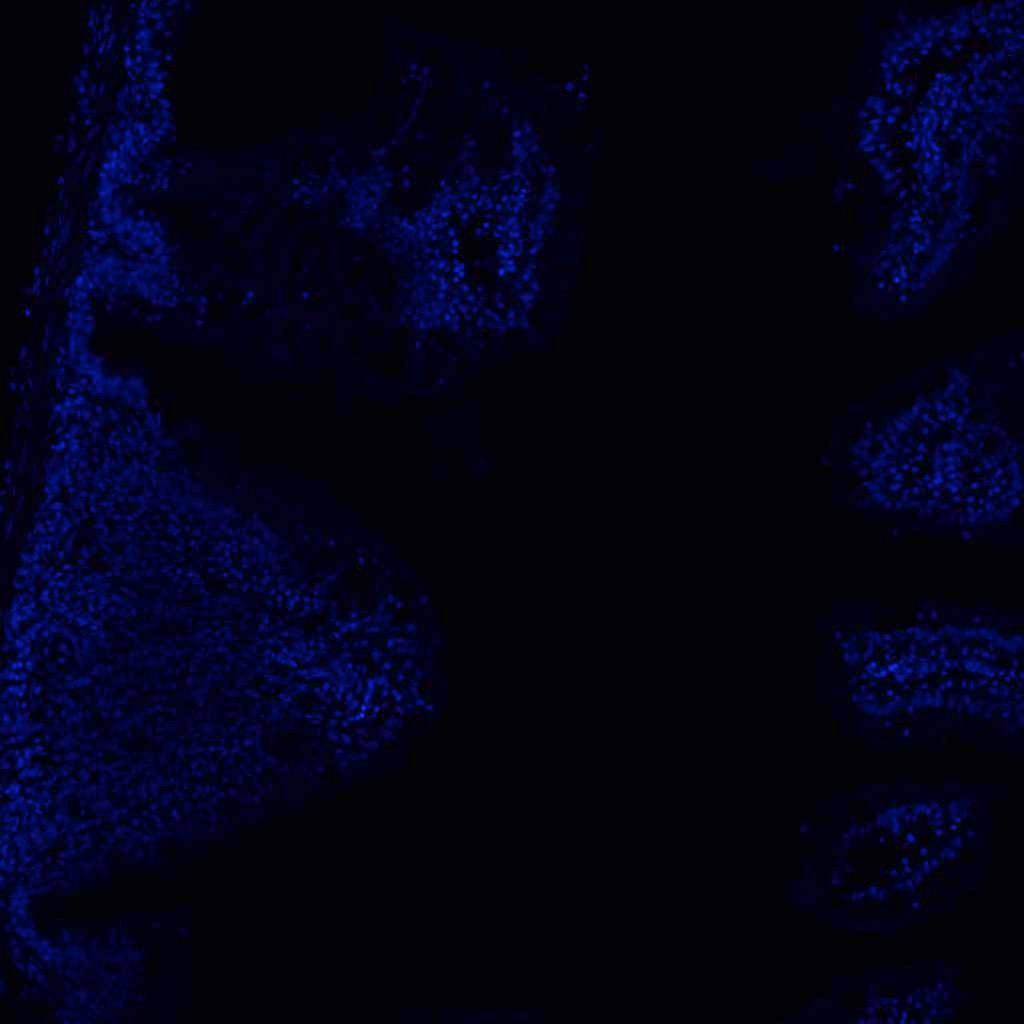

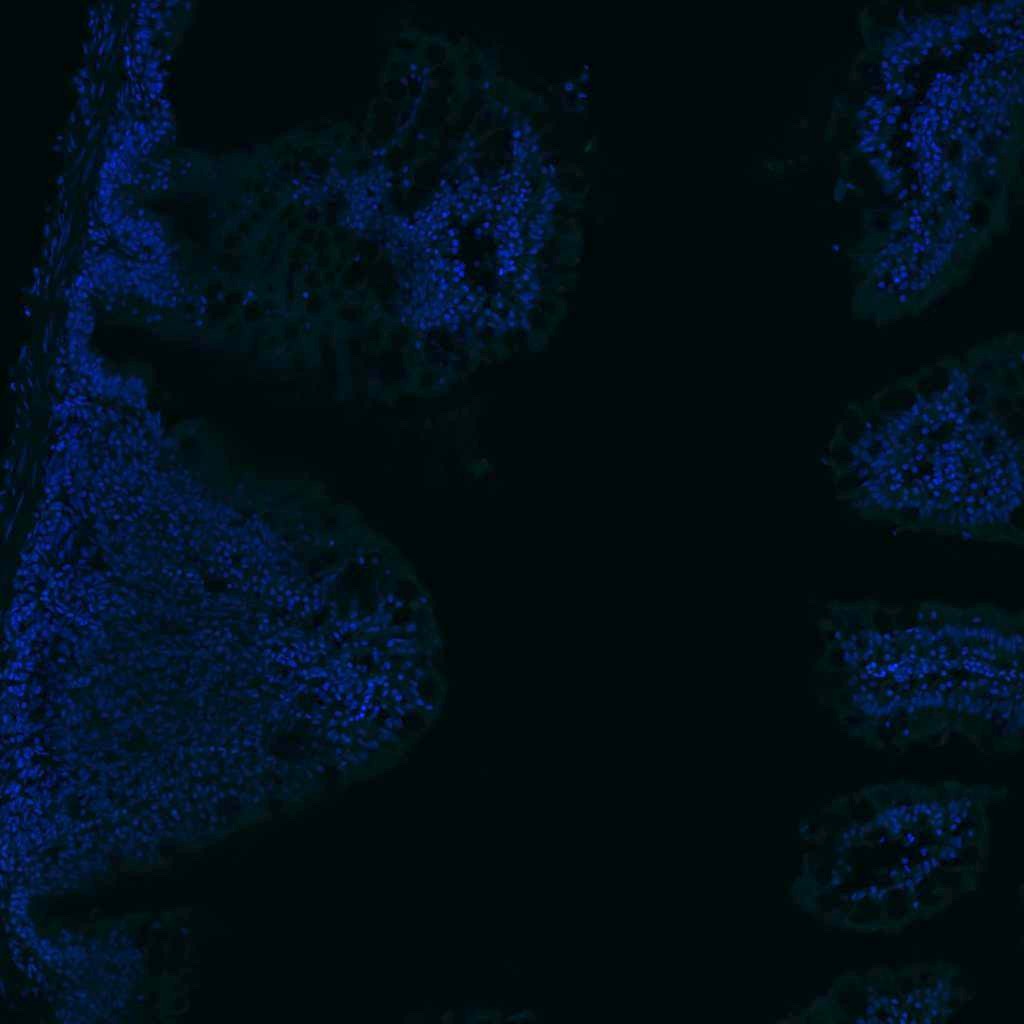

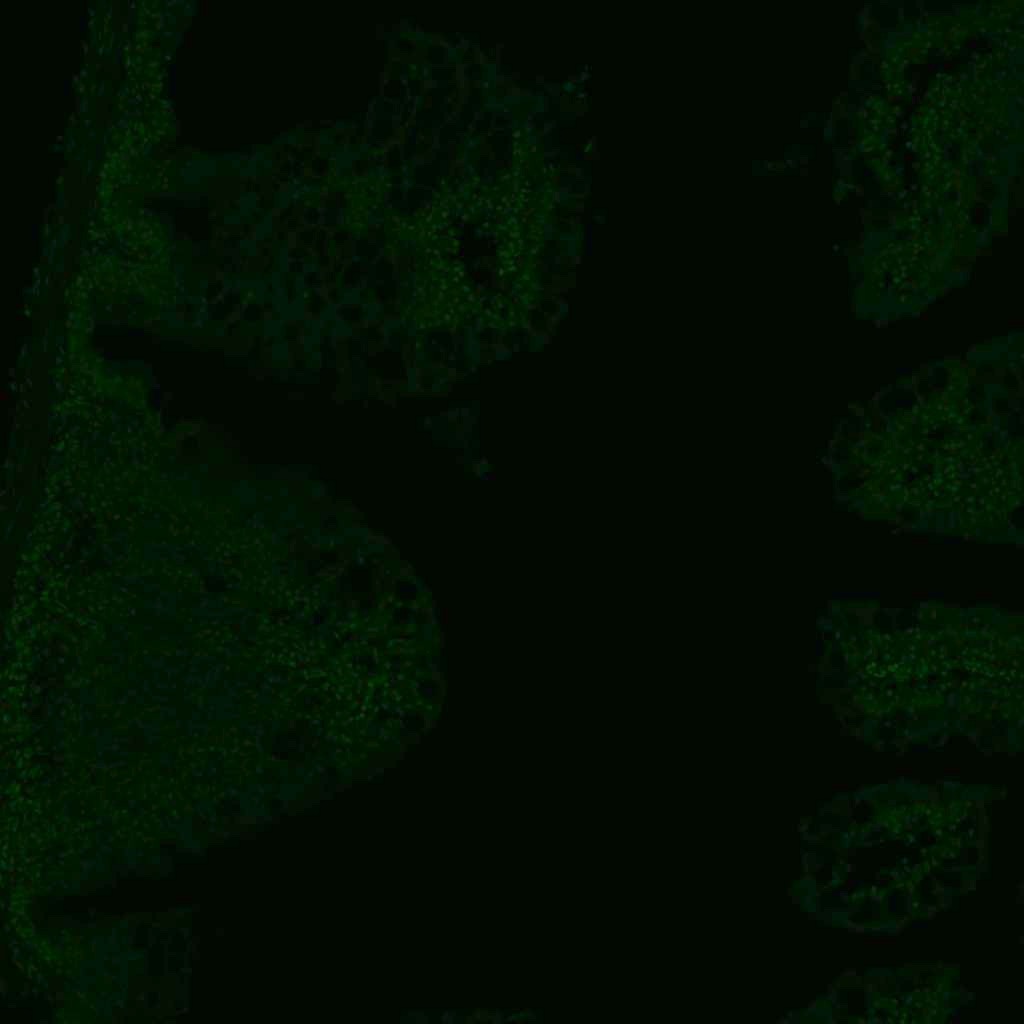

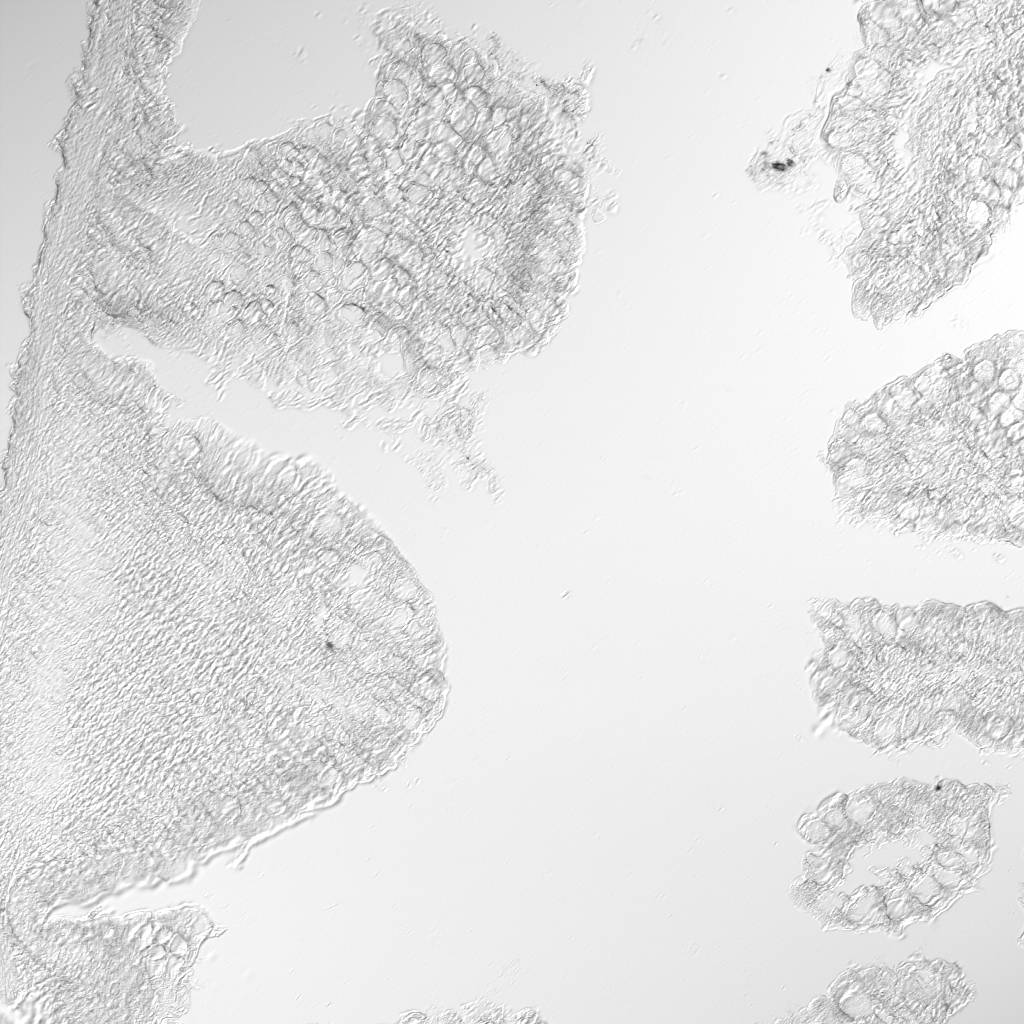


DAPI

DAPI+FITC

FITC

DIC

DAPI

FITC

DIC

DAPI+FITC


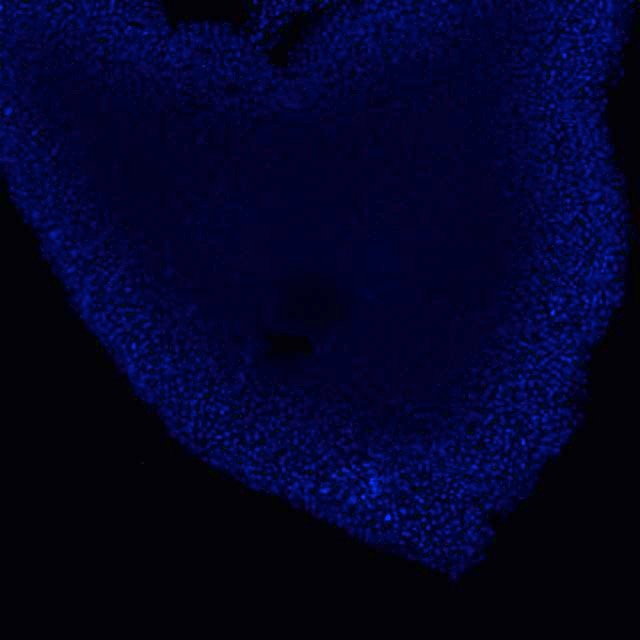

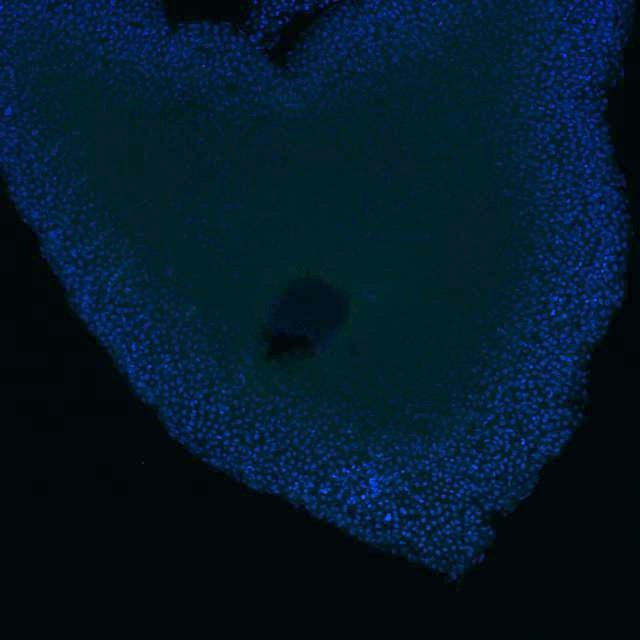

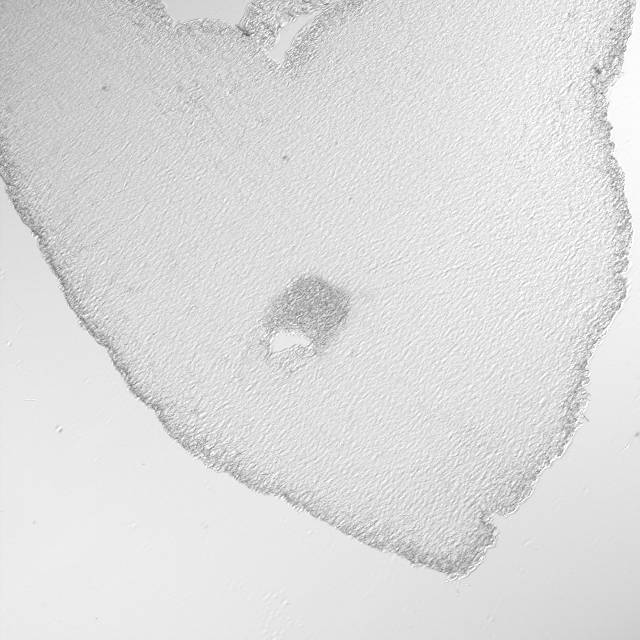

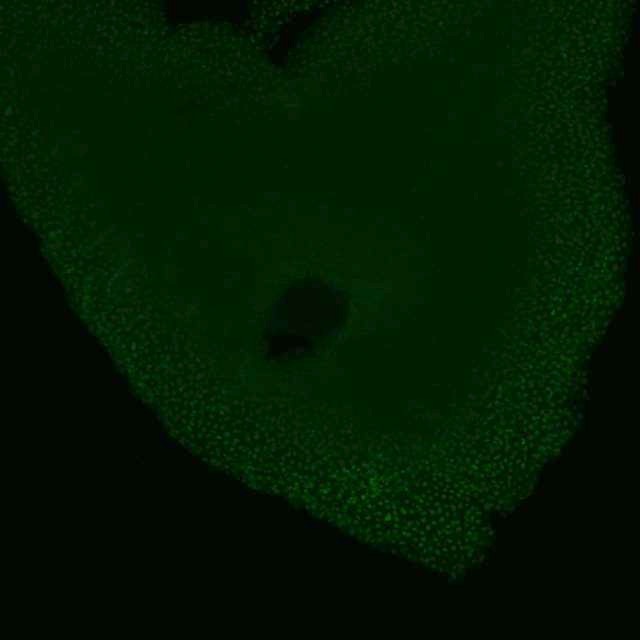


**Supplementary Figure 10** - Control staining of (a) intestine (scale corresponds to 100 μm) and (b) liver (scale corresponds to 50 μm) using IgG only showed background staining.


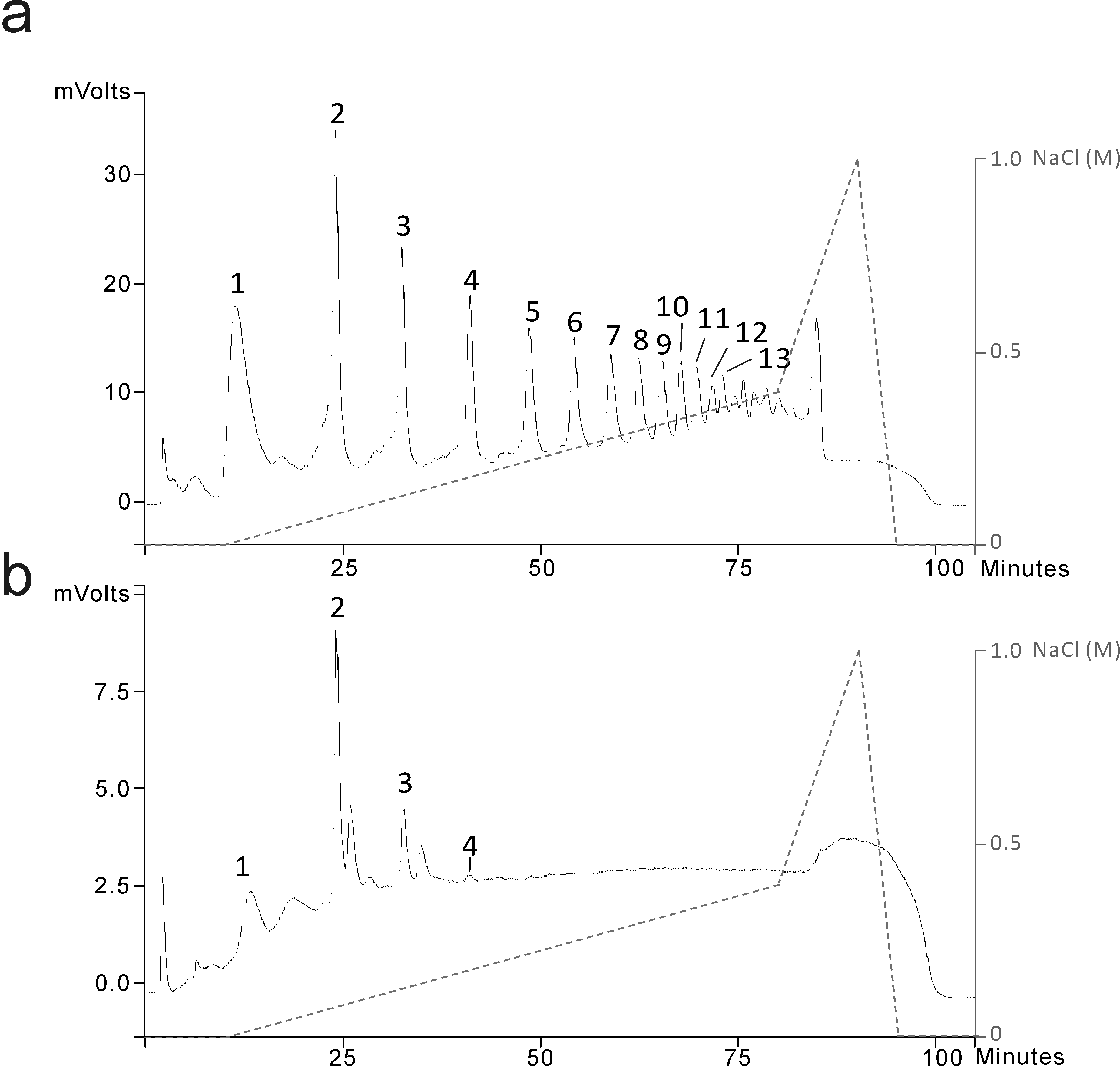


**Supplementary Figure 11** - Profiles of oligosialylated sequences on brain GSLs. OligoSia sequences were released from GSLs isolated from whole brains, tagged with DMB and separated by HPLC-FD on an anion exchange column. OligoSia profiles from (a) polysialic colominic acid used as authentic standard and (b) GSLs. Peaks are labelled according to the DP values as established by comparison with colominic acid. The multiple peaks observed for DP2 and DP3 probably originate from different NeuAc/NeuGc content, as previously shown in embryonic glycoproteins^8^.


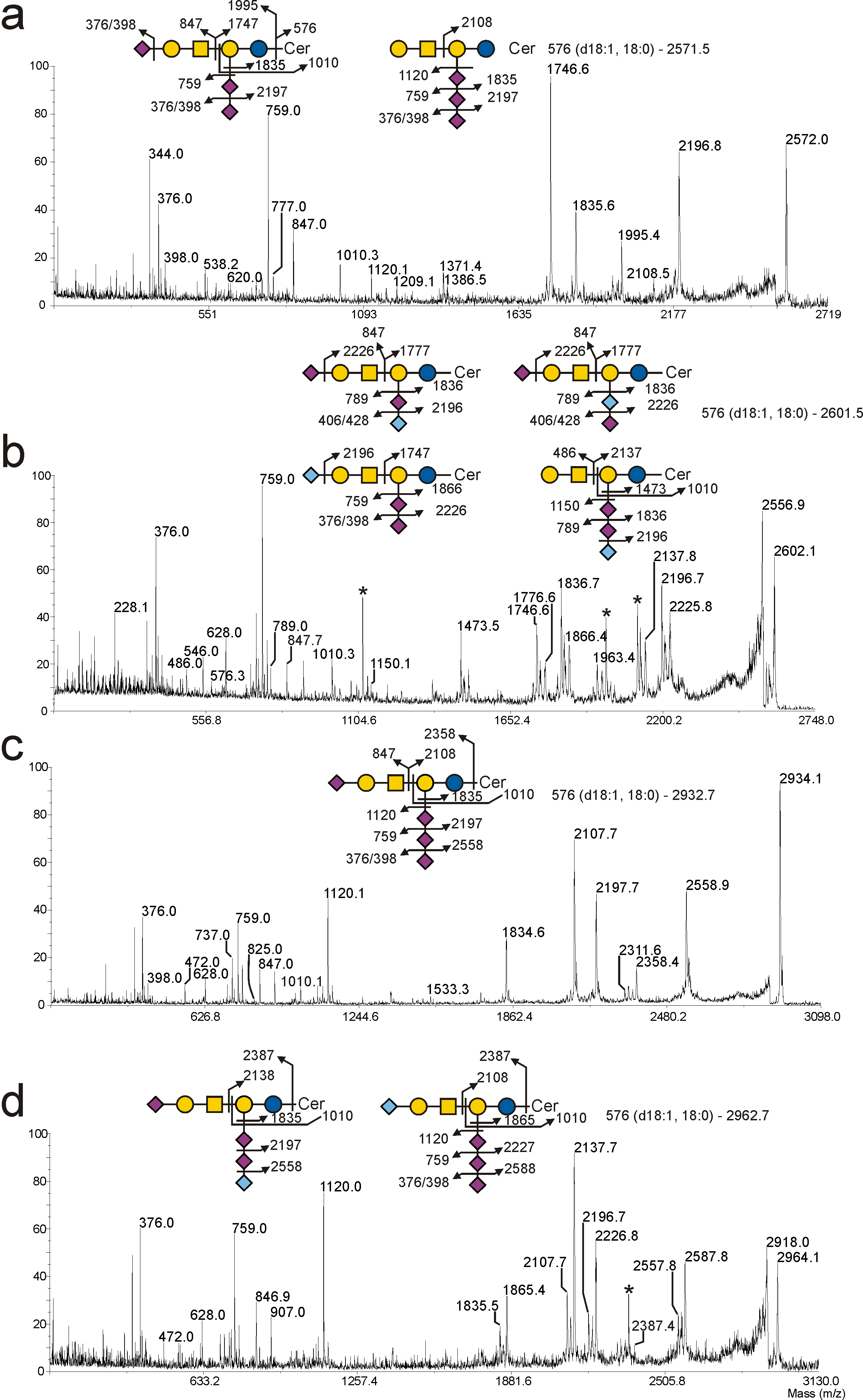


**Supplementary Figure 12** - MALDI-TOF/TOF MS/MS spectra of permethylated GSLs from ganglio-series GL96-97 (a), GL98-99-100-101 (b), GL102 (c) and GL103-104 (d)

Supplementary Figure 13 (1/2)


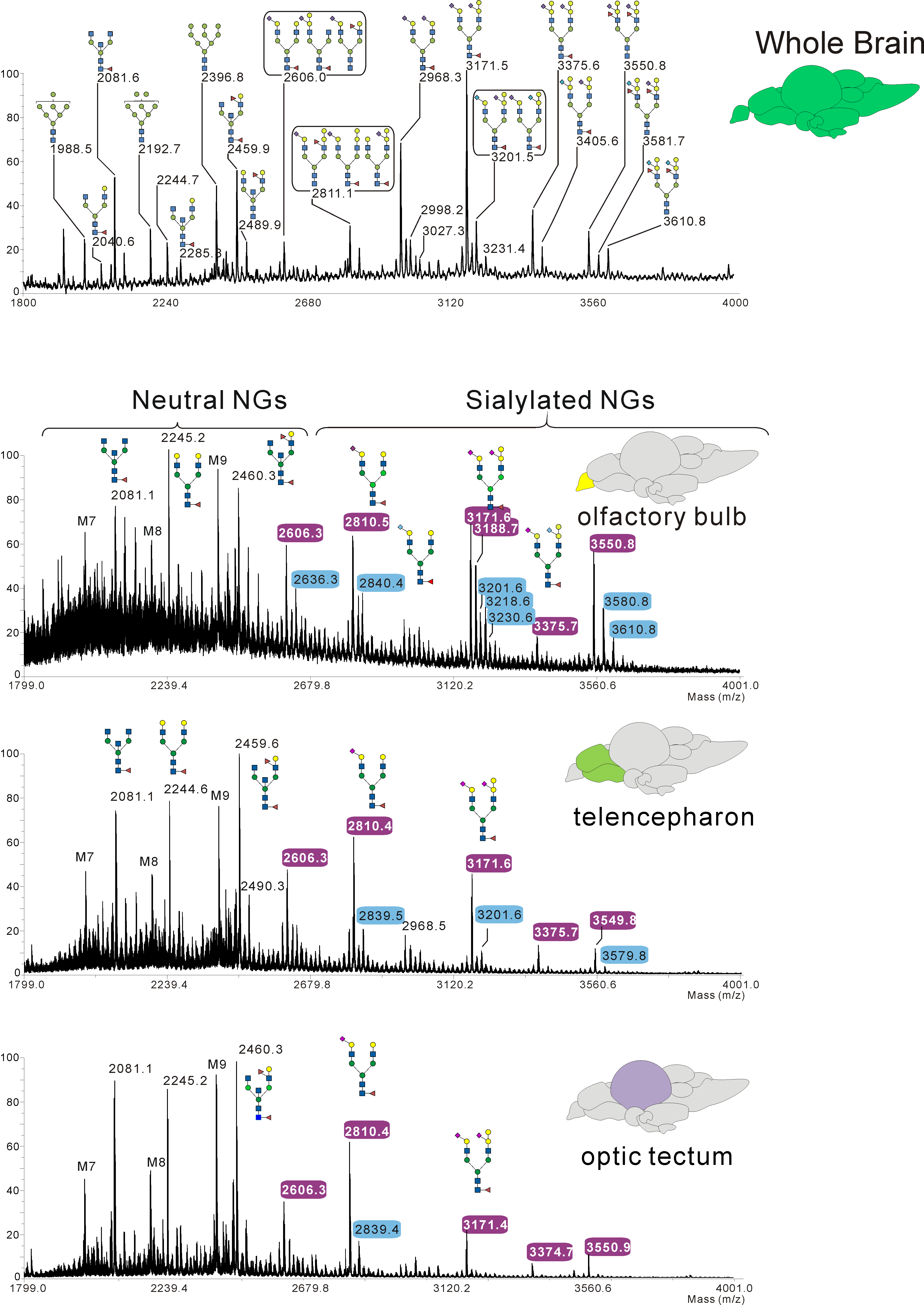


Supplementary Figure 13 (2/2)


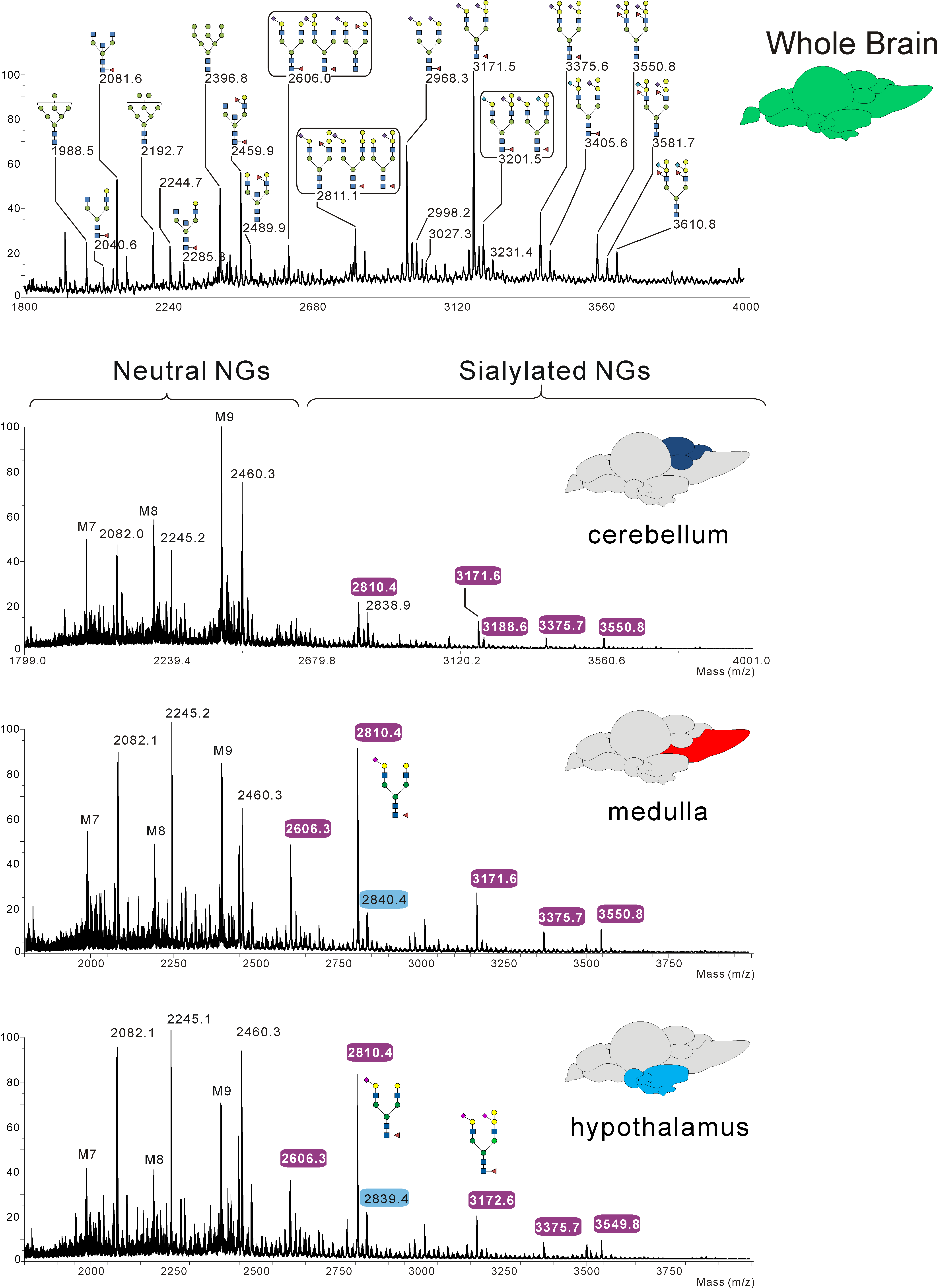


**Supplementary Figure 13** - MALDI-TOF MS spectra of permethylated NGs isolated from whole brain (top) and different regions of brain. Considering the small quantities of starting material, an alternative extraction protocol based on Triton X-100 was used, as described in Methods section of the manuscript. Values highlighted in purple exclusively contain Neu5Ac, those highlighted in light blue contain Neu5Gc. Olfactory bulb is the only region that show intense Neu5Gc associated signals. Analyses were done on three independent samples with similar results.


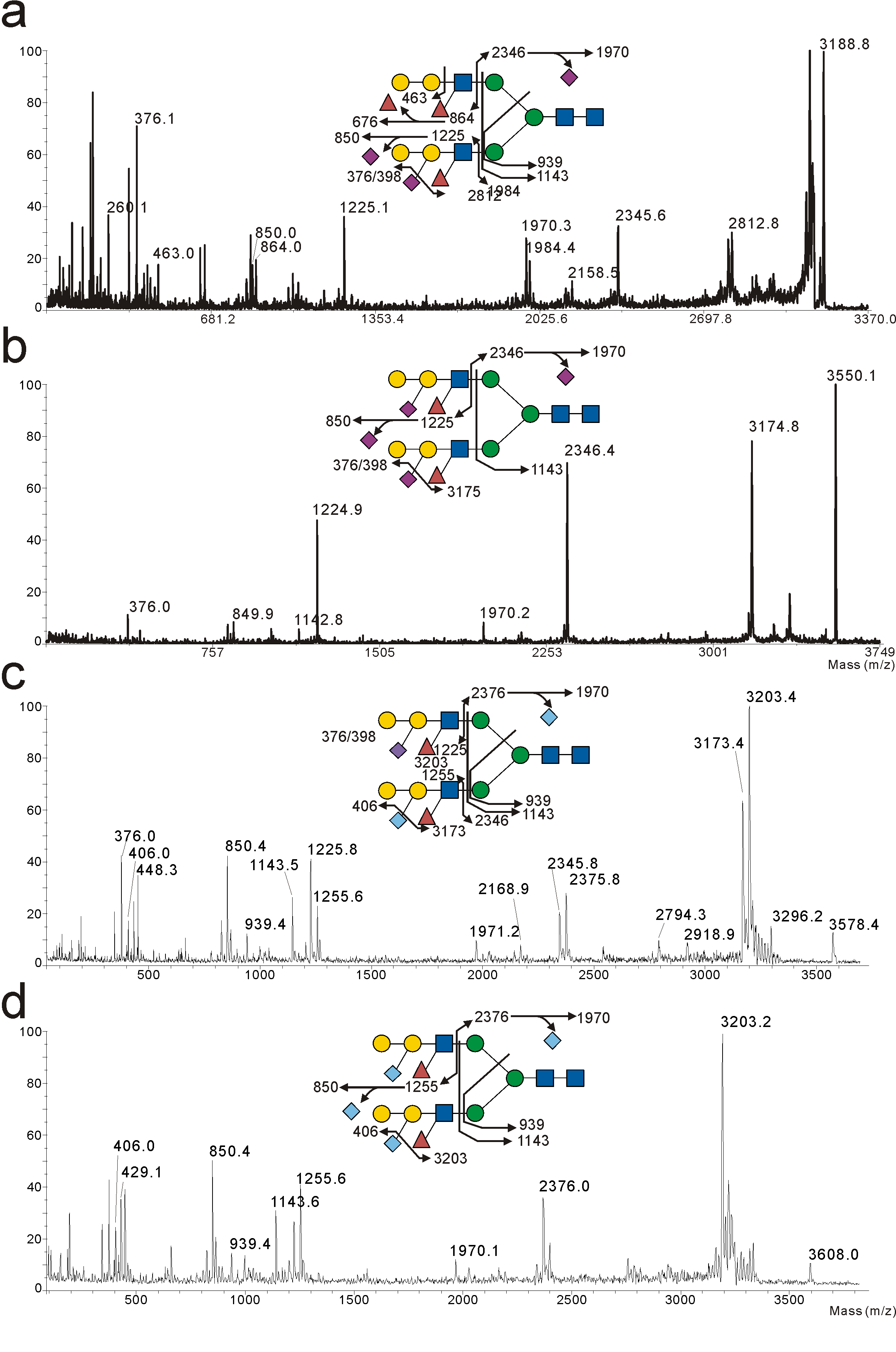


**Supplementary Figure 14** - MALDI-TOF/TOF MS/MS spectra of permethylated zebrafish epitope Gal(β1,4)(NeuAc/Gc(α2,3)]Gal(β1,4)[Fuc(α1,3)]GlcNAc containing NGs NG77 (a), NG89 (b), NG90 (c) and NG91 (d).


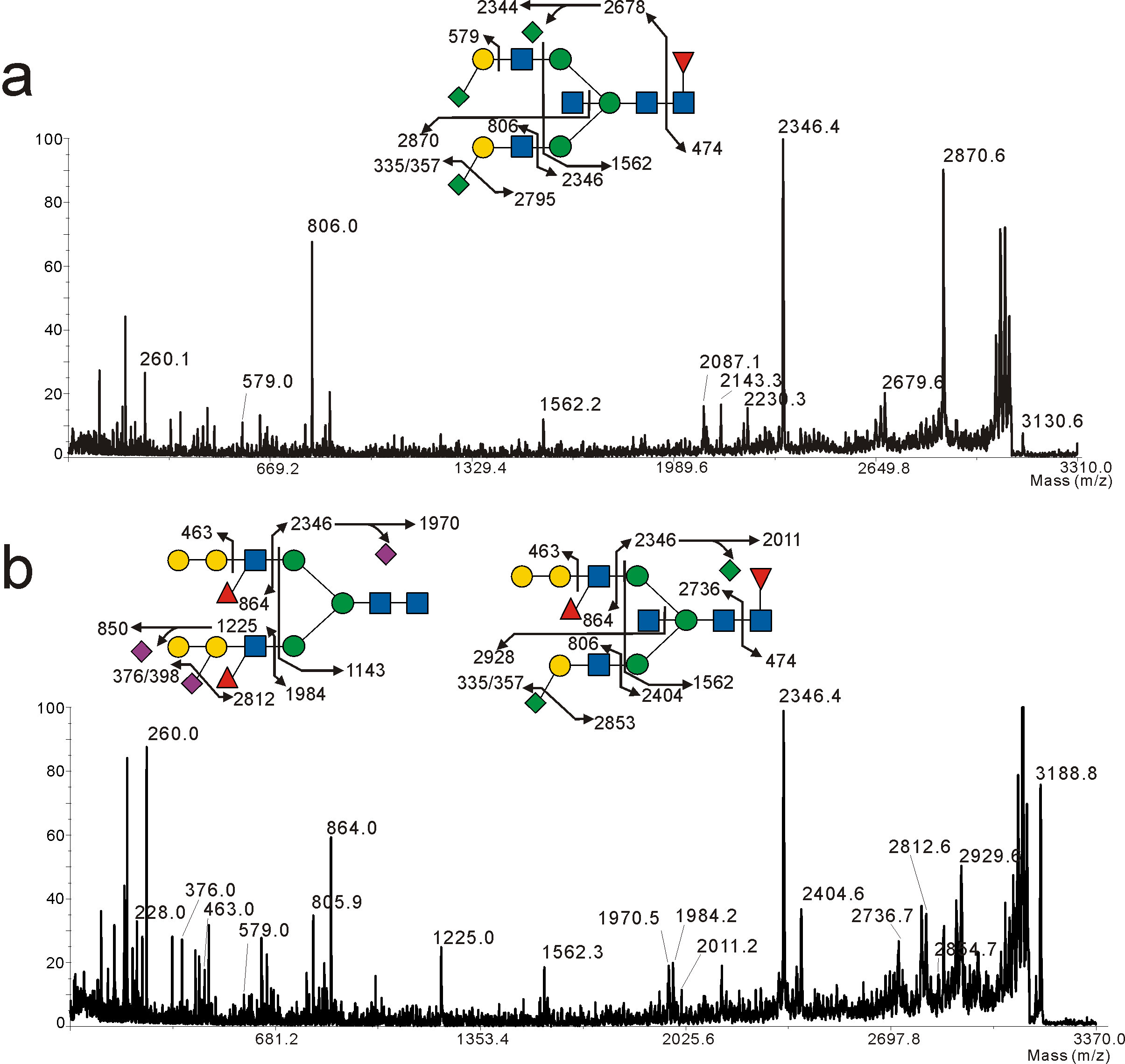


**Supplementary Figure 15** - MALDI-TOF/TOF MS/MS spectra of permethylated Kdn containing NGs NG74 (a) and NG78 (b).


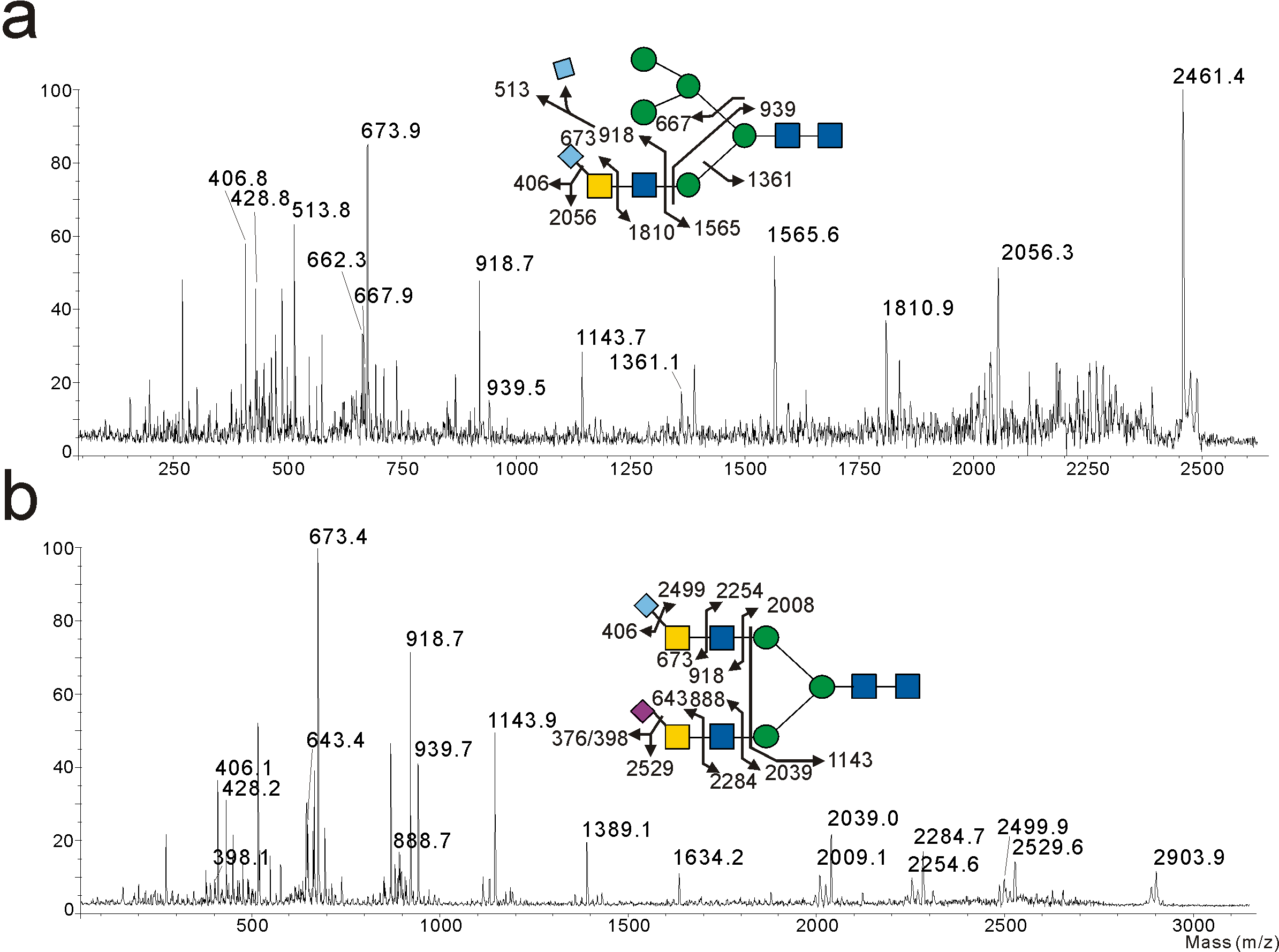


**Supplementary Figure 16** - MALDI-TOF/TOF MS/MS spectra of permethylated LacdiNAc-containing NGs NG24 (a) and NG58 (b).


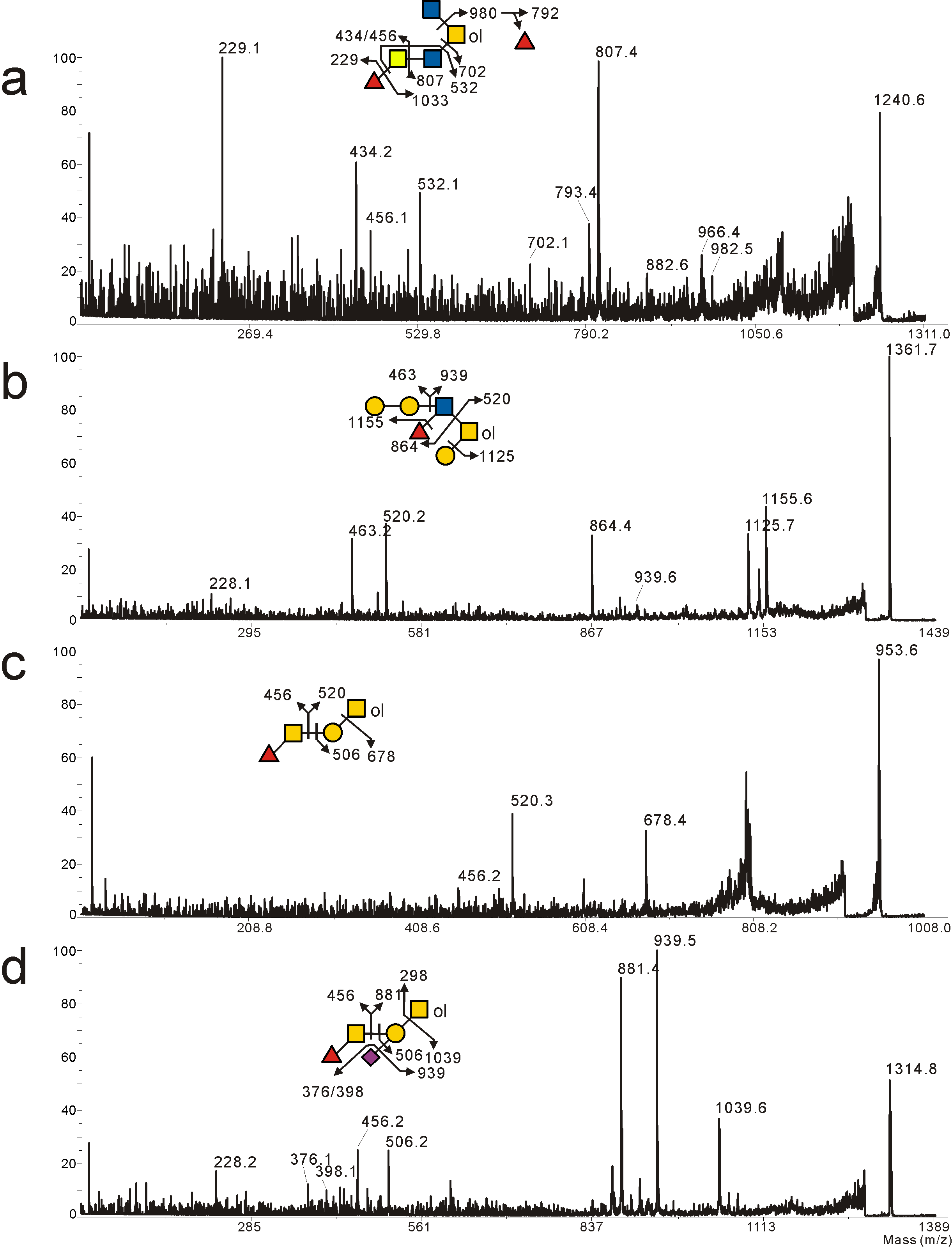


**Supplementary Figure 17** - MALDI-TOF/TOF MS/MS spectra of permethylated OGs OG14 (a), OG23 (b), OG7 (c) and OG18(d)


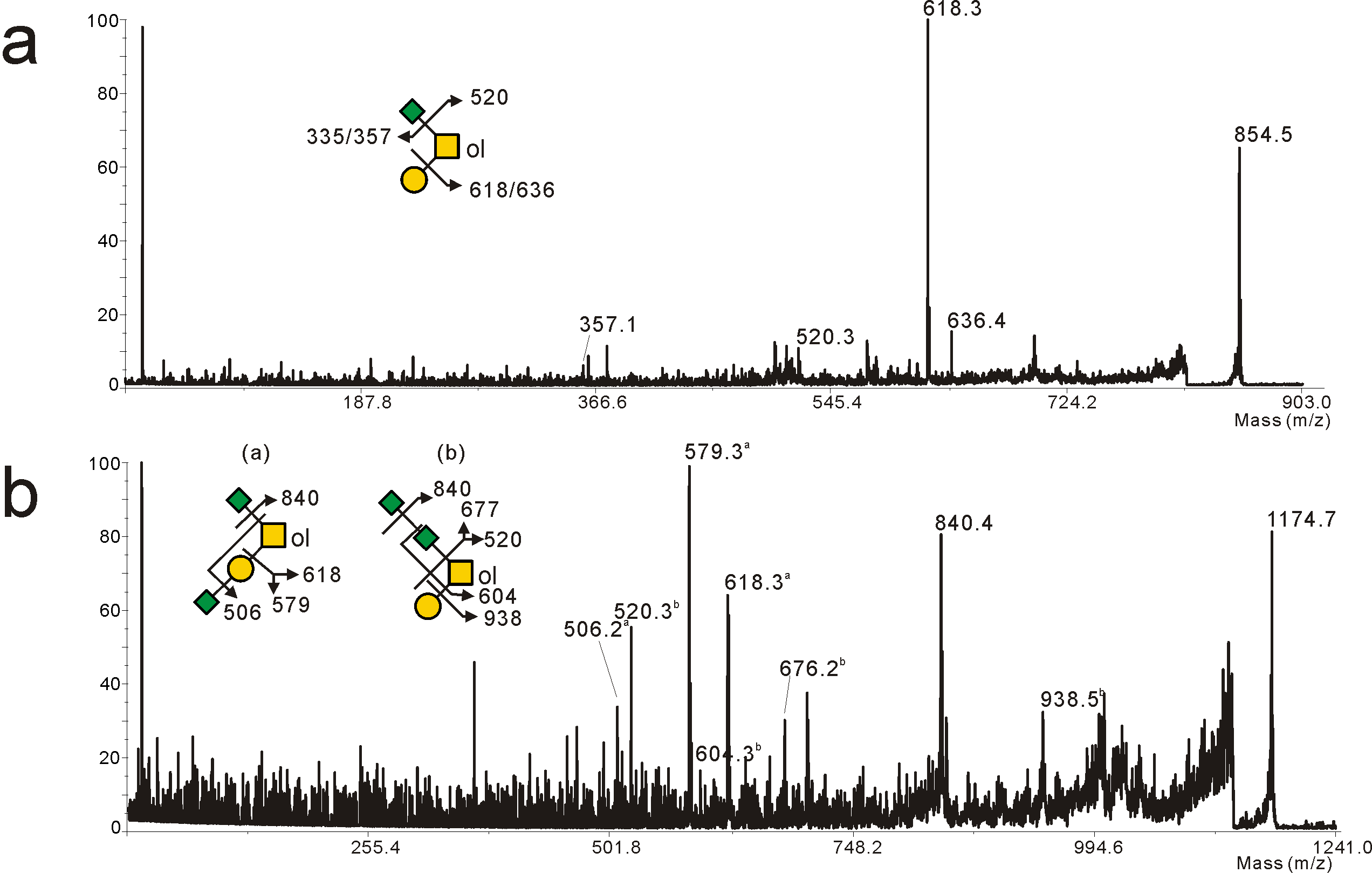


**Supplementary Figure 18** - MALDI-TOF/TOF MS/MS spectra of permethylated Kdn-substituted OGs OG1 (a) and OG10 & OG11 (b)


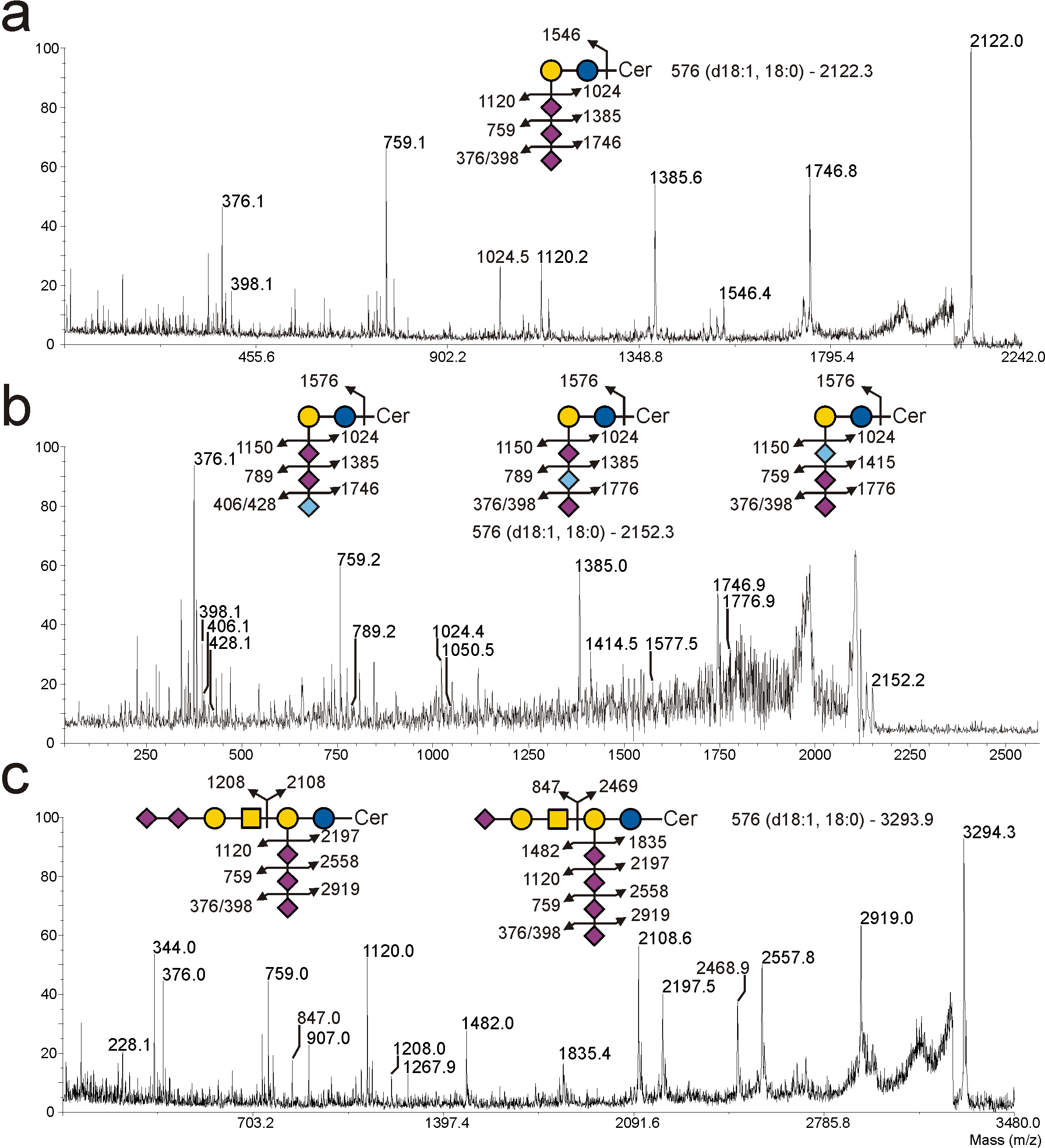


**Supplementary Figure 19** - MALDI-TOF/TOF MS/MS spectra of permethylated GSLs from hemato-series GL63 (a) and GL64-65-66 (b) and from ganglio-series GL105-106 (c)


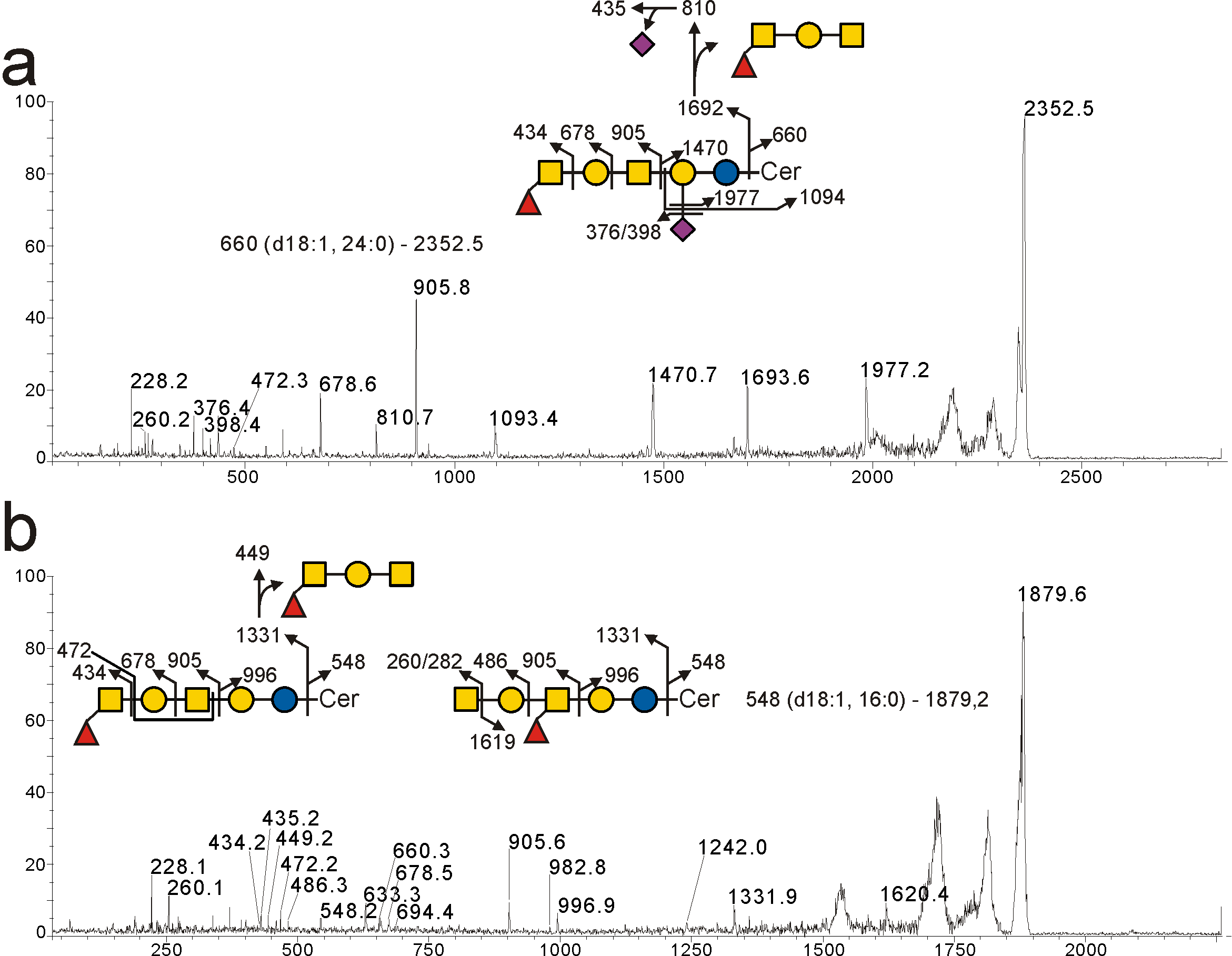


**Supplementary Figure 20** - MALDI-TOF/TOF MS/MS spectra of permethylated GSLs from the extended ganglio-series GL118 (a) and GL107-108 (b)


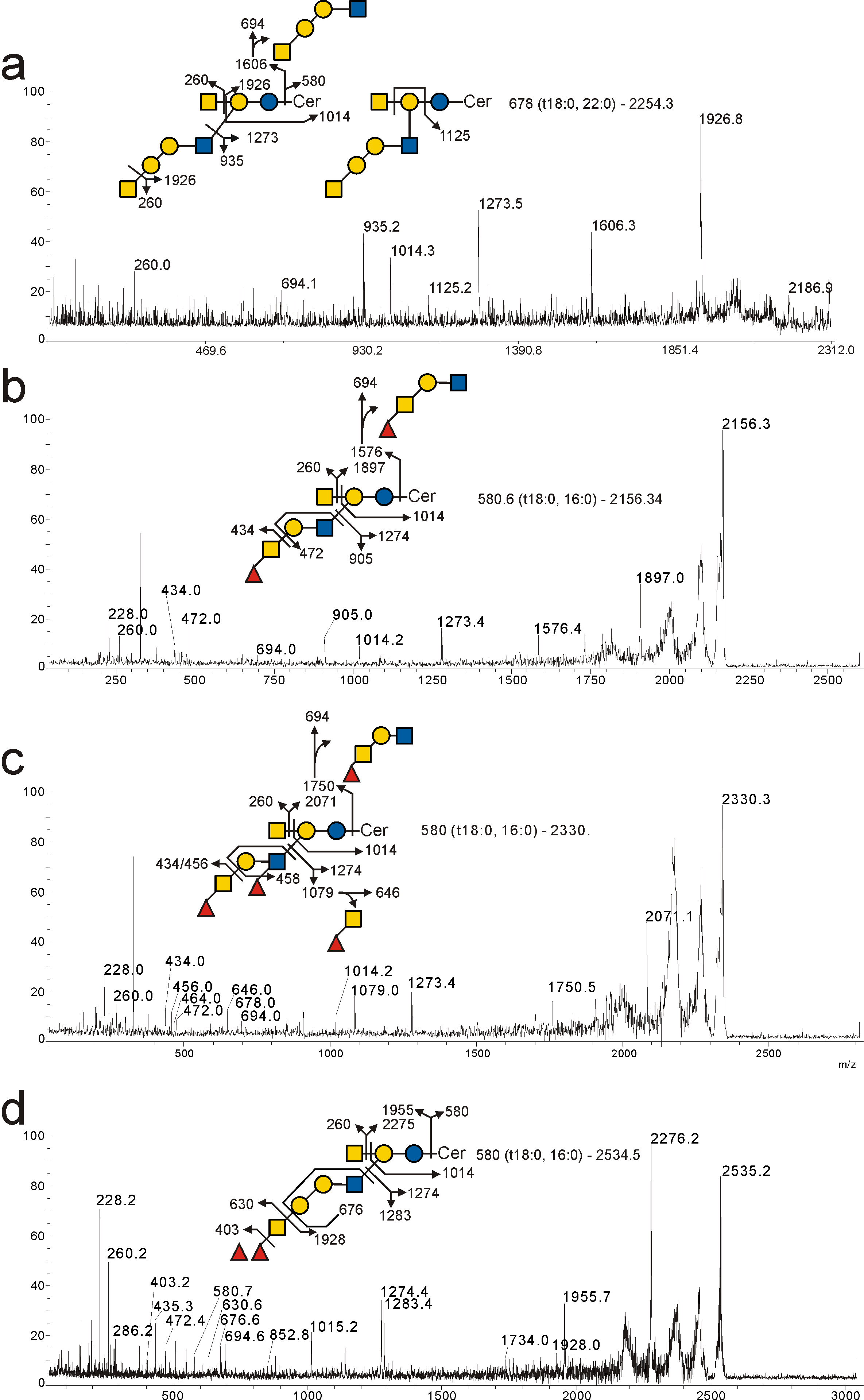


**Supplementary Figure 21** - MALDI-TOF/TOF MS/MS spectra of permethylated GSLs from the lacto-ganglio-series GL149 (a), GL131 (b), GL147 (c) and GL150 (d)

**Supplementary References**

1. Yu, S.-Y., Wu, S.-W. & Khoo, K.-H. Distinctive characteristics of MALDI-Q/TOF and TOF/TOF tandem mass spectrometry for sequencing of permethylated complex type N-glycans. *Glycoconj. J.* **23,** 355–369 (2006).

2. Wada, Y. *et al.* Comparison of the methods for profiling glycoprotein glycans--HUPO Human Disease Glycomics/Proteome Initiative multi-institutional study. *Glycobiology* **17,** 411–422 (2007).

3. Wada, Y. *et al.* Comparison of methods for profiling O-glycosylation: Human Proteome Organisation Human Disease Glycomics/Proteome Initiative multi-institutional study of IgA1. *Mol. Cell. Proteomics MCP* **9,** 719–727 (2010).

4. Aoki, K. *et al.* Dynamic Developmental Elaboration of N-Linked Glycan Complexity in the Drosophila melanogaster Embryo. *J. Biol. Chem.* **282,** 9127–9142 (2007).

5. Guérardel, Y., Chang, L.-Y., Maes, E., Huang, C.-J. & Khoo, K.-H. Glycomic survey mapping of zebrafish identifies unique sialylation pattern. *Glycobiology* **16,** 244–257 (2006).

6. Vanbeselaere, J. *et al.* Mapping the expressed glycome and glycosyltransferases of zebrafish liver cells as a relevant model system for glycosylation studies. *J. Proteome Res.* **11,** 2164–2177 (2012).

7. Hanzawa, K., Suzuki, N. & Natsuka, S. Structures and developmental alterations of N-glycans of zebrafish embryos. *Glycobiology* **27,** 228–245 (2017).

8. Chang, L.-Y. *et al.* Developmental regulation of oligosialylation in zebrafish. *Glycoconj. J.* **26,** 247–261 (2009).

9. Guérardel, Y., Chang, L.-Y., Maes, E., Huang, C.-J. & Khoo, K.-H. Glycomic survey mapping of zebrafish identifies unique sialylation pattern. *Glycobiology* **16,** 244–257 (2006).

10. Niimura, Y. & Ishizuka, I. Unique disialosyl gangliosides from salmon kidney: Characterization of V3αFuc, IV3βGalNAc, II3(αNeuAc)2-Gg4Cer and its analogue with 4-O-acetyl-N-acetylneuraminic acid. *Glycoconj. J.* **23,** 489–499 (2006).

11. DeGasperi, R. *et al.* Isolation and characterization of gangliosides with hybrid neolacto-ganglio-type sugar chains. *J. Biol. Chem.* **262,** 17149–17155 (1987).

12. Ostrander, G. K., Levery, S. B., Hakomori, S. & Holmes, E. H. Isolation and characterization of the major acidic glycosphingolipids from the liver of the English sole (Parophrys vetulus). Presence of a novel ganglioside with a Forssman antigen determinant. *J. Biol. Chem.* **263,** 3103–3110 (1988).

13. Ostrander, G. K. *et al.* Isolation and characterization of four major neutral glycosphingolipids from the liver of the English sole (Parophrys vetulus). Presence of a novel branched lacto-ganglio-iso-globo hybrid structure. *J. Biol. Chem.* **263,** 18716–18725 (1988).
