# Supplementary material for: Systems glycomics of adult zebrafish identifies organ-specific sialylation and glycosylation patterns
Source: Nat Commun. 2018 Nov 7;9:4647. doi: 10.1038/s41467-018-06950-3 (PMC6220181; doi:10.1038/s41467-018-06950-3)
Supplement: Supplementary file 3 — Description of Additional Supplementary Files [file 41467_2018_6950_MOESM3_ESM.pdf]

Description of Additional Supplementary Files

File Name: Supplementary Data 1
Description: The inclusion criteria of individual molecules in an organ were (1) observation of corresponding MS signal in three independent experiments and (2) confirmation of individual compounds by MS/MS analysis of permethylated glycans. The structural analysis of Nglycans was mostly based on the CID MS/MS fragmentation patterns of permethylated N-glycans in positive mode, in accordance with knowledge of embryonic N-glycans1–5. Graphical representation is based on accepted conventions for N-glycans and monosaccharide nomenclature as follows: yellow circle, Gal; yellow square, GalNAc; blue circle, Glc; blue square, GlcNAc; green circle, Man; red triangle, Fuc; purple diamond, Neu5Ac; light blue diamond, Neu5Gc; green diamond, Kdn.6,7 Core Fucose is de facto positioned on reducing GlcNAc residue on C6 carbon as for all vertebrate Nglycans. The interglycosidic bonds between monosaccharides of antennae are represented using the conventional positions as in I, C2 position;／, C3 position; 一, C4 position;＼, C6 position. The respective positions of C3 and C6 branches on internal Man residue of N-glycan core are just indicative.

File Name: Supplementary Data 2
Description: The inclusion criteria of individual molecules in an organ were (1) observation of corresponding MS signal in three independent experiments and (2) confirmation of individual compounds by MS/MS analysis of permethylated glycans. The structural analysis of Oglycans was mostly based on the CID MS/MS fragmentation patterns of permethylated O-glycans in positive mode, in accordance with knowledge of embryonic O-glycans1–3. Graphical representation is based on accepted conventions for O-glycans and monosaccharide nomenclature.4,5 The interglycosidic bonds between monosaccharides are represented using the conventional positions as I, C2 position; ／, C3 position; 一, C4 position;＼, C6 position, except for (-2,8) linkages between sialic acids which do not follow any convention.

File Name: Supplementary Data 3
Description: The inclusion criteria of individual molecules in an organ were (1) observation of corresponding MS signal in three independent experiments and (2) confirmation of individual compounds by MS/MS analysis of permethylated glycans. The sequence of carbohydrate moieties and the nature of ceramides were established byCID MS/MS fragmentation of permethylated GSLs in positive mode. Values in "mass" column correspond to the diagnostic Z fragmentation ions for the methylated ceramide moiety. Most probable combinations of sphingoid bases and fatty acids based on composition and Z fragment ions are provided in the "SB, FA" column. Graphical representation is based on accepted conventions for GSLs and monosaccharide nomenclature.1,2 The interglycosidic bonds between monosaccharides are represented using the conventional positions as I, C2 position;／, C3 position; 一, C4 position;＼, C6 position, except for (α2,8) linkages between sialic acids which

do not follow any convention. Whenever linkages could not be deduced from specific CID MS/MS fragmentation pattern and literature, they are shown as dotted lines.

File Name: Supplementary Data 4
Description: Distribution of carbohydrate moieties of GSLs among the eight screened adult organs, compiled from identification structural analysis of intact GSLs (Supplementary Data 3).

File Name: Supplementary Data 5
Description: Oligonucleotide sequences and expected amplicon sizes used for PCR detection of actin, sialyltransferases, sialidases, CMP-sialic acid synthases (CMAS) and CMP-sialic acid hydroxylases (CMAH). Accession numbers in GenBank are indicated. data are presented in Figure 7 and Supplementary Figure 7.
