# Supplementary material for: Systems glycomics of adult zebrafish identifies organ-specific sialylation and glycosylation patterns
Source: Nat Commun. 2018 Nov 7;9:4647. doi: 10.1038/s41467-018-06950-3 (PMC6220181; doi:10.1038/s41467-018-06950-3)
Supplement: Supplementary file 4 — Supplementary Data 1 [file 41467_2018_6950_MOESM4_ESM.pdf]

Supplementary Data 1 (1/4) - Distribution of complex N-glycans

	exact mass	Structures	Organs							
**No.**	theoritical *m/z*	Scheme	*skin [sk]*	*gill [gi]*	*brain [br]*	*heart [he]*	*liver [li]*	*intestine [in]*	*testis [te]*	*ovary [ov]*
**NG1**	1661,6	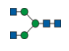				✓		✓	✓	
**NG2**	1824,9	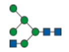	✓							
**NG3**		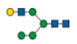	✓							
**NG4**	1835,9	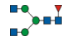						✓	✓	
**NG5**	1865,9	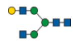						✓		
**NG6**	2029,0	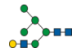	✓	✓						
**NG7**		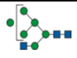	✓	✓						
**NG8**	2040,0	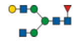		✓	✓				✓	
**NG9**	2070,0	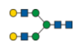	✓					✓	✓	
**NG10**		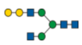						✓		
**NG11**		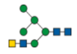	✓						✓	
**NG12**	2081,1	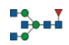	✓	✓	✓	✓	✓	✓	✓	
**NG13**	2244,1	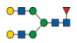	✓	✓	✓			✓	✓	
**NG14**		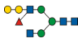	✓	✓				✓	✓	
**NG15**	2257,1	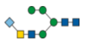								✓
**NG16**	2285,2	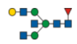	✓	✓		✓	✓	✓	✓	
**NG17**		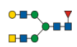	✓						✓	
**NG18**		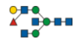			✓			✓		
**NG19**		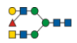								✓
**NG20**	2431,2	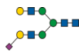		✓						
**NG21**	2448,2	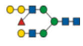		✓						
**NG22**		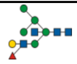		✓						
**NG23**	2459,2	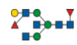		✓	✓					
**NG24**	2461,2	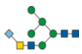								✓
**NG25**	2489,3	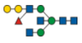		✓		✓	✓	✓		
**NG26**		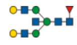	✓	✓		✓		✓		

Supplementary Data 1 (2/4)

No.	theoritical *m/z*	Scheme	*skin [sk]*	*gill [gi]*	*brain [br]*	*heart [he]*	*liver [li]*	*intestine [in]*	*testis [te]*	*ovary [ov]*
**NG27**	2489,3	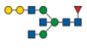		✓		✓		✓		
**NG28**		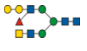	✓			✓				
**NG29**		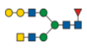	✓							
**NG30**	2605,3	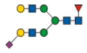	✓	✓	✓	✓		✓		
**NG31**		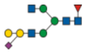			✓					
**NG32**		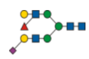		✓	✓	✓		✓		
**NG33**		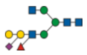	✓	✓		✓				
**NG34**		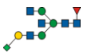						✓		
**NG35**	2622,3	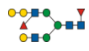		✓						
**NG36**		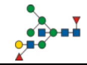		✓						
**NG37**	2635,3	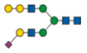		✓						
**NG38**		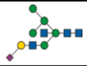		✓						
**NG39**		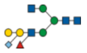		✓						
**NG40**		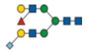		✓						
**NG41**		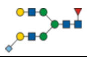		✓						
**NG42**	2663,3	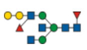	✓	✓		✓	✓	✓	✓	✓
**NG43**		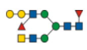	✓							
**NG44**	2693,4	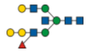				✓				
**NG45**		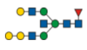				✓				
**NG46**	2809,4	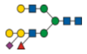	✓	✓		✓		✓		
**NG47**		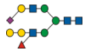	✓	✓	✓	✓		✓		
**NG48**		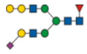			✓					
**NG49**		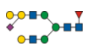			✓			✓		
**NG50**		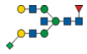	✓					✓		
**NG51**		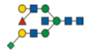						✓		

Supplementary Data 1 (3/4)

No.	theoritical *m/z*	Scheme	*skin [sk]*	*gill [gi]*	*brain [br]*	*heart [he]*	*liver [li]*	*intestine [in]*	*testis [te]*	*ovary [ov]*
**NG52**	2826,4	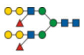							✓	
**NG53**	2850,4	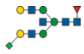	✓	✓						
**NG54**		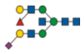		✓						
**NG55**		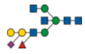	✓	✓						
**NG56**		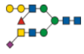	✓							
**NG57**	2867,4	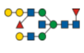	✓	✓		✓		✓	✓	
**NG58**	2904,4	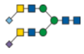								✓
**NG59**	2934,5	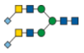								✓
**NG60**	2966,5	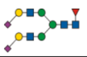			✓					
**NG61**	2983,5	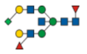						✓		
**NG62**		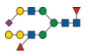		✓				✓		
**NG63**		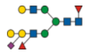		✓						
**NG64**		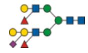						✓		
**NG65**	2996,5	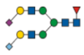			✓					
**NG66**	3000,5	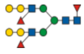							✓	
**NG67**	3013,5	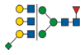						✓		
**NG68**		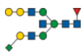						✓		
**NG69**	3054,3	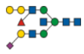		✓					✓	
**NG70**		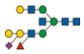		✓					✓	
**NG71**	3071,5	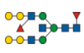						✓	✓	
**NG72**		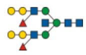				✓		✓	✓	
**NG73**		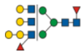						✓		
**NG74**	3129,5	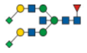						✓		
**NG75**	3170,6	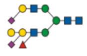		✓						
**NG76**		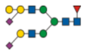			✓					
**NG77**	3187,6	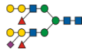	✓	✓		✓	✓	✓	✓	✓
**NG78**	3187,6	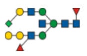						✓		

Supplementary Data 1 (4/4)

No.	theoritical *m/z*	Scheme	*skin [sk]*	*gill [gi]*	*brain [br]*	*heart [he]*	*liver [li]*	*intestine [in]*	*testis [te]*	*ovary [ov]*
**NG79**	3200,6	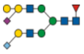			✓					
**NG80**	3200,6	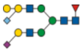			✓					
**NG81**	3217,6	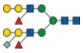		✓						✓
**NG82**		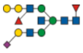		✓		✓		✓	✓	
**NG83**	3228,6	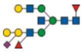		✓				✓		
**NG84**		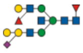						✓		
**NG85**	3245,6	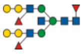	✓	✓		✓	✓	✓	✓	✓
**NG86**	3361,7	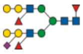				✓				
**NG87**	3374,7	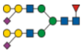			✓					
**NG88**		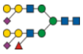				✓				
**NG89**	3548,8	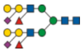	✓	✓	✓	✓	✓	✓	✓	✓
**NG90**	3578,8	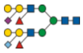	✓	✓	✓	✓	✓	✓	✓	✓
**NG91**	3608,8	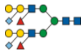	✓	✓	✓	✓	✓	✓	✓	✓
**NG92**	3722,8	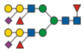	✓							
**NG93**	4359,2	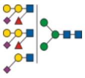		✓						
**NG94**	4737,3	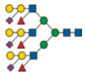		✓						
**NG95**	4739,3	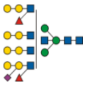						✓		

Supplementary Data 1 - The inclusion criteria of individual molecules in an organ were (1) observation of corresponding MS signal in three independent experiments and (2) confirmation of individual compounds by MS/MS analysis of permethylated glycans. The structural analysis of N-glycans was mostly based on the CID MS/MS fragmentation patterns of permethylated N-glycans in positive mode, in accordance with knowledge of embryonic N-glycans[1–5]. Graphical representation is based on accepted conventions for N-glycans and monosaccharide nomenclature as follows: yellow circle, Gal; yellow square, GalNAc; blue circle, Glc; blue square, GlcNAc; green circle, Man; red triangle, Fuc; purple diamond, Neu5Ac; light blue diamond, Neu5Gc; green diamond, Kdn.[6,7] Core Fucose is *de facto* positioned on reducing GlcNAc residue

on C6 carbon as for all vertebrate *N*-glycans. The interglycosidic bonds between monosaccharides of antennae are represented using the conventional positions as in I, C2 position; ⁄ , C3 position; —, C4 position; \ , C6 position. The respective positions of C3 and C6 branches on internal Man residue of N-glycan core are just indicative.

Supplementary References

1. Guérardel, Y., Chang, L.-Y., Maes, E., Huang, C.-J. & Khoo, K.-H. Glycomic survey mapping of zebrafish identifies unique sialylation pattern. *Glycobiology* **16,** 244–257 (2006).
2. Chang, L.-Y. *et al.* Developmental regulation of oligosialylation in zebrafish. *Glycoconj. J.* **26,** 247–261 (2009).
3. Vanbeselaere, J. *et al.* Mapping the expressed glycome and glycosyltransferases of zebrafish liver cells as a relevant model system for glycosylation studies. *J. Proteome Res.* **11,** 2164–2177 (2012).
4. Hanzawa, K., Suzuki, N. & Natsuka, S. Structures and developmental alterations of N-glycans of zebrafish embryos. *Glycobiology* **27,** 228–245 (2017).
5. Takemoto, T., Natsuka, S., Nakakita, S.-I. & Hase, S. Expression of complex-type N-glycans in developmental periods of zebrafish embryo. *Glycoconj. J.* **22,** 21–26 (2005).
6. *Essentials of Glycobiology*. (Cold Spring Harbor Laboratory Press, 2015).
7. Varki, A. *et al.* Symbol Nomenclature for Graphical Representations of Glycans. *Glycobiology* **25,** 1323–1324 (2015).
