# Supplementary material for: Systems glycomics of adult zebrafish identifies organ-specific sialylation and glycosylation patterns
Source: Nat Commun. 2018 Nov 7;9:4647. doi: 10.1038/s41467-018-06950-3 (PMC6220181; doi:10.1038/s41467-018-06950-3)
Supplement: Supplementary file 5 — Supplementary Data 2 [file 41467_2018_6950_MOESM5_ESM.pdf]

Supplementary Data 2 (1/2) - Distribution of mucin type O-glycans

No	theoritical m/z	Scheme	skin [sk]	gill [gi]	brain [br]	heart [he]	liver [li]	intestine [in]	testis [te]	ovary [ov]
**OG1**	854,4	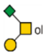						✓		
**OG2**	895,4	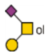	✓	✓	✓	✓	✓	✓	✓	
**OG3**		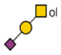				✓	✓	✓		
**OG4**	925,4	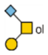		✓		✓				
**OG5**		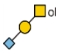				✓				
**OG6**	936,4	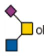		✓						
**OG7**	953,5	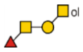	✓	✓				✓		✓
**OG8**	983,0	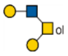				✓		✓	✓	
**OG9**	1141,0	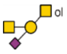							✓	
**OG10**	1174,5	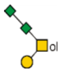						✓		
**OG11**		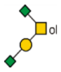						✓		
**OG12**	1215,6	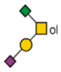						✓		
**OG13**		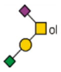						✓		
**OG14**	1239,6	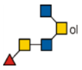								✓
**OG15**	1256,6	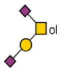	✓	✓	✓	✓	✓	✓	✓	
**OG16**	1286,6	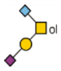		✓	✓	✓		✓		
**OG17**		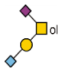	✓	✓	✓	✓		✓		
**OG18**	1314,6	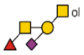	✓		✓			✓	✓	✓
**OG19**	1316,6	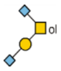	✓			✓				

Supplementary Data 2 (2/2)

No	theoritical m/z	Scheme	skin [sk]	gill [gi]	brain [br]	heart [he]	liver [li]	intestine [in]	testis [te]	ovary [ov]
**OG20**	1344,8	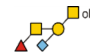	✓		✓	✓	✓	✓	✓	✓
**OG21**		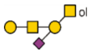			✓	✓				
**OG22**		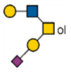			✓	✓				
**OG23**	1361,7	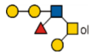	✓			✓	✓	✓	✓	✓
**OG24**		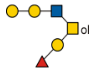	✓			✓				
**OG25**	1374,6	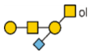	✓			✓				✓
**OG26**		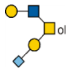				✓				
**OG27**	1617,8	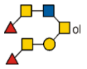	✓	✓						
**OG28**		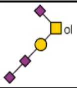	✓		✓	✓		✓	✓	
**OG29**	1647,8	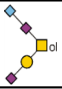			✓					
**OG30**	1722,8	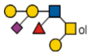				✓		✓	✓	
**OG31**	1752,8	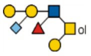				✓		✓	✓	
**OG32**	1791,9	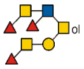		✓						
**OG33**	1979,0	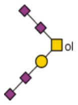			✓					

Supplementary Data 2 - The inclusion criteria of individual molecules in an organ were (1) observation of corresponding MS signal in three independent experiments and (2) confirmation of individual compounds by MS/MS analysis of permethylated glycans. The structural analysis of O-glycans was mostly based on the CID MS/MS fragmentation patterns of permethylated O-glycans in positive mode, in accordance with knowledge of embryonic O-glycans[1–3]. Graphical representation is based on accepted conventions for O-glycans and monosaccharide nomenclature.[4,5] The interglycosidic bonds between monosaccharides are represented using the conventional positions as I, C2 position; ∕ , C3 position; ―, C4 position; \ , C6 position, except for (α2,8) linkages between sialic acids which do not follow any convention.

Supplementary References

1. Guérardel, Y., Chang, L.-Y., Maes, E., Huang, C.-J. & Khoo, K.-H. Glycomic survey mapping of zebrafish identifies unique sialylation pattern. *Glycobiology* **16,** 244–257 (2006).
2. Chang, L.-Y. *et al.* Developmental regulation of oligosialylation in zebrafish. *Glycoconj. J.* **26,** 247–261 (2009).
3. Vanbeselaere, J. *et al.* Mapping the expressed glycome and glycosyltransferases of zebrafish liver cells as a relevant model system for glycosylation studies. *J. Proteome Res.* **11,** 2164–2177 (2012).
4. *Essentials of Glycobiology*. (Cold Spring Harbor Laboratory Press, 2015).
5. Varki, A. *et al.* Symbol Nomenclature for Graphical Representations of Glycans. *Glycobiology* **25,** 1323–1324 (2015).
