# Supplementary material for: Systems glycomics of adult zebrafish identifies organ-specific sialylation and glycosylation patterns
Source: Nat Commun. 2018 Nov 7;9:4647. doi: 10.1038/s41467-018-06950-3 (PMC6220181; doi:10.1038/s41467-018-06950-3)
Supplement: Supplementary file 6 — Supplementary Data 3 [file 41467_2018_6950_MOESM6_ESM.pdf]

## Supplementary Data 3 (1/7) - Distribution of GSLs

| | *Structures* | *exact mass* | *Ceramide* | | *Organs* | | | | | | | |
|---|---|---|---|---|---|---|---|---|---|---|---|---|
| ***No.*** | *Scheme* | *theoritical m/z* | *mass* | *SB, FA* | *skin [sk]* | *gill [gi]* | *brain [br]* | *heart [he]* | *liver [li]* | *intestine [in]* | *testis [te]* | *ovary [ov]* |
| **GL1** | | 806.6 | 548 | d18:1, C16:0 | ✓ | ✓ | | | | | ✓ | ✓ |
| **GL2** | | 808.6 | 550 | d18:0, C16:0 | | | | | | | | ✓ |
| **GL3** | 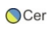 | 836.6 | 578 | t18:0, C16:1 | ✓ | ✓ | | | | | | |
| **GL4** | | 838.6 | 580 | t18:0, C16:0 | ✓ | ✓ | | | | | | |
| **GL5** | | 866,7 | 608 | t18:0, C18:0 | | | | | | | ✓ | |
| **GL6** | | 890.7 | 632 | t18:0, C20:2 | ✓ | | | | | | | |
| **GL7** | | 892.7 | 634 | t18:0, C20:1 | ✓ | | ✓ | | | | | |
| **GL8** | | 894.7 | 636 | t18:0, C20:0 | ✓ | ✓ | | | | | | |
| **GL9** | | 916,7 | 658 | d18:1, 24:1 | ✓ | | ✓ | ✓ | | | | |
| **GL10** | | 918,7 | 660 | d18:1, 24:0 | ✓ | | ✓ | ✓ | | | | |
| **GL11** | | 920,7 | 662 | t18:0, C22:1 | ✓ | | ✓ | ✓ | | | | |
| **GL12** | | 922,7 | 664 | t18:0, C22:0 | | | ✓ | ✓ | | | | |
| **GL13** | | 934,7 | 676 | t18:0, C23:1 | | | | | ✓ | | | |
| **GL14** | | 946.7 | 688 | d18:1, C26:0 | | | ✓ | ✓ | | | | |
| **GL15** | | 948.7 | 690 | t18:0, C24:1 | ✓ | ✓ | ✓ | ✓ | ✓ | | ✓ | |
| **GL16** | | 962,8 | 704 | t18:0, C25:1 | | | | ✓ | | | | |
| **GL17** | | 964,8 | 706 | t18:0, C25:0 | | | | | ✓ | | | |
| **GL18** | | 966,8 | 708 | t18:0, hC23:0 | | | | | | ✓ | | |
| **GL19** | | 976,8 | 718 | t18:0, C26:1 | | | | ✓ | | | | |
| **GL20** | | 978,8 | 720 | t18:0, C26:0 | | | | | | ✓ | | |
| **GL21** | | 980,8 | 722 | t18:0, hC24:0 | | | | | ✓ | ✓ | | |
| **GL22** | | 1004,9 | 746 | t18:1, C28:0 | | | | | | | ✓ | |
| **GL23** | | 1006,9 | 748 | t18:0, C28:0 | | | | | | | ✓ | |
| **GL24** | | 1008,8 | 750 | t18:0, hC26:0 | | | | | ✓ | ✓ | | |
| **GL25** | 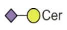 | 1281,9 | 662 | t18:0, C22:1 | | | | ✓ | ✓ | | | |
| **GL26** | | 1295,9 | 676 | t18:0, C23:1 | | | | ✓ | ✓ | | | |
| **GL27** | | 1299,9 | 680 | t18:0, hC20:0 | | | | | | ✓ | | |
| **GL28** | | 1310,0 | 690 | t18:0, C24:1 | | | | ✓ | ✓ | | | |

Supplementary Data 3 (2/7)

| No. | Scheme | theoritical m/z | mass | SB, FA | skin [sk] | gill [gi] | brain [br] | heart [he] | liver [li] | intestine [in] | testis [te] | ovary [ov] |
|---|---|---|---|---|---|---|---|---|---|---|---|---|
| GL29 | 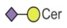 | 1314,0 | 694 | t18:0, hC22:0 | | | | | | ✓ | | |
| GL30 | | 1324,0 | 704 | t18:0, C25:1 | | | | ✓ | ✓ | | | |
| GL31 | | 1328,0 | 708 | t18:0, hC23:0 | | | | | | ✓ | | |
| GL32 | | 1338,0 | 718 | t18:0, C26:1 | | | | ✓ | ✓ | | | |
| GL33 | | 1366,0 | 746 | t18:0, C28:1 | | | | | ✓ | | | |
| GL34 | 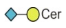 | 1312,0 | 662 | t18:0, C22:1 | | | | ✓ | ✓ | | | |
| GL35 | | 1326,0 | 676 | t18:0, C23:1 | | | | ✓ | | ✓ | | |
| GL36 | | 1329,0 | 680 | t18:0, hC20:0 | | | | | | ✓ | | |
| GL37 | | 1340,0 | 690 | t18:0, C24:1 | | | | ✓ | | | | |
| GL38 | | 1368,0 | 718 | t18:0, C28:1 | | | | | ✓ | | | |
| GL39 | | 1372,0 | 722 | t18:0, hC24:0 | | | | | | ✓ | | |
| GL40 | 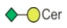 | 1272,9 | 694 | t18:0, hC22:0 | | | | | | ✓ | | |
| GL41 | | 1286,9 | 708 | t18:0, hC23:0 | | | | | | ✓ | | |
| GL42 | | 1299,9 | 720 | t18:0, C26:0 | | | | | | ✓ | | |
| GL43 | | 1301,0 | 722 | t18:0, hC24:0 | | | | | | ✓ | | |
| GL44 | | 1313,0 | 734 | t18:0, C27:0 | | | | | | ✓ | | |
| GL45 | | 1315,0 | 736 | t18:0, hC25:0 | | | | | | ✓ | | |
| GL46 | | 1329,0 | 750 | t18:0, hC26:0 | | | | | | ✓ | | |
| GL47 | 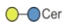 | 1008,7 | 546 | d18:1, C16:1 | | | | ✓ | | ✓ | | |
| GL48 | | 1010,8 | 548 | d18:1, C16:0 | | ✓ | | | ✓ | ✓ | | ✓ |
| GL49 | | 1012,8 | 550 | d18:0, C16:0 | | | | | | | | ✓ |
| GL50 | | 1092,8 | 630 | d18:1, C22:1 | | ✓ | | | | | | |
| GL51 | | 1094,8 | 632 | d18:1, C22:0 | | ✓ | | | | | | |
| GL52 | | 1120,9 | 658 | d18:1, C24:1 | ✓ | ✓ | ✓ | ✓ | | ✓ | | |
| GL53 | | 1122,9 | 660 | d18:1, C24:0 | | ✓ | ✓ | ✓ | | ✓ | | ✓ |
| GL54 | | 1124,9 | 662 | t18:0, C22:1 | | | | | | ✓ | | |
| GL55 | 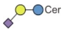 | 1372,0 | 548 | d18:1, C16:0 | ✓ | ✓ | | ✓ | | ✓ | ✓ | |

Supplementary Data 3 (3/7)

| No. | Scheme | theoritical m/z | mass | SB, FA | skin [sk] | gill [gi] | brain [br] | heart [he] | liver [li] | intestine [in] | testis [te] | ovary [ov] |
|---|---|---|---|---|---|---|---|---|---|---|---|---|
| **GL56** | 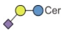 | 1456,0 | 632 | d18:1, C22:0 | | ✓ | | | | ✓ | ✓ | |
| **GL57** | | 1482,0 | 658 | d18:1, C24:1 | ✓ | | | ✓ | | | ✓ | |
| **GL58** | | 1484,0 | 660 | d18:1, C24:0 | ✓ | | | | | | ✓ | |
| **GL59** | | 1544,1 | 720 | t18:0, C26:0 | | ✓ | | | | | | |
| **GL60** | 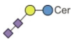 | 1761,1 | 576 | d18:1, C18:0 | | | ✓ | | | | | |
| **GL61** | | 1821,2 | 636 | t18:0, C20:0 | | | | | | ✓ | | |
| **GL62** | | 1903,2 | 718 | t18:0, C26:1 | | | | | | | ✓ | |
| **GL63** | 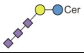 | 2123,3 | 576 | d18:1, C18:0 | | | ✓ | | | | | |
| **GL64** | 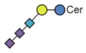 | 2153,3 | 576 | d18:1, C18:0 | | | ✓ | | | | | |
| **GL65** | 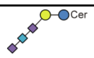 | 2153,3 | 576 | d18:1, C18:0 | | | ✓ | | | | | |
| **GL66** | 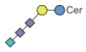 | 2153,3 | 576 | d18:1, C18:0 | | | ✓ | | | | | |
| **GL67** | 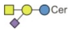 | 1617,1 | 548 | d18:1, C16:0 | ✓ | ✓ | | ✓ | ✓ | ✓ | ✓ | ✓ |
| **GL68** | | 1647,1 | 578 | t18:0, C16:1 | ✓ | ✓ | | | | | ✓ | ✓ |
| **GL69** | | 1701,1 | 632 | d18:1, C22:0 | ✓ | ✓ | | ✓ | | | ✓ | |
| **GL70** | | 1727,2 | 658 | d18:1, C24:1 | | ✓ | | ✓ | ✓ | ✓ | ✓ | ✓ |
| **GL71** | | 1729,2 | 660 | d18:1, C24:0 | ✓ | ✓ | | | | ✓ | ✓ | ✓ |
| **GL72** | | 1757,2 | 688 | d18:1, C26:0 | ✓ | | | | | | ✓ | ✓ |
| **GL73** | | 1759,2 | 690 | t18:0, C24:1 | | | | | | | ✓ | ✓ |
| **GL74** | 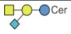 | 1647,1 | 548 | d18:1, C16:0 | | | | ✓ | | ✓ | ✓ | ✓ |
| **GL75** | | 1757,2 | 658 | d18:1, C24:1 | ✓ | | | ✓ | | ✓ | ✓ | |
| **GL76** | | 1759,2 | 660 | d18:1, C24:0 | ✓ | | | ✓ | | ✓ | ✓ | |
| **GL77** |  | 1460,0 | 548 | d18:1, C16:0 | | ✓ | | | | | ✓ | |
| **GL78** | | 1520,0 | 608 | t18:0, C18:0 | | | | | | | ✓ | |
| **GL79** | | 1544,1 | 632 | d18:1, C22:0 | | ✓ | | | | | | |
| **GL80** | | 1604,1 | 692 | t18:0, C24:0 | | ✓ | | | | | | |
| **GL81** | 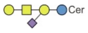 | 1819,1 | 546 | d18:1, C16:1 | | | | | | | | ✓ |
| **GL82** | | 1821,2 | 548 | d18:1, C16:0 | ✓ | ✓ | | | ✓ | ✓ | ✓ | ✓ |

Supplementary Data 3 (4/7)

| No. | Scheme | theoritical m/z | mass | SB, FA | skin [sk] | gill [gi] | brain [br] | heart [he] | liver [li] | intestine [in] | testis [te] | ovary [ov] |
|---|---|---|---|---|---|---|---|---|---|---|---|---|
| GL83 | 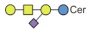 | 1850,2 | 576 | d18:1, C18:0 | | | ✓ | | | | | |
| GL84 | | 1933,3 | 660 | d18:1, C24:0 | ✓ | ✓ | | ✓ | | ✓ | | |
| GL85 | 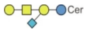 | 1851,2 | 548 | d18:1, C16:0 | | | | | ✓ | | | |
| GL86 |  | 1821,2 | 548 | d18:1, C16:0 | ✓ | ✓ | | | | | ✓ | |
| GL87 | | 1850,2 | 576 | d18:1, C18:0 | | | ✓ | | | | | |
| GL88 | | 1933,3 | 660 | d18:1, C24:0 | | ✓ | | | | | | |
| GL89 | 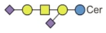 | 2210,3 | 576 | d18:1, C18:0 | | | ✓ | | | | | |
| GL90 | | 2270,4 | 636 | t18:0, C20:0 | ✓ | | | | | | | |
| GL91 | | 2294,5 | 660 | d18:1, C24:0 | ✓ | | | | | | | |
| GL92 | | 2296,4 | 662 | t18:0, C22:1 | ✓ | | | | | | | |
| GL93 | | 2322,5 | 688 | d18:1, C26:0 | ✓ | | | | | | | |
| GL94 | | 2324,5 | 690 | t18:0, C24:1 | ✓ | | | | | | | |
| GL95 | 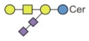 | 2210,3 | 576 | d18:1, C18:0 | | | ✓ | | | | | |
| GL96 | 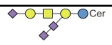 | 2572,5 | 576 | d18:1, C18:0 | | | ✓ | | | | | |
| GL97 | 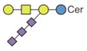 | 2572,5 | 576 | d18:1, C18:0 | | | ✓ | | | | | |
| GL98 | 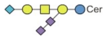 | 2602,2 | 576 | d18:1, C18:0 | | | ✓ | | | | | |
| GL99 | 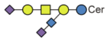 | 2602,2 | 576 | d18:1, C18:0 | | | ✓ | | | | | |
| GL100 | 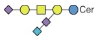 | 2602,2 | 576 | d18:1, C18:0 | | | ✓ | | | | | |
| GL101 | 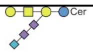 | 2602,2 | 576 | d18:1, C18:0 | | | ✓ | | | | | |
| GL102 | 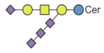 | 2933,7 | 576 | d18:1, C18:0 | | | ✓ | | | | | |
| GL103 | 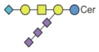 | 2963,7 | 576 | d18:1, C18:0 | | | ✓ | | | | | |
| GL104 | 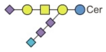 | 2963,7 | 576 | d18:1, C18:0 | | | ✓ | | | | | |
| GL105 | 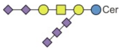 | 3294,8 | 576 | d18:1, C18:0 | | | ✓ | | | | | |
| GL106 | 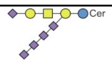 | 3294,8 | 576 | d18:1, C18:0 | | | ✓ | | | | | |
| GL107 | 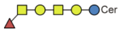 | 1879,2 | 548 | d18:1, C16:0 | | | | | | | ✓ | |
| GL108 | 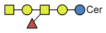 | 1879,2 | 548 | d18:1, C16:0 | | | | | | | ✓ | |
| GL109 | 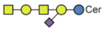 | 2066,3 | 548 | d18:1, C16:0 | | ✓ | | | | | ✓ | ✓ |

# Supplementary Data 3 (5/7)

| No. | Scheme | theoritical m/z | mass | SB, FA | skin [sk] | gill [gi] | brain [br] | heart [he] | liver [li] | intestine [in] | testis [te] | ovary [ov] |
|---|---|---|---|---|---|---|---|---|---|---|---|---|
| **GL110** | 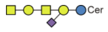 | 2096,3 | 578 | t18:0, C16:1 | | | | | | | ✓ | |
| **GL111** | 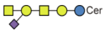 | 2066,3 | 548 | d18:1, C16:0 | ✓ | ✓ | | ✓ | | | ✓ | ✓ |
| **GL112** | | 2096,3 | 578 | t18:0, C16:1 | | | | | | | ✓ | |
| **GL113** | 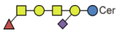 | 2238,4 | 546 | d18:1, C16:1 | | | | | | ✓ | | |
| **GL114** | | 2240,4 | 548 | d18:1, C16:0 | ✓ | ✓ | | | ✓ | ✓ | ✓ | |
| **GL115** | | 2270,4 | 578 | t18:0, C16:1 | ✓ | | | | | | | |
| **GL116** | | 2272,4 | 580 | t18:0, C16:0 | | | | | ✓ | | | |
| **GL117** | | 2350,5 | 658 | d18:1, C24:1 | ✓ | | | ✓ | | | | |
| **GL118** | | 2352,5 | 660 | d18:1, C24:0 | ✓ | | | ✓ | ✓ | | ✓ | |
| **GL119** | | 2382,5 | 690 | t18:0, C24:1 | | | | | | | ✓ | |
| **GL120** | 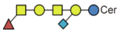 | 2270,4 | 548 | d18:1, C16:0 | | | | | ✓ | | ✓ | |
| **GL121** | | 2382,5 | 660 | d18:1, C24:0 | | | | | | | ✓ | |
| **GL122** | 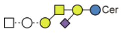 | 2270,4 | 548 | d18:1, C16:0 | | | | | | | ✓ | |
| **GL123** | 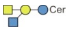 | 1501,0 | 548 | d18:1, C16:0 | | | | | | | ✓ | ✓ |
| **GL124** | 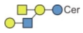 | 1705,1 | 548 | d18:1, C16:0 | | | | | | | ✓ | |
| **GL125** | | 1817,2 | 660 | d18:1, C24:0 | | | | | | | | ✓ |
| **GL126** | | 1819,2 | 662 | t18:0, C22:1 | | | | | | | | ✓ |
| **GL127** | | 1821,2 | 664 | t18:0, C22:0 | | | | | | | | ✓ |
| **GL128** | 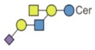 | 2066,3 | 548 | d18:1, C16:0 | | ✓ | | | | | ✓ | ✓ |
| **GL129** | | 2096,3 | 578 | t18:0, C16:1 | | | | | | | ✓ | |
| **GL130** | 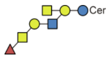 | 2124,3 | 548 | d18:1, C16:0 | | | | | | | ✓ | |
| **GL131** | | 2156,3 | 580 | t18:0, C16:0 | | ✓ | | | | | | |
| **GL132** | | 2180,3 | 604 | d18:1, C18:0 | ✓ | | | | | | | |
| **GL133** | | 2186,3 | 610 | t18:0, hC16:0 | ✓ | | | | | | | |
| **GL134** | | 2212,4 | 636 | t18:0, C20:0 | | ✓ | | | | | | |
| **GL135** | | 2226,3 | 650 | t18:0, C21:0 | | ✓ | | | | | | |
| **GL136** | | 2240,3 | 664 | t18:0, C22:0 | ✓ | ✓ | | | | | | |

Supplementary Data 3 (6/7)

| No. | Scheme | theoritical m/z | mass | SB, FA | skin [sk] | gill [gi] | brain [br] | heart [he] | liver [li] | intestine [in] | testis [te] | ovary [ov] |
|---|---|---|---|---|---|---|---|---|---|---|---|---|
| GL137 | 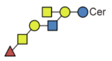 | 2254,5 | 678 | t18:0, C23:0 | | | | | | ✓ | | ✓ |
| GL138 | | 2256,4 | 680 | t18:0, hC20:0 | | | | | | ✓ | | |
| GL139 | | 2270,4 | 694 | t18:0, hC22:0 | | | | | ✓ | | | |
| GL140 | | 2284,4 | 708 | t18:0, hC23:0 | | | | | ✓ | | | |
| GL141 | | 2296,5 | 720 | t18:0, C26:0 | ✓ | | | | | | | |
| GL142 | | 2298,5 | 722 | t18:0, hC24:0 | | | | | ✓ | | | |
| GL143 | | 2310,5 | 734 | t18:0, C27:0 | ✓ | | | | | | | |
| GL144 | | 2312,5 | 736 | t18:0, hC25:0 | | | | | ✓ | | | |
| GL145 | | 2324,5 | 748 | t18:0, C28:0 | ✓ | | | | | | | |
| GL146 | | 2326,5 | 750 | t18:0, hC26:0 | | | | | ✓ | | | |
| GL147 | 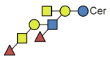 | 2330,4 | 580 | t18:0, C16:0 | | ✓ | | | | | | |
| GL148 | | 2386,5 | 636 | t18:0, C20:0 | | ✓ | | | | | | |
| GL149 | 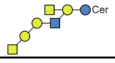 | 2186,4 | 580 | t18:0, C16:0 | | | | | | | | ✓ |
| GL150 | 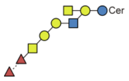 | 2535,5 | 580 | t18:0, C16:0 | | | | | | | | ✓ |
| GL151 | | 2644,7 | 690 | t18:0, C24:1 | | | | | | | | ✓ |
| GL152 | | 2674,7 | 720 | t18:0, C26:0 | | | | | | | | ✓ |
| GL153 | 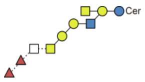 | 2779,7 | 580 | t18:0, C16:0 | | | | | | | | ✓ |
| GL154 | | 2891,8 | 692 | t18:0, C24:0 | | | | | | | | ✓ |
| GL155 | 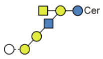 | 2254,3 | 690 | t18:0, C24:1 | | | | | | | | ✓ |
| GL156 | | 2284,3 | 720 | t18:0, C26:0 | | | | | | | | ✓ |
| GL157 | 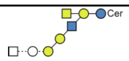 | 2390,5 | 580 | t18:0, C16:0 | | | | | | | | ✓ |
| GL158 | 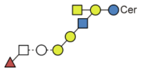 | 2564,6 | 580 | t18:0, C16:0 | | | | | | | | ✓ |
| GL159 | | 2646,6 | 662 | t18:0, C22:1 | | | | | | | | ✓ |
| GL160 | | 2648,6 | 664 | t18:0, C22:0 | | | | | | | | ✓ |
| GL161 | | 2674,7 | 690 | t18:0, C24:1 | | | | | | | | ✓ |
| GL162 | | 2704,7 | 720 | t18:0, C26:0 | | | | | | | ✓ | ✓ |

Supplementary Data 3 (7/7)

| No. | Scheme | theoritical m/z | mass | SB, FA | skin [sk] | gill [gi] | brain [br] | heart [he] | liver [li] | intestine [in] | testis [te] | ovary [ov] |
|---|---|---|---|---|---|---|---|---|---|---|---|---|
| **GL163** | 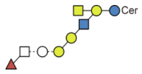 | 2732,8 | 748 | t18:0, C28:0 | | | | | | | ✓ | |
| **GL164** | 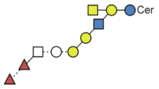 | 2738,6 | 580 | t18:0, C16:0 | | | | | | | | ✓ |
| **GL165** | | 2808,7 | 650 | t18:0, C21:0 | | | | | | | | |
| **GL166** | | 2848,7 | 690 | t18:0, C24:1 | | | | | | | | ✓ |
| **GL167** | | 2878,8 | 720 | t18:0, C26:0 | | | | | | | | ✓ |
| **GL168** | | 2904,8 | 746 | t18:0, C28:1 | | | | | | | | ✓ |
| **GL169** | 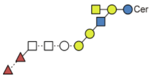 | 2983,8 | 580 | t18:0, C16:0 | | | | | | | | ✓ |
| **GL170** | | 3095,9 | 692 | t18:0, C24:0 | | | | | | | | ✓ |
| **GL171** | 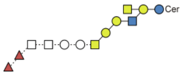 | 3545,1 | 692 | t18:0, C24:0 | | | | | | | | ✓ |
| **GL172** | 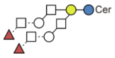 | 2747,7 | 548 | d18:1, C16:0 | | | | | | | ✓ | |

**Supplementary Data 3 -** The inclusion criteria of individual molecules in an organ were (1) observation of corresponding MS signal in three independent experiments and (2) confirmation of individual compounds by MS/MS analysis of permethylated glycans. The sequence of carbohydrate moieties and the nature of ceramides were established by CID MS/MS fragmentation of permethylated GSLs in positive mode. Values in "mass" column correspond to the diagnostic Z fragmentation ions for the methylated ceramide moiety. Most probable combinations of sphingoid bases and fatty acids based on composition and Z fragment ions are provided in the "SB, FA" column. Graphical representation is based on accepted conventions for GSLs and monosaccharide nomenclature.[1,2] The interglycosidic bonds between monosaccharides are represented using the conventional positions as I, C2 position; ⁄ , C3 position; —, C4 position; \ , C6 position, except for ($\alpha$2,8) linkages between sialic acids which do not follow any convention. Whenever linkages could not be deduced from specific CID MS/MS fragmentation pattern and literature, they are shown as dotted lines.

**Supplementary References**


1. *Essentials of Glycobiology*. (Cold Spring Harbor Laboratory Press, 2015).
2. Varki, A. *et al.* Symbol Nomenclature for Graphical Representations of Glycans. *Glycobiology* **25,** 1323–1324 (2015).
