# Supplementary material for: Systems glycomics of adult zebrafish identifies organ-specific sialylation and glycosylation patterns
Source: Nat Commun. 2018 Nov 7;9:4647. doi: 10.1038/s41467-018-06950-3 (PMC6220181; doi:10.1038/s41467-018-06950-3)
Supplement: Supplementary file 7 — Supplementary Data 4 [file 41467_2018_6950_MOESM7_ESM.pdf]

Supplementary Data 4 (1/3) - Distribution of carbohydrate moieties of GSLs

	Structures	***Organs***							
**No.**	*Scheme*	*skin [sk]*	*gill [gi]*	*brain [br]*	*heart [he]*	*liver [li]*	*intestine [in]*	*testis [te]*	*ovary [ov]*
**G1**	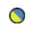	✓	✓	✓	✓	✓	✓	✓	✓
**G2**					✓	✓	✓		
**G3**					✓	✓	✓		
**G4**							✓		
**G5**		✓	✓	✓	✓	✓	✓	✓	✓
**G6**	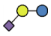	✓	✓	✓	✓		✓	✓	
**G7**	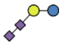			✓			✓	✓	
**G8**	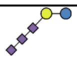			✓					
**G9**	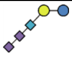			✓					
**G10**	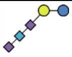			✓					
**G11**	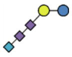			✓					
**G12**	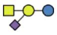	✓	✓		✓	✓	✓	✓	✓
**G13**	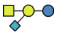	✓	✓		✓		✓	✓	✓
**G14**			✓					✓	
**G15**	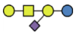	✓	✓	✓	✓	✓	✓	✓	✓
**G16**	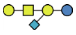					✓			
**G17**		✓	✓	✓				✓	
**G18**	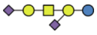	✓		✓					
**G19**	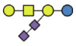			✓					
**G20**	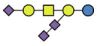			✓					
**G21**	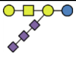			✓					
**G22**	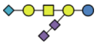			✓					
**G23**	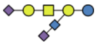			✓					
**G24**	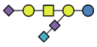			✓					
**G25**	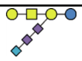			✓					
**G26**	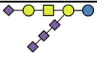			✓					
**G27**	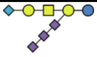			✓					
**G28**	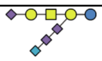			✓					

Supplementary Data 4 (2/3)

No.	Scheme	skin [sk]	gill [gi]	brain [br]	heart [he]	liver [li]	intestine [in]	testis [te]	ovary [ov]
G29	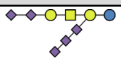			✓					
G30	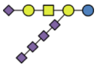			✓					
G31	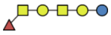							✓	
G32	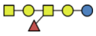							✓	
G33	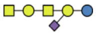		✓					✓	✓
G34	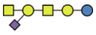	✓	✓		✓			✓	✓
G35	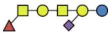	✓	✓		✓	✓	✓	✓	
G36	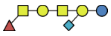					✓		✓	
G37	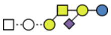							✓	
G38	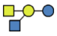							✓	✓
G39	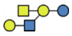							✓	✓
G40	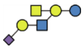		✓					✓	✓
G41	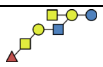	✓	✓			✓	✓	✓	✓
G42	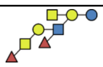		✓						
G43	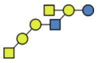								✓
G44	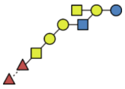								✓
G45	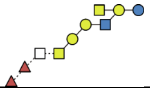								✓
G46	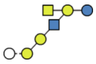								✓
G47	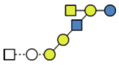								✓
G48	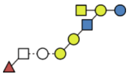							✓	✓
G49	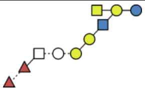								✓
G50	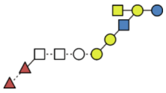								✓

Supplementary data 4 (3/3)

No.	Scheme	skin [sk]	gill [gi]	brain [br]	heart [he]	liver [li]	intestine [in]	testis [te]	ovary [ov]
G51	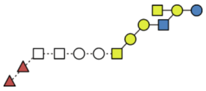								✓
G52	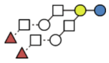							✓	

Supplementary Data 4 - Distribution of carbohydrate moieties of GSLs among the eight screened adult organs, compiled from identification structural analysis of intact GSLs (Supplementary Data 3).
