# Supplementary material for: Systems glycomics of adult zebrafish identifies organ-specific sialylation and glycosylation patterns
Source: Nat Commun. 2018 Nov 7;9:4647. doi: 10.1038/s41467-018-06950-3 (PMC6220181; doi:10.1038/s41467-018-06950-3)
Supplement: Supplementary file 8 — Supplementary Data 5 [file 41467_2018_6950_MOESM8_ESM.pdf]

Supplementary Data 5 - Oligonucleotide sequences

Zebrafish glycosyltransferases	GenBank accession number of ZF genes	Primer sequence<br>Forward<br>Reverse	PCR product size (bp)	Ref.
Actine	AF025305	5'-GTTGGTATGGGACAGAAAGA<br>5'-GGCGTAACCCTCGTAGAT	378	1
st3gal1A	AJ864512	5'-CTCTGGGAACATCTTGGG<br>5'-CGGTTGATGGGTAACTGC	368	2
st3gal1C	AM287261	5'-AAGGACAGCGACTACAGA<br>5'-TGAAGGAACTAATGAGCC	331	2
st3gal1D	AM287262	5'-CCGTATGATCTACCCTGAG<br>5'-TCCATCTTTATCTGCTCC	326	2
st3gal2	AJ783741	5'-CCCACTGTAGGGTATGAA<br>5'-TACGTGTAGAGCGAAGAA	324	2
st3gal8	AJ783740	5'-GGTGGACAGAGCGTCATG<br>5'-GTCAATTAACGGCAGGGTG	371	2
st3gal3	AJ626821	5'-GCAATGGCGGTATCCTTA<br>5'-GGCCTTCACCAACTTTCG	605	2
st3ga3-r	AJ626820	5'-AACCTAAGTAAAGGCGAGTG<br>5'-ACGAGGCTTCCTGTATGA	607	2
st3gal4	AJ744809	5'-AGAGGGTCAGTAAGGGTTT<br>5'-GATTCAGATCGTGGTAGGA	300	2
st3gal5	AJ619960	5'-TGGCTGTGATATTCAAGTC<br>5'-TGTAGGGATGTTCTGGTC	236	2
st3gal7	AJ783742	5'-TCCCACATACAGGAATACG<br>5'-CAGTCTCGGCACTAACATC	424	2
st6gal1	AJ744801	5'-TTCGGGATGCTGATGTTT<br>5'-ATAGAGTAATGGGTGGTAGG	896	3
st6gal2	AJ627627	5'-GGGTGGTGATGAAGGTAGA<br>5'-GTGCAGGTTGAGGGTGTA	628	3
st6gal2-r	FN550105	5'-CTCAACCTACTCTCAGCAT<br>5'-TCGCTGCAGTTTTGCACT	299	3
st6galnac1A	AM287259	5'-GGAGGATGTCGGAAACAGAA<br>5'-TGTTGCGAATGTAACGAAGG	293	This study
st6galnac1B	HE590885	5'-GCCAGTTGCTGCAAGTACACCCA<br>5'-TCTGACCACCCACCACCGCT	503	This study
st6galnac2A	AJ634459	5'-AACTCAGTCCAGCAATCA<br>5'-TGTAGACGGCCTGTAAATT	906	This study

st6galnac3	AJ620947	5’-CACAAAGCCTCCAAACCT<br>5’-TGCGAGCCTGTGGGTAAT	391	This study
st6galnac4	AJ868430	5’-CCAGGGATATGTGAGGATT<br>5’-ACGGAGGATAAACGAAGC	740	This study
st6galnac5A	AJ646874	5’-GCCAGGACACTTTCTTTAT<br>5’-CTGCTCATGGGTGACATA	377	This study
st6galnac5B	XM686955	5’-CAGCGTCATGGAGTTGCC<br>5’-CGCACTCTGCCTGATCGA	295	This study
st6galnac6	AJ646883	5’-AAGCAACTTCCGTCAAATC<br>5’-GCACTCGTCTGGACCTCT	673	This study
st8sia1	AJ715535	5’-TTGCGGTTACTAAGGAGA<br>5’-ACGAAAGATTTGCGGGAC	345	1
st8sia2	AY055462	5’-GACTCGCACGACTTTGTT<br>5’-TGGTTGGTCAGCCAGTAA	335	1
st8sia3	AJ715543	5’-GGTTCGGGTGGTCAGTGTT<br>5’-CGCTGTTCTTGGTCAGGGT	341	This study
st8sia4	AJ715545	5’-TCTTGACTTGGGAGTTGG<br>5’-TCTGACCGCAATCCTACA	366	1
st8sia5	AJ715546	5’-TGGCAAGAACTACATCAA<br>5’-AAAGTCAGAAGCGTCAAT	358	This study
st8sia6	AJ715551	5’-TGTCTATGATGGCGAAAG<br>5’-TGACCGTATGAATGAAGG	333	1
st8sia7	AM287257	5’-TTTCCTGGTGGTCCTGAT<br>5’-GGTGCGTCTACTGTTGGTT	345	1
Neu1	NM_001044909	5’-GCAGCCCCTCCAGCACGATG<br>5’-GGGCAGAGACTCGCCTCCGT	419	4
Neu3.1	EF107693	5’-GACTGTGGCGTCACCTGGCA<br>5’-CGGGCGGCTCCAACCAGAAG	408	4
Neu3.2	EF107692	5’-AGGGATGGTGTTGTCGAGTGGG<br>GCCCAGTTCTTCTCCTGCTTACCG	285	4
Neu3.3	EF107698	5’-GCCTCAAACTTGGTGCTGCTGT<br>5’-GCTCCAGGTTTTGCCAGCGT	400	4
Neu3.4	EF107696	5’-ACCTCTTGGGGTCTCTGAACGG<br>5’-GCAGCAGCATAAAGTGCAGACAGG	262	4
Neu3.5	NM_001109733	5’-AGTGGGTCAGTGGTCAACAGGTG<br>5’-TGCAAGTGCATGCGATGTGGGT	383	4
Neu4	EF116275	5’-ACCAGGGGGACACCTGGAGC<br>5’-AGCTTGCACCCGGTAGCCTG	362	4

CMAS1	JQ015186	5’-CGCGGCGTCAGGACTGGAGTG<br>5’-GGACAGCCCATCCTCTCAGACAGT	427	5
CMAS2	JQ015187	5’-TCCGACTGCGACCCGAGGTG<br>5’-AGGCGATCCTGCCCAGCTCA	321	5
CMAH	NM_001002192	5’-ACCCGTGGCTCACAGGACCA<br>5’-CTGCCACACACCGAACGGCA	275	This study

Supplemental Data 5 - Oligonucleotide sequences and expected amplicon sizes used for PCR detection of actin, sialyltransferases, sialidases, CMP-sialic acid synthases (CMAS) and CMP-sialic acid hydroxylases (CMAH). Accession numbers in GenBank are indicated. data are presented in Figure 7 and Supplementary Figure 7.

Supplementary References

1. Vanbeselaere, J. *et al.* Mapping the expressed glycome and glycosyltransferases of zebrafish liver cells as a relevant model system for glycosylation studies. *J. Proteome Res.* **11,** 2164–2177 (2012).
2. Petit, D. *et al.* Integrative view of α2,3-sialyltransferases (ST3Gal) molecular and functional evolution in deuterostomes: significance of lineage-specific losses. *Mol. Biol. Evol.* **32,** 906–927 (2015).
3. Petit, D. *et al.* Molecular phylogeny and functional genomics of beta-galactoside alpha2,6-sialyltransferases that explain ubiquitous expression of st6gal1 gene in amniotes. *J. Biol. Chem.* **285,** 38399–38414 (2010).
4. Manzoni, M. *et al.* Molecular cloning and biochemical characterization of sialidases from zebrafish (Danio rerio). *Biochem. J.* **408,** 395–406 (2007).
5. Schaper, W. *et al.* Identification and biochemical characterization of two functional CMP-sialic acid synthetases in Danio rerio. *J. Biol. Chem.* **287,** 13239–13248 (2012).
